# Global, regional, and national burden of diet high in processed meat from 1990 to 2019: a systematic analysis from the global burden of disease study 2019

**DOI:** 10.3389/fnut.2024.1354287

**Published:** 2024-02-13

**Authors:** Feng-Xia Wang, Xiang-Hua Kong, Zhe Guo, Lu-Xia Li, Shu Zhang

**Affiliations:** Department of Urology, Shanxi Bethune Hospital, Shanxi Academy of Medical Science, Tongji Shanxi Hospital, Third Hospital of Shanxi Medical University, Taiyuan, China

**Keywords:** diet high in processed meat, dietary pattern, disability-adjusted life year, GBD 2019, mortality, summary exposure value

## Abstract

**Objective:**

The objective of this study is to explore the prevalence and attributable burden of diet high in processed meat (DHIPM) in global, regional, and national level due to the burden caused by unhealthy dietary pattern worldwide.

**Design:**

Cross-sectional study.

**Materials and design:**

All the data involved in this research were obtained from Global Burden of Diseases Study 2019. DisMod-MR 2.1, a Bayesian meta-regression tool, was used to estimate the prevalence, which was measured by summary exposure value (SEV) and attributable burden of DHIPM. The Spearman rank order correlation method was performed to measure the correlation between sociodemographic index (SDI) and the prevalence as well as attributable burden. The estimated annual percentage change (EAPC) was calculated to demonstrate the temporal trends.

**Results:**

Globally, there were 304.28 thousand deaths and 8556.88 disability-adjusted life years (DALYs) caused by DHIPM in 2019 and increased by 34.63 and 68.69%, respectively. The prevalence had decreased slightly from 1990 to 2019, however increased in most regions and countries, especially in middle SDI regions, despite the implicitly high prevalence in high SDI regions. Countries with higher SDI values were facing higher prevalence and attributable burden of DHIPM while developing countries were observed with severer temporal trends. Compared with women, men had suffered from lower exposure level however graver attributable burden of DHIPM in the past three decades.

**Conclusion:**

The progress of continuous urbanization allowed increasingly severe prevalence and attributable burden of DHIPM, thus the challenge to alleviate this trend was acute. Effective measures such as education on beneficial dietary pattern and supplement on healthy food were urgently required, especially in developing regions and countries.

## Introduction

According to a research on the global burden of diseases published in the Lancet, diet was classified as the top risk factor for a reduction in global disability-adjusted life years (DALYs) (1). Compared with diet habits in the eastern population, the food consumption in Western countries was characterized by a higher percentage of refined cereals and processed and ultra-processed food and an insufficient proportion of vegetables, fruits, vitamin, and mineral content (2, 3). Growing evidence showed that a significant association between the diet pattern in eastern population and the incidence and mortality of non-communicable diseases (4–6) was considered to possess seven of the top ten causes of mortality worldwide according to the 2019 Global Health Estimates (7). Diet high in red meat and processed meat was an apparent characteristic of Western diet pattern; however, previous systematic reviews had demonstrated positive associations between red meat consumption and all-cause mortality and cardiovascular mortality and stroke (8–11). In addition, high consumption in processed meat was considered associated with the mortality of all-cause mortality, cardiovascular mortality, stroke, and coronary heart disease (9–12).

To alleviating a huge increase in global meat consumption in the past decades, the world meat production had increased from 234 million tonnes in 2010 to 337 million tonnes in 2019, which had expanded for almost 44%, while the world meat production in 1960 was only 70 million tonnes (13). Moreover, it was reported that more than 60% of this increase were contributed by red meat and processed meat (13). Although it was universally accepted that as an important source of proteins, essential amino acids, minerals (such as iron, potassium, and zinc), vitamins (including B12), and micronutrients for human bodies (14), moderate intake of red meat was absolutely beneficial and recommended while excessive consumption of red meat was proven harmful to the environment and health (15). However, recommendations on processed intake were not as clear as red meet and varied from the guidelines (16–19), although the consumption of processed meat was considered “carcinogenic” to humans (20).

Processed meat was defined as any type of meat that underwent treatment such as salting, fermentation, smoking, and other processes, enhancing the flavor or extending its shelf life (including ham, salami, frankfurters, and turkey blanquette) (20). Compared with unprocessed meat, processed meat was characterized by higher content in solidum, saturated fat, heterocyclic amines and polycyclic aromatic hydrocarbons, which were proven to be carcinogenic and genotoxic (15); moreover, the ratio of the increased nitrites to nitrates in processed meat associated with the rise in cardiovascular diseases, diabetes mellitus, and gastrointestinal tract cancer (21).

The prevalence and attributable burden of diet high in red meat had been well demonstrated in a previously published article (22); however, prior findings on the epidemiology of diet high in processed meat (DHIPM) were based on finite nation (21) or limited endpoint disease (23, 24), thus the prevalence and attributable burden of DHIPM had not been well clarified on a global scale yet. The Global Burden of Diseases, Injuries, and Risk Factors Study 2019 (GBD 2019) systematically reviewed and combined the risk data from 84 risk factors, therefore offering an opportunity to explore the epidemiology of DHIPM at the global, regional, and national levels (25, 26). GBD 2019 defined DHIPM as one of the 84 risk factors that were associated with communicable, non-communicable, and malignant diseases. The data of summary exposure value (SEV), mortality and DALYs of DHIPM in globe, and 23 regions and 204 countries and territories from 1990 to 2019 were extracted to evaluate the trends of prevalence and attributable burden of DHIPM. The correlations between the epidemiology of DHIPM and sex, age, and disparities in economic development were also investigated to demonstrate a comprehensive and precise assessment on the health burden of DHIPM, thus raising the awareness of policymakers and the public to alleviate the burden globally.

## Materials and designs

### Data source and definition

All the data involved in this research were obtained from the GBD study 2019.^1^ Previously published articles had well demonstrated the methodology of data inputting, mortality estimation, and modeling for GBD 2019; moreover, the final data of every disease, injury, and risk factor which can be associated with location, year, and age groups had also been comprehensively reviewed (25, 26). In this study, we aimed to explore the prevalence and associated burden of DHIPM in global, regional, and nation levels from 1990 to 2019. Diet high in processed meat was defined as any intake (in grams per day) of meat preserved by smoking, curing, salting, or addition of chemical preservatives on the basis of the parent GBD risk factor study (25). In GBD 2017, the dietary data adopted in the models came from multiple sources, including nationally and subnationally representative nutrition surveys, household budget surveys, accounts of national sales from the Euromonitor, and availability of data from the United Nations Food and Agriculture Supply and Utilization Accounts. However, GBD 2019 acquired new dietary recall sources from a literature search of PubMed and new sources from the IHME GHDx yearly known survey series updates in our models. GBD 2019 adopted a comparative risk factor assessment framework to estimate the risk factors, which included six steps: identification of risk outcome pairs; exposure estimation; relative risk (RR) estimation; determination of the theoretical minimum-risk exposure level; estimation of SEV, and the attributable burden. The specific methodology to model and estimate all risk factors had been introduced in previous parent GBD studies (25, 27, 28). Thus, we summarized the specific methods for these steps of DHIPM.

### Risk-outcome pairs

GBD 2019 contained risk-outcome pairs which meet the World Cancer Research Fund (WCRF) grades of convincing or probable evidence from 2010 and defined ischemic heart diseases (IHD), diabetes mellitus, and colon and rectum cancer as the disease endpoint of DHIPM (25).

### Relative risk

GBD 2019 utilized the estimation of the RR to the outcomes to serve as a function of exposure to risk factors for each risk-outcome pair. The GBD study collected and performed meta-analyses of RRs from published systematic reviews, and 81 new systematic reviews were added in GBD 2019. Using the sources identified by these searches, GBD 2019 incorporated the most recent epidemiological evidence, assessing the relationship between diet high in processed meat and related outcomes in relative risk analysis (25).

### Exposure estimation

To estimate the distribution of risk exposure, GBD 2019 investigated household surveys, censuses, published studies, and governmental data to estimate the mean levels of risk exposure. Then, GBD 2019 applied spatiotemporal Gaussian process regression (ST-GPR), a non-linear model, to estimate the mean exposure and standard deviation of each risk factor by age, sex, country, and year (26, 28). In this model, the time window was set to 10 years for fitting data, and age was divided into 0, 10, 20, 30, 40, 50, 60, 70, 80, 90, and 100 years. The minimum coefficient of variation was 0.1 for global, 0.06 for super regions, and 0.08 for other region level.

### Theoretical minimum-risk exposure level

Theoretical minimum-risk exposure level (TMREL) was defined as the theoretically possible risk exposure that minimizes the risk to the exposed population. TMREL was regarded as 0 for harmful dietary risk factors with monotonically increasing risk functions and was measured as the 85th percentile of exposure levels across cohort studies or meta-analyses for protective dietary risk factors with monotonically decreasing risk functions.

### Population-attributable fractions

GBD 2019 defined population-attributable fractions (PAF) as the percentage of disease burden that could be alleviated when TMREL exposure to a specific risk factor was achieved. The PAF of DHIPM was calculated according to the following formula: $PAF=\frac{\int_{x=l}^{m}\;RR\left(x\right)P\left(x\right)dx-RR\left(x\right)\mathrm{TRMEL}}{\int_{x=l}^{m}\;RR\left(x\right)P\left(x\right)dx}$ , in this formula, l stood for minimum exposure level, m indicated the maximum exposure level, RR (x) represented the relative risks at exposure level x, TRMEL mean the counterfactual exposure level, and P (x) was on behalf of the current exposure level. Moreover, we calculated all the above variables according to the combination of other important covariates, which included age, sex, location, and year.

### Summary exposure values

The GBD study measured the prevalence of risk factors by the SEV that was weighted by the relative risk. As for the relative risk, zero indicated that there was not excess risk for the population while one stood for that the population was facing the highest level of risk. In this study, SEV, which mean the weighted prevalence of DHIPM at the regional and national levels, varied from 0 to 100, in which 0 indicated that all the people were at minimum prevalence and 100 indicated that all the people were at maximum prevalence. All the reported SEVs in this study were age-standardized by age, and the decline in age-standardized SEV stood for the reduction in prevalence of DHIPM and vice versa.

### Sociodemographic index

In this study, the prevalence and burden of DHIPM were calculated in coordinate with a country-level development metric: sociodemographic index (SDI) (29), which was a composite metric that merged by three separate indicators: (1) lag-distributed income *per capita*; (2) average educational attainment for people aged 15 years and older; (3) the total fertility rate (in people aged <25 years). According to the above criteria, 204 countries and territories worldwide were divided into five groups: low SDI (<0.45), low-middle SDI (≥0.45 and <0.61), middle SDI (≥0.61 and <0.69), high-middle SDI (≥0.69 and <0.80), and high SDI (≥0.80).

### Statistical analysis

In this study, we presented age-standardized SEV, mortality rate (ASMR), DALYs rate (ASDR), and their 95% uncertain intervals (95% UI) to evaluate and compare the prevalence and burden among regions and countries with distinct age structure and demographic traits. We reported all metrics with 95% UI, and the ASMR and ASDR of DHIPM were exhibited per 100,000. Moreover, we calculated the estimated annual percentage change (EAPC) on the basis of age-standardized rates in every year from 1990 to 2019, to demonstrate the trends of prevalence and burden of DHIPM with time. In brief, a linear correlation was determined between the natural logarithm of ASMR or ASDR and time, i.e., y = α + βx + ε, in which x stood for year and y indicated ln (rates), then EAPC and its 95% confidence interval (95% CI) were calculated by the following formula: EAPC = 100* (e^β −1). The ASMR or ASDR was considered to increase with time when the lower boundary of 95% CI is positive; on the contrary, if upper lower boundary of 95% CI is negative, ASMR or ASDR was thought to have a downward trend over the period. The expected values of age-standardized SEV, ASMR, and ASDR within every SDI unit were estimated by Gaussian process regression with a Loess smoother; otherwise, the correlation between the SDI and age-standardized SEV, ASMR, and ASDR was determined by Spearman’s rank order correlation. *p*-value < 0.05 was considered statistically significant in this study, and all the statistical analyses were performed on R software (version 4.0.5).

## Results

### Global and regional prevalence of DHIPM

**Figure 1:**
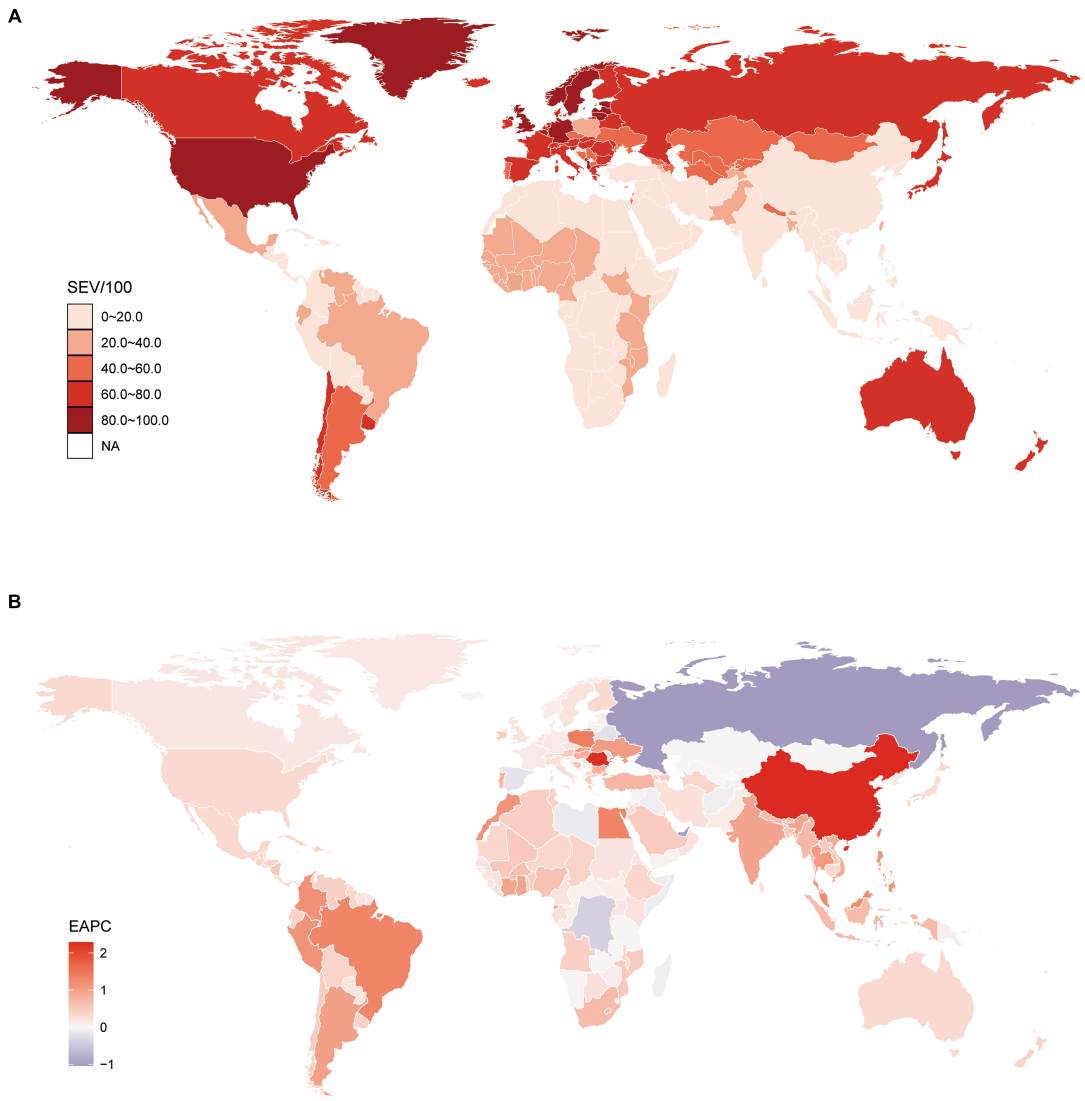
Global exposure to diet high in processed meat. **(A)** Age standardized SEV of diet high in processed meat, for both sexes in 204 countries and territories in 2019. **(B)** The EAPC in age standardized SEV of diet high in processed meat, for both sexes from 1990 to 2019, in 204 countries and territories. SEV, summary exposure value; EAPC, estimated annual percentage change.

The prevalence of DHIPM was demonstrated by SEV according to the GBD 2019, and Table 1 shows the prevalence of DHIPM in 1990, 2000, 2010, and 2019 and the change in the trends of DHIPM in 1990–2010 and 1990–2019, respectively. Globally, the SEVs of DHIPM in both sexes, males and females, were 29.81 (95% UI, 19.04 to 43.32), 28.91 (95% UI, 18.50 to 41.82), and 32.12 (95% UI, 21.74 to 44.62), respectively. At the regional level, high-income North America was observed to own the highest age-standard SEV of DHIPM (83.72; 95% UI, 65.79 to 97.62), followed by Western Europe (78.23; 95% UI, 56.08 to 96.39) and Australasia (74.26; 95% UI, 48.27 to 96.59), while the lowest three age-standard SEVs were found in Southeast Asia (9.57; 95% UI, 4.57 to 21.41), Oceania (9.98; 95% UI, 4.33 to 20.98), and Andean Latin America (11.98; 95% UI 5.5 to 23.83) (Table 1). At the national level, countries with higher age-standard SEV were mainly located in high-income North America, Western Europe, Australasia, and Eastern Europe (Figure 1A); among them, Lithuania possessed the highest age-standard SEV (93.26; 95% UI, 80.98 to 100), the following two countries were Norway (92.59; 95% UI, 83.41 to 99.94) and Latvia (91.69; 95% UI, 77.69 to 100), and it was noteworthy that all of these three countries were located in Europe. The opposite was that countries in South Asia, Southeast Asia, East Asia, and sub-Saharan Africa were observed with a relatively prevalence of DHIPM, Vietnam was observed to have the lowest age-standard SEV (5.29; 95% UI, 1.98 to 14.82) in 204 countries and territories, followed by Indonesia (6.32; 95% UI, 2.84 to 16.77) and Timor-Leste (7.19, 95% UI, 3.13 to 18.72) (Supplementary Table S3, Figure 1A).

**Table 1:** Global and regional age-standardized SEVs of diet high in processed meat for both sexes combined in 1990, 2000, 2010, and 2019 and EAPC of SEVs from 1990 to 2010 and 1990 to 2019.

	SEV 1990	SEV 2000	SEV 2010	SEV 2019	EAPC 1990–2010	EAPC 1990–2019
Global gender	30.95 (20.8 to 42.39)	30.94 (21.07 to 42.51)	30.56 (20.13 to 43.05)	29.81 (19.04 to 43.32)	−0.06 (−0.07 to −0.04)	−0.13 (−0.15 to −0.11)
Male	28.96 (18.97 to 40.59)	29.14 (19.34 to 40.89)	28.22 (17.66 to 41.80)	28.91 (18.50 to 41.82)	0 (−0.02 to 0.02)	−0.09 (−0.11 to −0.06)
Female SDI	32.78 (22.52 to 43.99)	32.62 (22.65 to 43.93)	31.35 (20.62 to 44.97)	32.12 (21.74 to 44.62)	−0.1 (−0.11 to −0.09)	−0.16 (−0.18 to −0.14)
High SDI	69.58 (47.25 to 88.24)	73.97 (54.8 to 89.78)	74.36 (56.09 to 89.67)	72.29 (53.82 to 87.89)	0.34 (0.27 to 0.41)	0.11 (0.04 to 0.18)
High-middle SDI	39.01 (29.14 to 50.76)	37.29 (26.45 to 49.79)	36.49 (23.48 to 51.5)	35.4 (21.91 to 51.89)	−0.32 (−0.35 to −0.29)	−0.31 (−0.33 to −0.29)
Middle SDI	10.96 (5.71 to 21.35)	12.59 (6.53 to 23.53)	14.47 (7.37 to 27.29)	16.3 (8.36 to 30.34)	1.43 (1.42 to 1.44)	1.39 (1.38 to 1.4)
Low-middle SDI	14.29 (7.72 to 26.38)	15.49 (8.47 to 27.8)	16.92 (9.3 to 29.29)	18.34 (10.18 to 31.77)	0.86 (0.85 to 0.87)	0.87 (0.87 to 0.88)
Low SDI region	21.13 (11.2 to 36.99)	22.09 (12.01 to 38.21)	22.76 (12.47 to 39.13)	23.36 (12.73 to 39.75)	0.39 (0.37 to 0.41)	0.34 (0.32 to 0.35)
Andean Latin America	9.65 (4.88 to 19.79)	10.35 (5.08 to 19.9)	11.11 (5.1 to 22.27)	11.98 (5.5 to 23.83)	0.72 (0.72 to 0.73)	0.73 (0.72 to 0.74)
Australasia	67.11 (42.08 to 90.06)	71.51 (46.38 to 93.54)	73.69 (47.17 to 96.43)	74.26 (48.27 to 96.59)	0.47 (0.43 to 0.51)	0.34 (0.29 to 0.38)
Caribbean	15.01 (6.6 to 31.9)	15.73 (7.1 to 33.23)	16 (7.25 to 33.95)	16.08 (7.25 to 33.97)	0.32 (0.28 to 0.35)	0.22 (0.19 to 0.25)
Central Asia	47.63 (28.04 to 69.19)	47.02 (27.53 to 67.93)	47.35 (28.32 to 68.71)	49.41 (29.81 to 70.64)	−0.02 (−0.04 to 0.01)	0.1 (0.06 to 0.15)
Central Europe	43.23 (21.51 to 66.8)	49.47 (26.88 to 72.31)	53.15 (28.6 to 76.69)	54.89 (30.27 to 78.26)	1.06 (0.98 to 1.14)	0.8 (0.72 to 0.88)
Central Latin America	18.66 (9.21 to 35.52)	19.8 (9.94 to 36.76)	20.67 (10.07 to 38.08)	21.47 (10.85 to 39.13)	0.52 (0.5 to 0.54)	0.47 (0.46 to 0.49)
Central Sub-Saharan Africa	16.53 (3.44 to 43.23)	15.61 (3.23 to 42.6)	15.37 (3.18 to 42.71)	15.74 (3.34 to 43.49)	−0.37 (−0.42 to −0.31)	−0.17 (−0.23 to −0.11)
East Asia	8.81 (4.07 to 19.17)	10.87 (5.01 to 22.06)	13.55 (5.94 to 28.64)	16.61 (7.44 to 34.03)	2.2 (2.19 to 2.22)	2.22 (2.21 to 2.23)
Eastern Europe	78.19 (70.15 to 86.44)	75.25 (62.58 to 85.53)	70.27 (49.88 to 87.93)	63.43 (40.66 to 85.03)	−0.54 (−0.57 to −0.5)	−0.7 (−0.75 to −0.65)
Eastern Sub-Saharan Africa	18.67 (8.77 to 36.62)	18.99 (8.9 to 37.37)	19.34 (9.06 to 38.1)	19.84 (9.36 to 38.33)	0.18 (0.18 to 0.19)	0.2 (0.19 to 0.21)
High-income Asia Pacific	59.89 (35.56 to 82.39)	68.52 (45.78 to 88.66)	70.33 (46.14 to 91.27)	64.87 (40.79 to 87.01)	0.78 (0.65 to 0.91)	0.28 (0.12 to 0.44)
High-income North America	76.38 (53.07 to 95.64)	82.32 (63.91 to 96.89)	84.57 (67.94 to 97.98)	83.72 (65.79 to 97.62)	0.51 (0.45 to 0.57)	0.31 (0.24 to 0.37)
North Africa and Middle East	11.18 (4.49 to 25.33)	12.14 (5.07 to 27.15)	12.93 (5.31 to 28.53)	13.44 (5.49 to 30.07)	0.73 (0.71 to 0.76)	0.64 (0.61 to 0.67)
Oceania	9.68 (4.11 to 20.53)	9.84 (4.21 to 20.36)	9.87 (4.26 to 20.52)	9.98 (4.33 to 20.98)	0.1 (0.08 to 0.11)	0.08 (0.07 to 0.09)
South Asia	13.77 (7.45 to 26.94)	15.18 (8.22 to 29.13)	16.27 (8.75 to 31.24)	17.09 (9.24 to 32.1)	0.85 (0.81 to 0.88)	0.74 (0.7 to 0.77)
Southeast Asia	7.03 (3.44 to 16.87)	7.67 (3.65 to 18.08)	8.45 (4.01 to 20.12)	9.57 (4.57 to 21.41)	0.94 (0.93 to 0.95)	1.04 (1.01 to 1.08)
Southern Latin America	46.67 (28.41 to 67.77)	50.74 (32.82 to 71.8)	54.35 (34.77 to 75.71)	59.43 (38.19 to 81.78)	0.77 (0.76 to 0.79)	0.79 (0.77 to 0.81)
Southern Sub-Saharan Africa	15.88 (6.97 to 33.08)	17.03 (7.79 to 34.28)	18 (8.28 to 36.3)	18.8 (8.7 to 37.12)	0.64 (0.62 to 0.66)	0.58 (0.56 to 0.6)
Tropical Latin America	16.67 (7.55 to 34.56)	18.87 (9.14 to 36.67)	21.5 (10.54 to 40.78)	24.43 (12.01 to 44.31)	1.29 (1.28 to 1.3)	1.32 (1.31 to 1.34)
Western Europe	74.68 (51.69 to 93.9)	77.63 (55.8 to 95.58)	78.62 (56.46 to 96.76)	78.23 (56.08 to 96.39)	0.25 (0.22 to 0.28)	0.15 (0.12 to 0.18)
Western Sub-Saharan Africa	30.72 (15.95 to 50.76)	33.58 (17.95 to 54.25)	35.13 (19.57 to 56.11)	35.69 (20.04 to 57.22)	0.67 (0.62 to 0.73)	0.5 (0.45 to 0.56)

SEV, summary exposure value; EAPC, estimated annual percentage change.

Further analysis on EAPC of age-standard SEV of DHIPM in two time-intervals, the first two decades, 1990–2010, and the full duration of the study, 1990–2019, were performed to explore the trends of the risk exposures of DHIPM. At the global level, the EAPC of SEVs for both sexes, males and females, were −0.06 (95% CI, −0.07 to −0.04), 0 (95% CI, −0.02 to 0.02), and −0.1 (95% CI, −0.11 to −0.09), respectively, from 1990 to 2010, and the EAPC of SEVs for both sexes, males and females, were −0.13 (95% CI, −0.15 to −0.11), −0.09 (95% CI, −0.11 to −0.06), and −0.16 (95% CI, −0.18 to −0.14), respectively, from 1990 to 2019. Compared with EAPC of SEV of DHIPM from 1990 to 2010, an obvious decrease was observed in that from 1990 to 2019 (Table 1). However, although an apparent decrease was observed in the age-standard SEV of DHIPM in the past three decades, it was noteworthy that only Eastern Europe (−0.7; 95% CI, −0.75 to −0.65) and Central sub-Saharan Africa (−0.17; 95% CI, −0.23 to −0.11) were facing a decreased age-standard SEV of DHIPM, while the age-standard SEV increased in other 19 regions worldwide from 1990 to 2019. Among them, the greatest increase was observed in East Asia (2.22; 95% CI, 2.21 to 2.23), followed by Tropical Latin America (1.32; 95% CI, 1.31 to 1.34) and Southeast Asia (1.04; 95% CI, 1.01 to 1.08). There exists a little difference between the trends within the past decade and within the past 30 years at the regional level. It is noteworthy that 7 regions had higher EAPCs of DHIPM while 14 regions had lower EAPCs from 1990 to 2019 compared with that from 1990 to 2010 (Table 1). At the national level, 22 countries were facing an obvious decrease in age-standard SEV of DHIPM from 1990 to 2019; among them, the lowest EAPC was observed in Russian Federation (−1.04; 95% CI, −1.11 to −0.98), followed by United Arab Emirates (−0.98; 95% CI, −0.99 to −0.97) and Democratic Republic of the Congo (−0.41; 95% CI, −0.52 to −0.31). Moreover, the top three countries in terms of growth in age-standard SEV of DHIPM were China (2.29; 95% CI, 2.28 to 2.3), Romania (2.25; 95% CI, 1.96 to 2.54), and Singapore (1.44; 95% CI, 1.43 to 1.45) (Supplementary Table S3, Figure 1B).

### Risk-attributable burden of DHIPM

**Figure 2:**
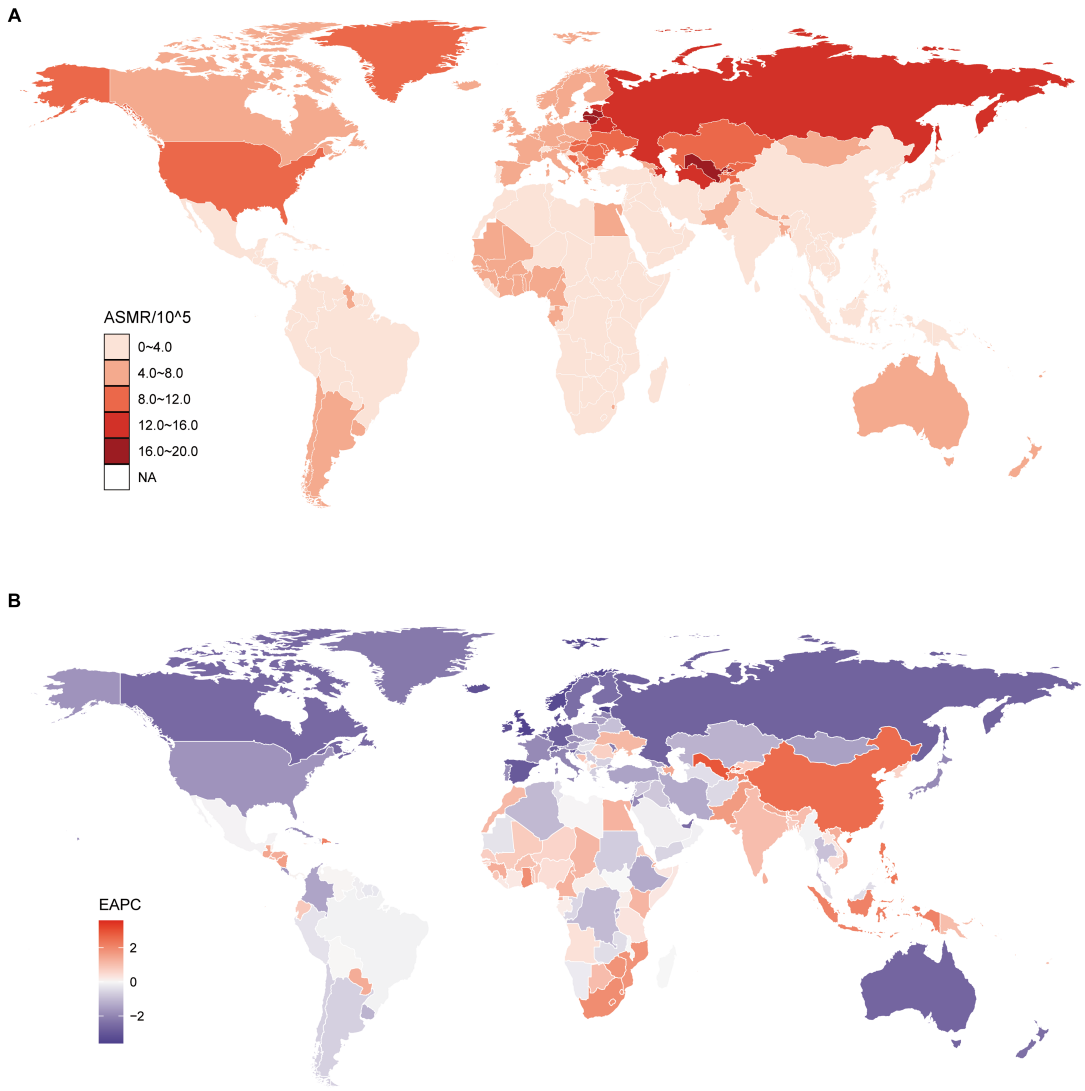
Global age standardized mortality rate to diet high in processed meat. **(A)** The all-cause ASMR per 100,000 associated with diet high in processed meat, for both sexes in 204 countries and territories in 2019. **(B)** The EAPC of ASMR of diet high in processed meat, for both sexes from 1990 to 2019, in 204 countries and territories. ASMR, age standardized mortality rate; EAPC, estimated annual percentage change.

Globally, in 2019, there were 304284.43 (95% UI, 154310.08 to 486449.43) deaths due to DHIPM, and an increase of 34.63% was observed compared with that in 1990. In 2019, the age-ASMR due to DHIPM was 3.9 (95% UI, 1.96 to 6.25) per 100,000 population, which had decreased in 1990–2010 (−1.84; 95% CI, −2.05 to −1.63) and 1990–2019 (−2.08; 95% CI, −2.2 to −1.95). At the regional level, the highest ASMR of DHIPM was observed in Eastern Europe (13.54; 95% UI, 3.18 to 25.2), followed by Central Asia (11; 95% UI, 3.46 to 23.48) and high-income North America (8.35; 95% UI, 3.52 to 12.77), while Andean Latin America had the lowest ASMR of DHIPM (0.93; 95% UI, 0.54 to 1.44) in 2019. As for the trends of DHIPM from 1990 to 2019, East Asia had the highest increase in the ASMR of DHIPM in 1990–2010 (2.55; 95% CI, 2.11 to 2.99) and 1990–2019 (2.39; 95% CI, 2.13 to 2.64). On the contrary, Western Europe had the greatest decrease in the ASMR of DHIPM (−2.76; 95% CI, −2.86 to −2.65) in 1990–2019 while the greatest decrease in the ASMR of DHIPM in 1990–2010 was observed in Australasia (−2.92; 95% CI, −3.04 to −2.8) (Supplementary Table S1). A considerable global difference of more than 10-fold was observed in ASMR for DHIPM between countries in 2019; the highest ASMR was observed in Lithuania (19.02; 95% UI, 4.46 to 34.09) while the lowest was observed in Peru with an ASMR of (0.55; 95% UI, 0.3 to 0.9) (Supplementary Table S4, Figure 2A). It was noteworthy that almost all countries in Europe, America, and Australia were facing a decreased ASMR from 1990 to 2019 while the ASMR in most countries in Asia and Africa had increased in the past 30 years (Figure 2B). Among these countries, Lesotho had the utmost increase in ASMR of DHIPM in both 1990–2010 (4.59; 95% CI, 4.12 to 5.07) and 1990–2019 (3.6; 95% CI, 3.21 to 4); on the contrary, Bermuda had the greatest decrease in ASMR (−3.98; 95% CI, −4.2 to −3.76) from 1990–2010 while Estonia had the greatest decrease (−3.6; 95% CI, −3.95 to −3.25) from 1990 to 2019 (Supplementary Table S4, Figure 2B).

**Figure 3:**
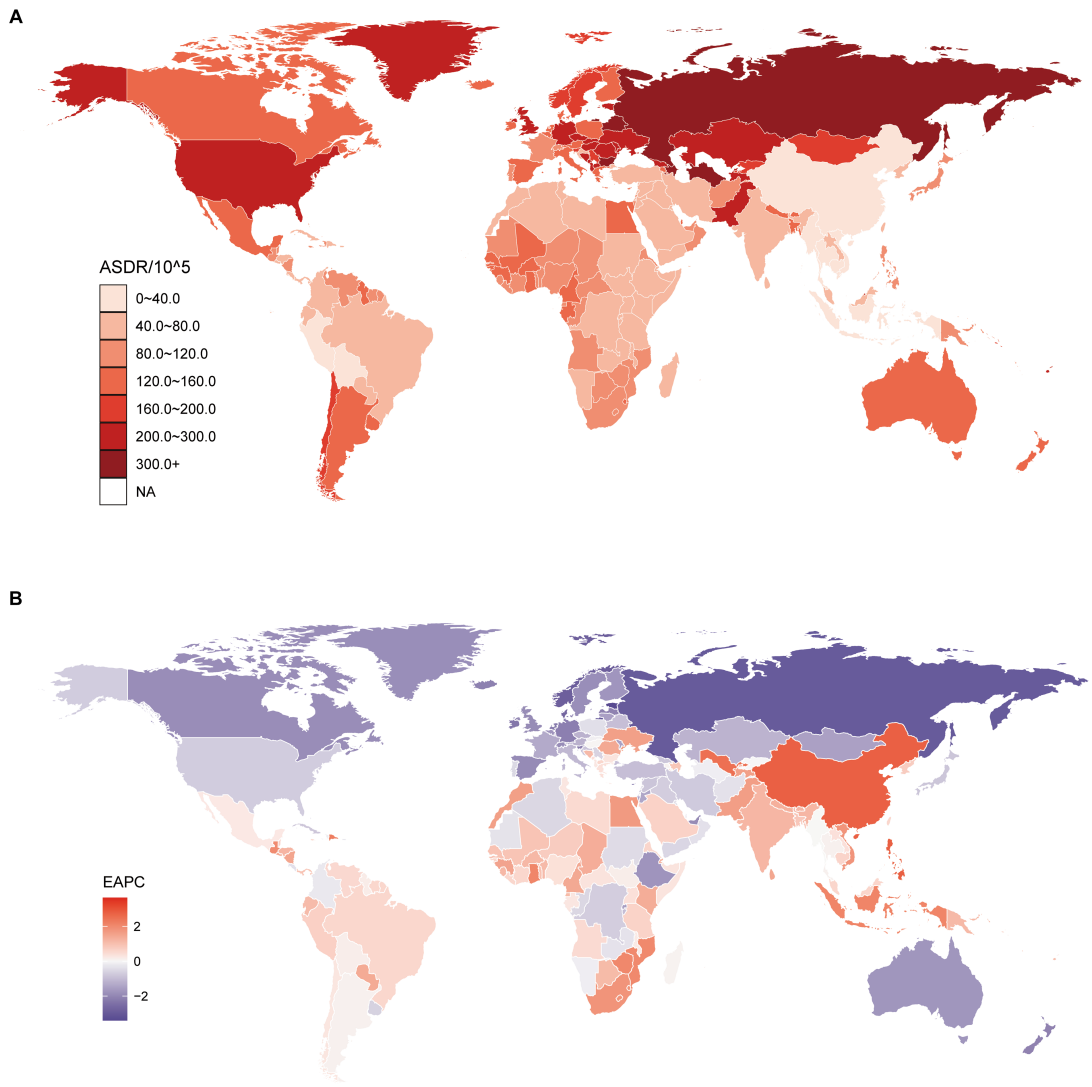
Global age standardized DALYs rate of diet high in processed meat. **(A)** The all-cause ASDR per 100,000 associated with diet high in processed meat, for both sexes in 204 countries and territories in 2019. **(B)** The EAPC of ASDR of diet high in processed meat, for both sexes from 1990 to 2019, in 204 countries and territories. DALYs, disease adjusted life year. ASDR, age standardized DALYs rate; EAPC, estimated annual percentage change.

There were 8556880.57 (95%UI, 5293182.27 to 12676193.19) DALYs due to DHIPM in 2019, which was increased by 68.69% when compared with that in 1990. The age-standardized DALY rate (ASDR) due to DHIPM was 104.35 (95%UI, 64.34 to 154.35) per 100,000 population in 2019, which had decreased in both 1990–2010 (EAPC, −1.2; 95% CI, −1.37 to −1.02) and 1990–2019 (EAPC, −1.36; 95% CI, −1.46 to −1.26). At the regional level, the highest ASDR of DHIPM in 2019 was observed in Eastern Europe (307.4; 95% UI, 97.14 to 541.15), followed by Central Asia (287.72; 95% UI, 123.08 to 540.71) and high-income North America (261.73; 95% UI, 150.78 to 367.58), while Andean Latin America had the lowest ASDR (30.21; 95% UI, 16.07 to 45.87). Similar with ASMR, the greatest increase in ASDR from 1990 to 2019 was observed in East Asia (2.65; 95% CI, 2.39 to 2.91), and Western Europe had the utmost decreased ASDR (−1.81; 95% CI, −1.91 to −1.72) from 1990 to 2019 (Supplementary Table S2). As shown in Figure 3A and Supplementary Table S5, the highest ASDR of DHIPM at the country level was observed in Uzbekistan (423.99; 95% UI, 159.66 to 854.42), followed by Belarus (355.78; 95% UI, 88.36 to 652.68) and Turkmenistan (336.54; 95% UI, 120.83 to 635.85), while the lowest ASDR of DHIPM was observed in Vietnam (16.56; 95% UI, 12.83 to 21.35). The extreme increase in ASDR of DHIPM was observed in Lesotho in both 1990–2010 (4.65; 95% CI, 4.21 to 5.09) and 1990–2019 (3.64; 95% CI, 3.25 to 4.03), while the greatest decrease in 1990–2010 was observed in Maldives (−3.38; 95% CI, −3.62 to −3.13) and Estonia (−3.4; 95% CI, −3.76 to −3.04) in 1990–2019 (Figure 3B, Supplementary Table S5).

### Causes of DHIPM-related mortality and disability

As stated above, every risk factor in GBD 2019 was associated with an outcome or outcomes, which was called risk-outcome pairs. According to GBD 2019, there were three causes for DHIPM-related mortality and disability (Supplementary Figure S1). IHD was the most important cause associated with both ASMR and ASDR of DHIPM in all years from 1990 to 2019, followed by diabetes mellitus and colon and rectum cancer, although the ASMR of DHIPM due to IHD had decreased from 5 to 2.5 per 100,000 population from 1990 to 2019, and the ASDR of DHIPM due to IHD had decreased from 100 to 50 per 100,000 population from 1990 to 2019. Moreover, although the ASMR of DHIPM due to diabetes mellitus and colon and rectum cancer was stable in a low burden, the ASDR of DHIPM due to diabetes mellitus had increased gradually in the past 30 years (Supplementary Figure S1B).

### Correlation of SEV and attributable burden of DHIPM with SDI

**Figure 4:**
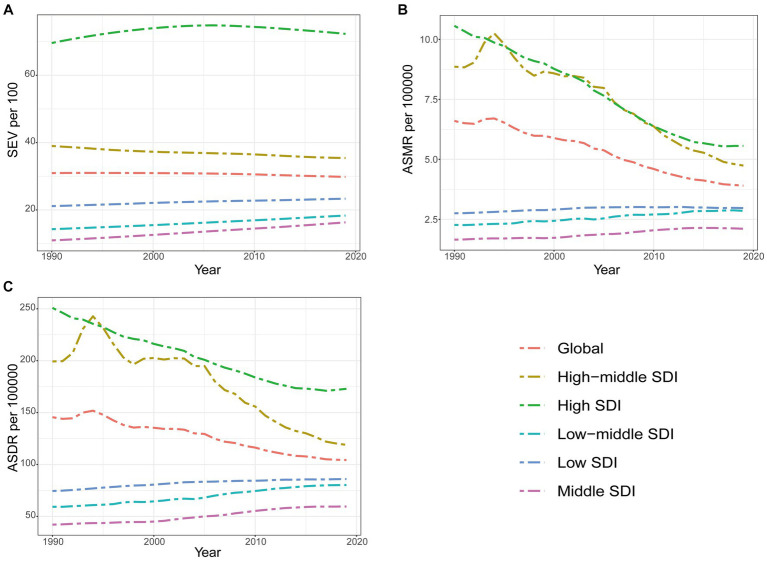
The exposure and burden of diet high in processed meat by SDI. **(A)** The age standardized SEV, **(B)** ASMR, and **(C)** ASDR of diet high in processed meat in different SDI regions from 1990 to 2019. Results are showed for both sexes worldwide. SEV, summary exposure value; ASMR, age standardized mortality rate; DALYs, disease adjusted life year; ASDR, age standardized DALYs rate.

Figure 4 demonstrates age-standardized SEV, mortality, and DALYs rate from 1990 to 2019 in global and five GBD regions. Compared with regions with lower SDI values, high-SDI regions were facing an apparently higher prevalence of DHIPM in all years from 1990 to 2019, and the age-standard SEVs in high SDI, middle SDI, low-middle SDI, and low SDI regions had increased gradually in this period while the age-standard SEVs in high-middle SDI regions had decreased (Figure 4A). Although the prevalence of DHIPM in high SDI regions remained severe in the past 30 years, the ASMR and ASDR of DHIPM in high SDI regions had decreased significantly in this period. In addition, the ASMR and ASDR of DHIPM in high-middle SDI regions had increased in the first 5 years, then decreased to and fluctuated around the original value from 1995 to 2005, and then decreased rapidly from 2005 to 2019 (Figures 4B,C). The trends of ASMR and ASDR of DHIPM in middle SDI, low-middle SDI, and low SDI regions showed the same pattern with the change in age-standardized SEV in these regions from 1990 to 2019 (Figures 4B,C).

**Figure 5:**
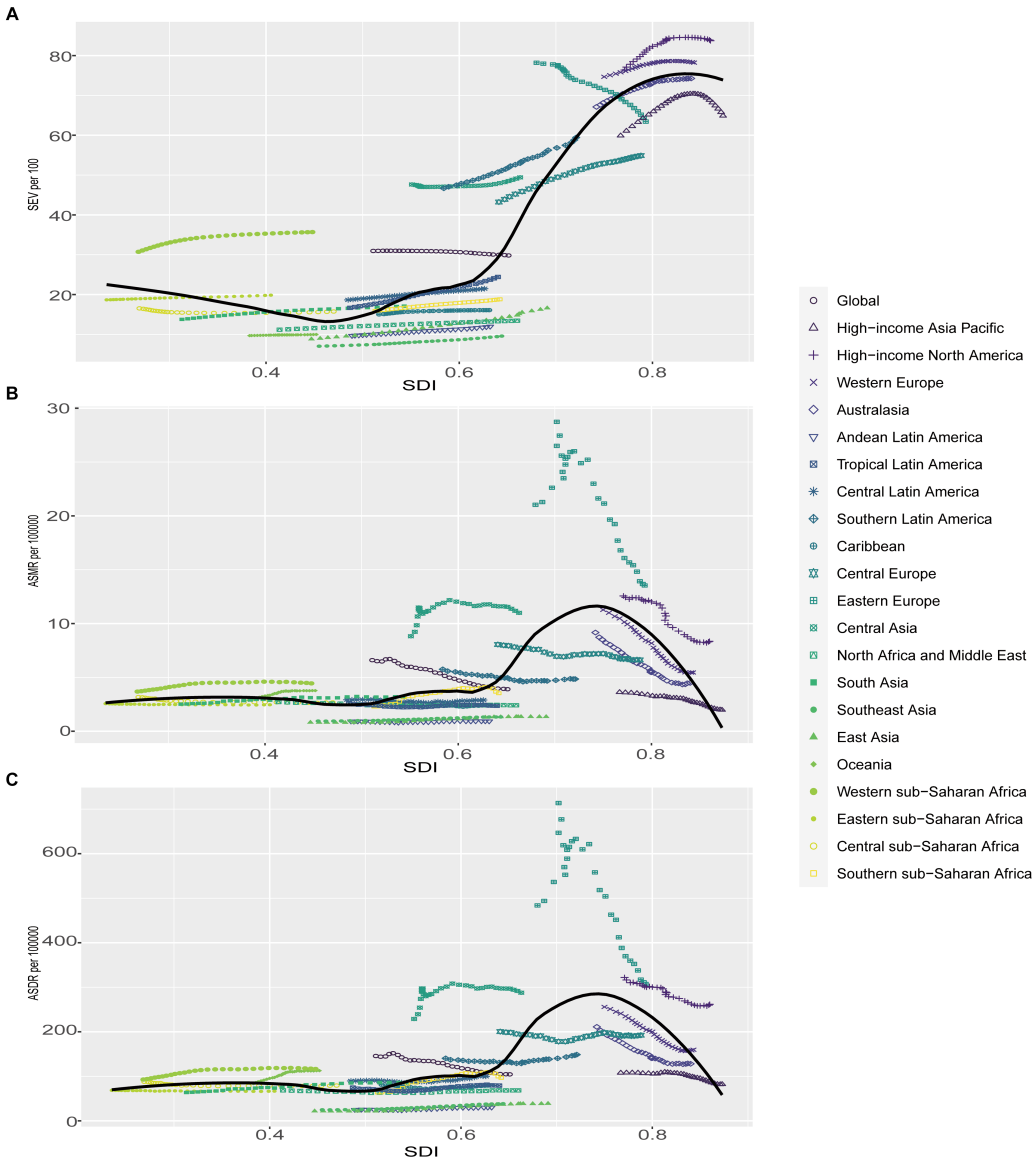
Correlations of SEV, ASMR as well as ASDR and SDI at the regional level. Age-standardized SEV **(A)**, ASMR **(B)** as well as ASDR **(C)** for diet high in processed meat and SDI at the regional level in 21 regions from 1990 to 2019. SEV, summary exposure value; ASMR, age standardized mortality rate; DALYs, disease adjusted life year; ASDR, age standardized DALYs rate.

As for the correlation with SDI, an obvious positive association was observed in age-standardized SEV of DHIPM with SDI (Figure 5A, Supplementary Figure S2A). The association between ASMR of DHIPM and SDI was not significant when SDI value was less than 0.6, and then, a positive association was observed when SDI ranges from 0.6 to 0.75 while the association turned into negative when SDI was more than 0.75 (Figure 5B, Supplementary Figure S2B); the same pattern was also observed in the correlation between ASDR of DHIPM and SDI (Figure 5C, Supplementary Figure S2C). At the regional level, high-income North America, Western Europe, Central Asia, Southern Latin America, and Western sub-Saharan Africa demonstrated higher observed SEVs of DHIPM compared with the expected trends based on SDI over the past 30 years; on the contrary, high-income North Asia Pacific, North Africa and Middle East, Oceania, East Asia, Caribbean, Southeast Asia, and Andean Latin America exhibited lower observed SEVs than expected (Figure 5A). The trends of ASMR and ASDR at regional level were similar, among the 21 regions worldwide, the observed ASMR and ASDR in Eastern Europe, Central Asia, High-income North America and Western Sub-Saharan Africa were higher than expected values on the basis of SDI over the period while Australasia, High-income North Asia Pacific, Andean Latin America, East Asia and Southeast Asia had lower observed ASMR and ASDR (Figures 5B,C). Compared with the expected values according to their SDI values at the national level, the trends of observed SEVs, ASMR, and ASDR were similar to that at the regional level. Among the 204 countries and territories, Albania demonstrated the biggest separation between the observed SEV value and the expected value based on SDI while Uzbekistan had the greatest difference in the comparison between observed values and expected values for both ASMR and ASDR of DHIPM (Supplementary Figure S2).

### Age and sex patterns

**Figure 6:**
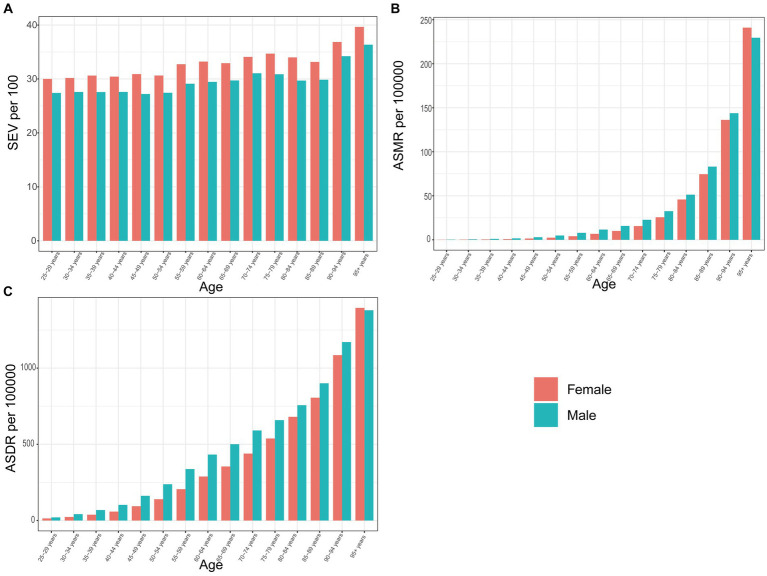
The exposure and burden of diet high in processed meat by age and sex. The all-cause **(A)** age standardized SEV, **(B)** ASMR, and **(C)** ASDR of diet high in processed meat worldwide in different age groups. SEV, summary exposure value; ASMR, age standardized mortality rate; DALYs, disease adjusted life year; ASDR, age standardized DALYs rate.

Compared with women, men were facing a lower age-standardized SEV; however, higher mortality and DALYs rate of DHIPM in all years from 1990 to 2019 and the separation between genders demonstrated an overall stable trend in the past three decades (Supplementary Figure S3). Although women had a 14.28% higher age-standardized SEV of DHIPM when compared with men, the ASMR in women was approximately 25% lower than men and the ASDR was 33.33% lower in women compared with men (Supplementary Figure S3). In GBD 2019, only the data on the age distribution in age-standardized SEV, AMSR, and ASDR of DHIPM in those aged 25 years and older were available. Generally, women were facing a higher age-standardized SEV in all age groups and lower ASMR and ASDR in most age groups, except for those aged 95 + years (Figure 6). In general, the elderly were facing comparable age-standardized SEV however severer burden of DHIPM compared with youngers, moreover the separation of ASMR enlarged rapidly after 70 years old while the difference of ASDR between elderly and youngers increased in a stable speed with age in 2019 (Figure 6). The ratio of male to female SEV was fluctuated approximately 0.90 in all age groups in 2019, in which the highest ratio was observed in aged 90–94 years old group which was the lowest observed in 80 to 84 years old group (Supplementary Figure S4A). Moreover, although the male to female ratios in ASMR and ASDR of DHIPM in all age groups were always higher than 1.0 in 2019, the gap between genders only increased in those aged 25–34 years old, decreasing in those older than 34 years old with age (Supplementary Figures S4B,C).

## Discussion

Compared with diet high in red meat, the harm caused by diet high in processed meat was acute to humans due to the production of toxic substances (such as heterocyclic amines, polycyclic aromatic hydrocarbons and so on) in the progress of cooking at high temperatures (15, 21). In this study, we systematically analyzed and demonstrated the prevalence and attributable burden of DHIPM and its correlation with SDI, year, and age group. Globally, the prevalence and attributable burden of DHIPM had decreased for both sexes, males and females, from 1990 to 2019, while the trends of ASMR had decreased with a faster speed compared with age-standard SEV and ASDR; in addition, women had greater decrease in the trends of age-standard SEV and ASMR while the decrease in the trend of ASDR was observed in men from 1990 to 2019. At the regional level, regions with higher SDI values such as high-income North America, Western Europe, Australasia, and Eastern Europe had higher prevalence compared with other regions while the EAPC values of age-standard SEV, ASMR, and ASDR in these regions, which indicated the alteration in the prevalence and attributable burden of DHIPM in a certain period, were universally lower than those in regions with lower SDI values; moreover, the same phenomenon could also be observed at the nation level in the current study. We also found a generally positive correlation between the age-standard SEV, ASMR, and ASDR of DHIPM and SDI, indicating that high SDI and high-middle SDI regions were facing higher prevalence and attributable burden of DHIPM than middle SDI, low-middle SDI, and low SDI regions, which was consistent with our results. According to our study, men were facing a lower age-standard SEV and higher ASMR and ASDR in all age groups from 1990 to 2019 compared with women.

Although public health concerns of processed meat consumption had been growing (20, 30), our study demonstrated the increased prevalence of DHIPM in most regions and countries; however, the age-standard SEV was found decreased, which might be explained by the high-speed growth of population from 1990 to 2019 worldwide (26). Moreover, the clear shift in dietary patterns toward high energy-dense diet over the past decades, which was characterized by higher consumption of food and animal origin, might also contribute to the increased prevalence of DHIPM (15). At the regional level, positive correlations were observed between SDI and the prevalence and attributable burden of DHIPM, indicating the dilemma of DHIPM in regions with higher SDI, especially high SDI region. However, the EAPC values in these regions were relatively lower compared with undeveloped regions, revealing the general decrease in the prevalence and attributable burden of DHIPM in regions with higher SDI. This phenomenon might largely due to the growing awareness of the negative health effects of processed meat, sufficient healthier food substitute (including white meat, poultry, and plant-based alternatives), and greater compliance with the dietary recommendations in people in regions with higher SDI (31–34). Moreover, sufficient medical resource and pleasant economic condition allowed people in high-income regions to receive effective prevention and treatment of diseases associated with excessive processed meat consumption (especially IHD), thus reducing the ASMR and ASDR attributable to DHIPM (33–36). As for developing regions, although lower age-standard SEV, ASMR and ASDR were demonstrated in our study when compared with high-income regions, the ever-fast increasing speeds of prevalence and attributable burden of DHIPM in these regions could not be ignored, because of that overconsumption of processed meat would lead to severer burden in these regions without adequate health policy reformation while the economic development was persistent in these regions in the future. Sievert et al. (37) proposed that the increased consumption of processed meat and the reduced improvement in treatment and emergency services together contributed to the increasing trend in disease burden in low-income and middle-income countries over the past decades. Thus, highly efficient healthcare systems were in urgent need in countries in the middle or low-middle SDI regions, and stakeholders in these regions should always keep this dietary risk factor in mind together with the algorithm to develop the economics. The findings at the national level were comparable to the regional level.

GBD 2019 defined IHD, diabetes mellitus, and colon and rectum cancer as outcomes of DHIPM, and IHD was the most common outcome of the ASMR and ASDR of DHIPM, although a previous research demonstrated that diabetes mellitus was found serving as the most important outcome (21). Moreover, the attributable burden of DHIPM associated with IHD was consistently decreasing from 1990 to 2019; however, the ASDR associated with diabetes mellitus was increasing over the past three decades. The changing pattern of attributable burden of DHIPM in the past three decades indicated the well-controlled disease burden of IHD in most countries (38). However, the prevalence of diabetes mellitus had increased greatly [529 million (95% UI, 500 to 564) people living with diabetes mellitus worldwide] in the past 30 years due to the rapid economic development, urbanization, and growth in population (39). As a chronic disease associated with obesity and a sedentary lifestyle (40), early diagnosis, patient education, and regular visits to clinicians provided patients with possibility of preventing the onset of diabetes mellitus and prolonging their survival duration, which might explain why the ASDR of DHIPM associated with diabetes mellitus had increased over the past three decades (41). Despite lower exposure level of DHIPM, men possessed higher ASMR and ASDR compared with women, as evidenced by a 37.5% higher mortality rate and a 43.5% higher DALYs rate, and the gap between genders kept in a stable narrow over the past three decades, according to a previous research (21). The separation of prevalence and attributable burden of DHIPM between genders in different age groups were similar to that in all ages, except for the trend in those aged 95 + years. Generally, the age-standard SEV and ASDR increased with a stable speed in the progress of aging however the growing speed of ASMR increased rapidly after 85 years old, the reason why this phenomenon occurred might be that as a dietary risk, processed meat influenced humans gradually and the outcomes of DHIPM, such as IHD and diabetes mellitus, were all chronic diseases, which would not acutely affect the longevity of patients once well controlled.

In the progress of continuous urbanization, rapid increases in the trade of global processed meat allowed growth in processed meat consumption over the decades, thus changing the dietary and exacerbating the spread of diet-related non-communicable diseases worldwide (42, 43). Considering an avoidable risk factor, DHIPM, which mean that more energy and chemical additives and less micronutrient content were ingested, was associated with decreased quality of the diet and increased risk of all causes of premature deaths. Since the reduction of process meat consumption was possible and desirable to prevent deaths in populations with high risk as well as the prevalence and heavy burden of DHIPM globally, guideline and education on diet pattern were in urgent need by all countries in the world, especially for developing countries because of that the government and policy-makers seldom paid attention to this issue. Moreover, previous articles had indicated that decrease in processed meat consumption may have potential benefits on reducing the use of environmental resources and alleviating the risk of premature deaths associated with dietary risk factors, therefore a healthy food consumption pattern was not only regarded as beneficial for human bodies but also helpful to maintain environmental balance (44).

To the best of our knowledge, this was the most updated study to demonstrate the epidemiology of DHIPM globally, which included 204 countries and some that even had not been involved before. It was worth noting that essential measures were in urgent need to alleviate the burden of DHIPM due to its acute prevalence as well as attributable burden and the potential damage on humans as well as environment of processed meat, which contained less proteins, essential amino acids, minerals (such as iron, potassium and zinc) however more carcinogen, including saturated fat, heterocyclic amines and polycyclic aromatic hydrocarbons. This study also had some limitations which were common for all GBD estimates (25, 26). First of all, the availability and completeness data source were incomplete. In addition, some regions were lacked in the source data, and trends in these regions were predicted by trends from neighboring locations, leading to discrepant accuracy of estimates among different countries. Finally, GBD 2019 did not include the results of some current national food surveys, such as Family Budget Survey.

## Conclusion

The past three decades had observed a slight decrease in DHIPM globally; however, high-income regions were facing severe prevalence and attributable burden while developing regions were suffering from greater increase in the prevalence and attributable burden from 1990 to 2019. IHD remained the most common outcome of DHIPM from 1990 to 2019, although the ASMR and ASDR associated with IHD had decreased year by year. Compared with females, males were observed with lower level of exposure however higher attributable burden of DHIPM. Education on healthy diet pattern and cessation on ultra-processed food were in urgent need, especially in developing countries, and the maintenance of dietary health could be a reasonable strategy to alleviate the risk of premature deaths and maintain environmental balance.

## Data availability statement

The original contributions presented in the study are included in the article/Supplementary material, further inquiries can be directed to the corresponding author.

## Author contributions

F-XW: Writing – original draft, Writing – review & editing. X-HK: Conceptualization, Data curation, Methodology, Writing – original draft. ZG: Conceptualization, Supervision, Writing – review & editing. L-XL: Formal analysis, Validation, Writing – review & editing. SZ: Conceptualization, Data curation, Investigation, Methodology, Software, Supervision, Writing – original draft, Writing – review & editing.

## Footnotes

^1^ http://ghdx.healthdata.org/gbd-results-tool

## Supplementary material

### Abbreviations

ASMR, Age standardized mortality rate; DALYs, Disease adjusted life years; ASDR, Age standardized DALY rate; SDI, Sociodemographic index; EAPC, Estimated annual percentage change.
