# Erratum: Global, regional, and national burden of diet high in processed meat from 1990 to 2019: a systematic analysis from the global burden of disease study 2019

**DOI:** 10.3389/fnut.2025.1563555

**Published:** 2025-01-30

**Authors:** Frontiers Production Office

**Affiliations:** Frontiers Media SA, Lausanne, Switzerland

**Keywords:** diet high in processed meat, dietary pattern, disability-adjusted life year, GBD 2019, mortality, summary exposure value

Following publication, we found a non-genuine email address had been provided for reviewer Alaa El-Din Bekhit. Following an investigation, which was conducted in accordance with Frontiers policy, we confirmed that the real Alaa El-Din Bekhit was impersonated and did not take any action on this manuscript. They have therefore been removed from this article. In line with the COPE guidelines on potential peer review manipulation, we conducted a post-publication assessment that concluded that the article meets the standards for publication at Frontiers.

The original article has been updated.

