# Supplementary material for: Global, regional, and national burden of diet high in processed meat from 1990 to 2019: a systematic analysis from the global burden of disease study 2019
Source: Front Nutr. 2024 Feb 13;11:1354287. doi: 10.3389/fnut.2024.1354287 (PMC10896824; doi:10.3389/fnut.2024.1354287)
Supplement: Supplementary file 5 [file Table_1.docx]

Table 1S. Global and regional age-standardized mortality of diet high in processed meat for both sexes combined in 1990,2000,2010, and 2019, and EAPC of ASMR from 1990 to 2010 and 1990 to 2019

|  | ASMR 1990 | ASMR2000 | ASMR 2010 | ASMR 2019 | EAPC 1990-2010 | EAPC 1990-2019 |
| --- | --- | --- | --- | --- | --- | --- |
| Global  Gender | \| 6.6(2.34 to 10.97) \| \| --- \| | 5.89(2.32 to 9.49) | 4.6(2.12 to 7.35) | \| 3.9(1.96 to 6.25) \| \| --- \| | -1.84 (-2.05 to -1.63) | -2.08 (-2.2 to -1.95) |
| Male | 7.4(2.52 to 12.4) | 6.65(2.52 to 10.75) | 5.35(2.41 to 8.61) | 4.55(2.22 to 7.3) | -1.68 (-1.88 to -1.48) | -1.9 (-2.02 to -1.79) |
| Female  SDI | 5.81(2.18 to 9.54) | 5.11(2.11 to 8.14) | 3.9(1.86 to 6.18) | 3.31(1.74 to 5.24) | -2.01 (-2.22 to -1.8) | -2.22 (-2.34 to -2.09) |
| High SDI | 6.6(2.34 to 10.97) | 8.77(3.47 to 13.74) | 6.37(2.8 to 9.72) | 5.56(2.5 to 8.51) | -2.42 (-2.6 to -2.24) | -2.51 (-2.63 to -2.39) |
| High-middle SDI | 10.56(3.62 to 17.39) | 8.58(2.49 to 14.4) | 6.37(2.16 to 10.92) | 4.74(1.87 to 8.31) | -1.83 (-2.27 to -1.39) | -2.6 (-2.9 to -2.31) |
| Middle SDI | 8.86(2.53 to 15.07) | 1.73(1 to 2.96) | 2.04(1.17 to 3.52) | 2.11(1.2 to 3.58) | 1 (0.86 to 1.13) | 1.06 (0.97 to 1.15) |
| Low-middle SDI | 1.65(0.94 to 2.83) | 2.44(1.46 to 3.92) | 2.7(1.63 to 4.32) | 2.85(1.79 to 4.51) | 0.96 (0.89 to 1.04) | 0.93 (0.89 to 0.98) |
| Low SDI  Region | 2.27(1.34 to 3.75) | 2.91(1.52 to 5.16) | 3(1.59 to 5.25) | 2.97(1.57 to 5.24) | 0.51 (0.46 to 0.56) | 0.29 (0.23 to 0.36) |
| Andean Latin America | 0.92(0.62 to 1.4) | 0.84(0.56 to 1.22) | 0.93(0.58 to 1.37) | 0.93(0.54 to 1.44) | -0.23 (-0.5 to 0.04) | 0.2 (0.02 to 0.38) |
| Australasia | 9.17(3.15 to 16.8) | 6.48(2.7 to 11.33) | 5.05(2.42 to 8.13) | 4.49(2.19 to 7.04) | -2.92 (-3.04 to -2.8) | -2.64 (-2.79 to -2.5) |
| Caribbean | 2.89(1.29 to 5.49) | 2.46(1.12 to 4.53) | 2.33(1.07 to 4.25) | 2.37(1.06 to 4.34) | -1.12 (-1.29 to -0.94) | -0.7 (-0.86 to -0.54) |
| Central Asia | 8.82(2 to 20.09) | 11.08(2.7 to 25.59) | 11.68(3.21 to 25.42) | 11(3.46 to 23.48) | 1.07 (0.68 to 1.46) | 0.48 (0.23 to 0.73) |
| Central Europe | 8.05(2.44 to 17.39) | 6.95(2.23 to 14.88) | 7.09(2.6 to 13.45) | 6.62(2.56 to 12.12) | -0.65 (-0.85 to -0.44) | -0.62 (-0.72 to -0.52) |
| Central Latin America | 2.92(1.51 to 4.93) | 2.69(1.41 to 4.41) | 2.72(1.46 to 4.4) | 2.9(1.52 to 4.71) | -0.54 (-0.69 to -0.39) | -0.14 (-0.28 to 0) |
| Central Sub-Saharan Africa | 3.17(1.46 to 5.98) | 2.86(1.3 to 5.43) | 2.69(1.22 to 5.16) | 2.68(1.21 to 5.11) | -0.86 (-0.93 to -0.79) | -0.63 (-0.71 to -0.56) |
| East Asia | 0.82(0.49 to 1.41) | 0.87(0.51 to 1.52) | 1.29(0.62 to 2.56) | 1.35(0.58 to 2.76) | 2.55 (2.11 to 2.99) | 2.39 (2.13 to 2.64) |
| Eastern Europe | 21.03(4.23 to 36.71) | 25.32(4.89 to 43.91) | 19.24(3.98 to 34.22) | 13.54(3.18 to 25.2) | -0.65 (-1.38 to 0.09) | -2.17 (-2.7 to -1.63) |
| Eastern Sub-Saharan Africa | 2.56(1.22 to 4.42) | 2.49(1.2 to 4.33) | 2.46(1.22 to 4.32) | 2.46(1.24 to 4.36) | -0.19 (-0.22 to -0.16) | -0.14 (-0.17 to -0.12) |
| High-income Asia Pacific | 3.62(1.65 to 6.33) | 3.18(1.61 to 4.96) | 2.64(1.38 to 4.06) | 1.98(1.02 to 3.1) | -1.47 (-1.59 to -1.34) | -2.06 (-2.25 to -1.87) |
| High-income North America | 12.56(4.15 to 20.77) | 12.02(4.7 to 18.67) | 8.97(3.74 to 13.69) | 8.35(3.52 to 12.77) | -1.55 (-1.87 to -1.23) | -1.81 (-2 to -1.62) |
| North Africa and Middle East | 2.71(1.21 to 5.64) | 2.42(1.06 to 5.1) | 2.42(1.04 to 5.13) | 2.41(1.03 to 5.06) | -0.69 (-0.81 to -0.56) | -0.37 (-0.48 to -0.26) |
| Oceania | 2.87(1.49 to 4.65) | 3.5(1.75 to 5.48) | 3.72(1.86 to 5.87) | 3.77(1.85 to 5.97) | 1.32 (1.17 to 1.47) | 0.87 (0.73 to 1.02) |
| South Asia | 2.52(1.58 to 3.92) | 2.84(1.81 to 4.27) | 3.11(2.06 to 4.56) | 3.15(2.13 to 4.61) | 1.18 (1.06 to 1.3) | 0.89 (0.79 to 0.99) |
| Southeast Asia | 0.94(0.69 to 1.3) | 1.04(0.73 to 1.52) | 1.2(0.79 to 1.85) | 1.33(0.83 to 2.06) | 1.26 (1.19 to 1.33) | 1.35 (1.3 to 1.39) |
| Southern Latin America | 5.75(2.57 to 10.84) | 4.98(2.62 to 8.42) | 4.69(2.61 to 7.48) | 4.85(2.77 to 7.44) | -1.03 (-1.12 to -0.93) | -0.59 (-0.73 to -0.45) |
| Southern Sub-Saharan Africa | 2.29(1.13 to 3.8) | 3.39(1.68 to 5.49) | 4.01(2.06 to 6.19) | 3.53(1.82 to 5.35) | 3.05 (2.67 to 3.43) | 1.94 (1.55 to 2.33) |
| Tropical Latin America | 2.61(1.16 to 5.15) | 2.24(1.03 to 4.16) | 2.41(1.17 to 4.33) | 2.36(1.22 to 4.09) | -0.46 (-0.72 to -0.19) | -0.04 (-0.22 to 0.13) |
| Western Europe | 11.27(4.06 to 18.2) | 8.69(3.55 to 13.57) | 6.29(2.88 to 9.55) | 5.46(2.58 to 8.23) | -2.82 (-2.92 to -2.71) | -2.76 (-2.86 to -2.65) |
| Western Sub-Saharan Africa | 3.68(1.76 to 7.24) | 4.23(2.16 to 7.77) | 4.54(2.38 to 7.92) | 4.44(2.34 to 7.8) | 1.18 (1.06 to 1.3) | 0.75 (0.6 to 0.89) |

ASMR, age-standard morality rate; EAPC, estimated annual percentage change.
