# Supplementary material for: Global, regional, and national burden of diet high in processed meat from 1990 to 2019: a systematic analysis from the global burden of disease study 2019
Source: Front Nutr. 2024 Feb 13;11:1354287. doi: 10.3389/fnut.2024.1354287 (PMC10896824; doi:10.3389/fnut.2024.1354287)
Supplement: Supplementary file 6 [file Table_2.docx]

Table 2S. Global and regional age-standardized DALYs of diet high in processed meat for both sexes combined in 1990,2000,2010, and 2019, and EAPC of ASDR from 1990 to 2010 and 1990 to 2019

|  | ASDR 1990 | ASDR 2000 | ASDR 2010 | ASDR 2019 | EAPC 1990-2010 | EAPC 1990-2019 |
| --- | --- | --- | --- | --- | --- | --- |
| Global  Gender | \| 145.57(63.48 to 226.67) \| \| --- \| | 135.4(64.23 to 206.53) | 116.33(66.78 to 172.98) | \| 104.35(64.34 to 154.35) \| \| --- \| | -1.2 (-1.37 to -1.02) | -1.36 (-1.46 to -1.26) |
| Male | \| 172.74(68.95 to 274.29) \| \| --- \| | \| 162.33(70.74 to 252.72) \| \| --- \| | \| 139.8(75.99 to 211.6) \| \| --- \| | \| 123.73(73.6 to 186.91) \| \| --- \| | -1.15 (-1.35 to -0.94) | -1.37 (-1.48 to -1.25) |
| Female  SDI | 118.85(60.12 to 180.93) | 108.77(59.07 to 161.59) | 93.86(58.23 to 134.98) | 86.07(55.05 to 123.34) | -1.23 (-1.37 to -1.1) | -1.31 (-1.38 to -1.23) |
| High SDI | 250.85(108.03 to 390.78) | 216.2(109.6 to 315.42) | 183.86(107.84 to 259.13) | 172.94(106.31 to 241.41) | -1.48 (-1.53 to -1.43) | -1.41 (-1.47 to -1.35) |
| High-middle SDI | 199.28(69.97 to 324.45) | 202.59(71.46 to 326.89) | 155.88(68.49 to 249.14) | 118.93(61.47 to 188.81) | -1.45 (-1.95 to -0.95) | -2.29 (-2.62 to -1.96) |
| Middle SDI | 42.11(24.73 to 69.1) | 45.07(26.5 to 73.26) | 55.32(31.65 to 90.14) | 59.52(34.7 to 95.39) | 1.33 (1.18 to 1.49) | 1.43 (1.33 to 1.53) |
| Low-middle SDI | 59.32(36.46 to 93.71) | 64.5(39.67 to 99.98) | 74.5(46.66 to 114.8) | 80.29(50.76 to 121.34) | 1.16 (1.07 to 1.25) | 1.2 (1.15 to 1.25) |
| Low SDI  Region | 74.48(39.61 to 128.39) | 80.64(43.96 to 136.11) | 84.4(47.67 to 140.35) | 86.01(48.59 to 142.06) | 0.67 (0.62 to 0.73) | 0.5 (0.44 to 0.56) |
| Andean Latin America | 24.83(15.2 to 38.75) | 24.47(15.03 to 35.98) | 28.97(16.58 to 43.64) | 30.21(16.07 to 45.87) | 0.55 (0.29 to 0.8) | 0.82 (0.67 to 0.97) |
| Australasia | 211.01(84.24 to 359.07) | 159.48(83 to 249.4) | 134.92(78.12 to 193.44) | 128.05(77.25 to 179.45) | -2.23 (-2.36 to -2.1) | -1.77 (-1.93 to -1.61) |
| Caribbean | 81.11(36.61 to 143.39) | 74.13(34.67 to 124.61) | 74.21(36.15 to 122.54) | 79.28(37.66 to 132.75) | -0.45 (-0.59 to -0.32) | -0.08 (-0.21 to 0.06) |
| Central Asia | 228.77(70.86 to 457.44) | 282.81(91.11 to 593.5) | 298.18(112.56 to 594.43) | 287.72(123.08 to 540.71) | 0.97 (0.58 to 1.35) | 0.47 (0.24 to 0.7) |
| Central Europe | 200.37(78.62 to 386.41) | 178.1(75.11 to 333.77) | 196.99(97.69 to 329.15) | 192.38(102.09 to 305.62) | -0.1 (-0.36 to 0.16) | -0.01 (-0.14 to 0.12) |
| Central Latin America | 89.58(47.94 to 137.69) | 86.08(46.15 to 129.7) | 90.5(49.38 to 135.29) | 99.95(56.42 to 147.14) | -0.08 (-0.21 to 0.05) | 0.32 (0.19 to 0.46) |
| Central Sub-Saharan Africa | 87.68(42.25 to 157.11) | 81.24(39.28 to 145.17) | 78.34(37.53 to 139.66) | 79.21(38.46 to 138.87) | -0.61 (-0.67 to -0.54) | -0.37 (-0.44 to -0.29) |
| East Asia | 21.89(12.35 to 36.04) | 22.98(12.45 to 38.25) | 35.03(16.54 to 63.36) | 38.87(18.3 to 71.18) | 2.61 (2.13 to 3.09) | 2.65 (2.39 to 2.91) |
| Eastern Europe | 483.73(113.59 to 841.16) | 608.63(136.33 to 1057.88) | 451.41(124.06 to 764.55) | 307.4(97.14 to 541.15) | -0.6 (-1.47 to 0.27) | -2.35 (-2.97 to -1.73) |
| Eastern Sub-Saharan Africa | 68.42(33.48 to 113.5) | 67.38(33.97 to 111.73) | 66.64(33.92 to 111.07) | 66.62(34.43 to 110.87) | -0.13 (-0.16 to -0.1) | -0.11 (-0.13 to -0.09) |
| High-income Asia Pacific | 107.72(61.23 to 165.77) | 108.81(68.9 to 152.12) | 96.61(62.77 to 133.51) | 82.14(54.1 to 114.24) | -0.46 (-0.63 to -0.29) | -1.01 (-1.19 to -0.84) |
| High-income North America | 321.9(137.84 to 492.31) | 300.31(142.15 to 441.27) | 269.33(153.39 to 384.75) | 261.73(150.78 to 367.58) | -0.75 (-0.84 to -0.67) | -0.81 (-0.87 to -0.75) |
| North Africa and Middle East | 68.43(28.57 to 143.23) | 62.72(26.28 to 129.27) | 66.41(27.98 to 131.37) | 68.39(29.15 to 132.85) | -0.27 (-0.42 to -0.11) | 0.09 (-0.03 to 0.22) |
| Oceania | 83.52(40.66 to 136.46) | 102.21(48.35 to 162.36) | 110.51(53.05 to 174.99) | 112.65(52.03 to 180.76) | 1.45 (1.31 to 1.58) | 0.99 (0.84 to 1.13) |
| South Asia | 63.64(41.06 to 96.12) | 72.33(46.47 to 107.46) | 82.11(54.64 to 119.17) | 85.73(58.06 to 124.36) | 1.28 (1.19 to 1.38) | 1.15 (1.08 to 1.22) |
| Southeast Asia | 24.75(17.43 to 34.35) | 27.61(18.54 to 40.28) | 33.12(20.85 to 50.94) | 38.43(23.22 to 58.68) | 1.53 (1.42 to 1.63) | 1.73 (1.65 to 1.81) |
| Southern Latin America | 140.34(70.73 to 245.9) | 130.85(76.31 to 205.04) | 135.25(83.67 to 197.91) | 148.69(94.42 to 210.74) | -0.21 (-0.32 to -0.1) | 0.22 (0.08 to 0.36) |
| Southern Sub-Saharan Africa | 63.61(30.36 to 103.3) | 90.16(44.54 to 142.44) | 107.24(55.92 to 160.63) | 96.68(51.52 to 142.01) | 2.86 (2.55 to 3.17) | 1.88 (1.54 to 2.22) |
| Tropical Latin America | 74.64(34.59 to 138.27) | 66.35(31.25 to 116.78) | 76.05(39.22 to 126.66) | 79.16(42.82 to 126.23) | 0.05 (-0.25 to 0.35) | 0.56 (0.36 to 0.76) |
| Western Europe | 255.74(112.07 to 391.67) | 208.5(107.47 to 304.11) | 170.02(101.81 to 238.14) | 159.66(99.93 to 220.48) | -1.99 (-2.03 to -1.96) | -1.81 (-1.91 to -1.72) |
| Western Sub-Saharan Africa | 92.72(47.64 to 169.15) | 108.36(58.38 to 187.88) | 116.79(64.87 to 192.95) | 116.36(66.57 to 188.95) | 1.27 (1.13 to 1.4) | 0.85 (0.71 to 0.98) |

ASDR, age-standard DALYs rate; DALYs: disability-adjusted life years; EAPC: estimated annual percentage change.
