# Supplementary material for: Global, regional, and national burden of diet high in processed meat from 1990 to 2019: a systematic analysis from the global burden of disease study 2019
Source: Front Nutr. 2024 Feb 13;11:1354287. doi: 10.3389/fnut.2024.1354287 (PMC10896824; doi:10.3389/fnut.2024.1354287)
Supplement: Supplementary file 7 [file Table_3.docx]

Table 3S. Age-standardized SEVs of diet high in processed meat for both sexes combined in 1990, 2000,2010 and 2019, and EAPC of SEVs from 1990 to 2010 and 1990 to 2019 in 204 countries and territories

| Location | SEVs 1990 | SEVs 2000 | SEVs 2010 | SEVs 2019 | EAPC 1990to2010 | EAPC 1990to2019 |
| --- | --- | --- | --- | --- | --- | --- |
| Afghanistan | 9.74(4.2 to 20.48) | 9.38(3.96 to 19.83) | 9.3(3.87 to 18.94) | 9.54(4.08 to 20.12) | -0.22 (-0.25 to -0.19) | -0.08 (-0.13 to -0.03) |
| Albania | 81.73(60.16 to 99.43) | 83.04(60.52 to 99.54) | 83.27(60.37 to 99.98) | 81.28(54.96 to 99.98) | 0.09 (0.07 to 0.1) | 0 (-0.03 to 0.03) |
| Algeria | 11.04(4.65 to 23.72) | 12.05(5.18 to 25.05) | 12.55(5.49 to 26.26) | 12.64(5.52 to 26.1) | 0.64 (0.59 to 0.7) | 0.45 (0.39 to 0.51) |
| American Samoa | 11.9(5.14 to 25.02) | 12.13(5.31 to 25.94) | 12.23(5.27 to 26.59) | 12.21(5.33 to 26.46) | 0.13 (0.12 to 0.15) | 0.09 (0.07 to 0.1) |
| Andorra | 77.05(52.32 to 97.24) | 77.39(52.98 to 97.35) | 77.51(53.07 to 97.64) | 77.39(53.12 to 97.75) | 0.03 (0.03 to 0.03) | 0.02 (0.01 to 0.02) |
| Angola | 14.41(2.96 to 39.89) | 15.49(3.2 to 41.81) | 16.12(3.43 to 43.1) | 16.36(3.61 to 43.35) | 0.56 (0.52 to 0.6) | 0.43 (0.39 to 0.47) |
| Antigua and Barbuda | 12.89(5.73 to 26.78) | 13.29(5.83 to 27.03) | 13.54(5.98 to 28.23) | 13.7(6.07 to 28.33) | 0.25 (0.23 to 0.26) | 0.2 (0.19 to 0.22) |
| Argentina | 37.33(20.47 to 59.05) | 39.81(23.52 to 62.96) | 43.59(24.83 to 66.09) | 50.52(29.89 to 73.66) | 0.79 (0.76 to 0.83) | 1 (0.93 to 1.07) |
| Armenia | 29.01(14.61 to 49.5) | 30.36(16.34 to 50.32) | 31.44(16.24 to 52.1) | 33.4(17.97 to 54) | 0.41 (0.4 to 0.42) | 0.44 (0.42 to 0.46) |
| Australia | 67.6(42.51 to 90.85) | 71.93(46.88 to 94.14) | 74.03(47.29 to 96.84) | 74.52(48.39 to 97.04) | 0.46 (0.42 to 0.5) | 0.32 (0.28 to 0.37) |
| Austria | 62.93(38.59 to 87.48) | 72.77(49.23 to 94.37) | 75.9(49.47 to 98.37) | 72.75(46.41 to 95.81) | 0.92 (0.8 to 1.05) | 0.49 (0.35 to 0.63) |
| Azerbaijan | 44.29(24.83 to 65.72) | 46.84(27.06 to 68.36) | 49.19(29.09 to 71.4) | 51.25(30.23 to 73.34) | 0.53 (0.52 to 0.54) | 0.5 (0.49 to 0.51) |
| Bahamas | 14.25(6.14 to 29.83) | 14.38(6.2 to 30.28) | 14.45(6.17 to 30.71) | 14.49(6.34 to 31.45) | 0.07 (0.07 to 0.08) | 0.06 (0.05 to 0.06) |
| Bahrain | 12.92(5.66 to 26.98) | 13.37(5.79 to 28.93) | 13.52(5.8 to 29.16) | 13.65(5.93 to 30) | 0.23 (0.2 to 0.26) | 0.17 (0.15 to 0.19) |
| Bangladesh | 26.54(6.94 to 55.7) | 28.36(7.97 to 58.45) | 29.85(8.51 to 60.31) | 31(8.7 to 61.59) | 0.59 (0.57 to 0.61) | 0.53 (0.51 to 0.55) |
| Barbados | 16.69(7.29 to 34.83) | 16.57(7.25 to 33.86) | 16.52(7.11 to 33.47) | 16.52(7.25 to 33.89) | -0.05 (-0.05 to -0.04) | -0.03 (-0.04 to -0.03) |
| Belarus | 67.95(33.85 to 97.18) | 63.82(30.3 to 94.48) | 62.44(28.08 to 93.94) | 63.54(28.46 to 94.81) | -0.42 (-0.47 to -0.38) | -0.23 (-0.29 to -0.17) |
| Belgium | 75.61(49.95 to 97.5) | 78.22(54.32 to 98.67) | 79.66(55.71 to 99.6) | 80.12(56.95 to 99.52) | 0.26 (0.24 to 0.28) | 0.2 (0.17 to 0.22) |
| Belize | 14.19(6.19 to 30.6) | 15.31(6.68 to 32.59) | 15.79(6.81 to 33.7) | 15.72(6.8 to 33.36) | 0.54 (0.48 to 0.59) | 0.34 (0.28 to 0.4) |
| Benin | 29.68(14.96 to 49.47) | 32.84(17.04 to 54.05) | 34.26(18.45 to 56.08) | 34.29(18.27 to 55.25) | 0.72 (0.65 to 0.79) | 0.48 (0.4 to 0.56) |
| Bermuda | 15.13(6.64 to 31.38) | 14.85(6.65 to 30.72) | 14.77(6.62 to 30.77) | 14.93(6.65 to 30.57) | -0.12 (-0.13 to -0.1) | -0.05 (-0.07 to -0.03) |
| Bhutan | 29.2(8.52 to 59.31) | 31.44(10.36 to 61.33) | 33.51(11.07 to 65.06) | 35.3(12.1 to 64.31) | 0.69 (0.68 to 0.71) | 0.65 (0.64 to 0.67) |
| Bolivia (Plurinational State of) | 7.87(3.75 to 17.03) | 8.26(3.82 to 17.74) | 8.59(3.83 to 18.09) | 8.84(3.94 to 18.47) | 0.44 (0.42 to 0.45) | 0.4 (0.38 to 0.41) |
| Bosnia and Herzegovina | 45.03(25.13 to 67.89) | 48.74(28.41 to 70.64) | 51.2(29.51 to 74.31) | 52.46(30.5 to 76.9) | 0.64 (0.61 to 0.68) | 0.52 (0.48 to 0.56) |
| Botswana | 14.1(6.08 to 29.83) | 14.42(6.18 to 30.12) | 14.74(6.41 to 30.56) | 15.16(6.67 to 31.59) | 0.23 (0.22 to 0.23) | 0.24 (0.24 to 0.25) |
| Brazil | 16.69(7.58 to 34.58) | 18.94(9.16 to 36.75) | 21.62(10.62 to 41.02) | 24.63(12.13 to 44.43) | 1.32 (1.31 to 1.33) | 1.35 (1.34 to 1.36) |
| Brunei Darussalam | 36.23(19.64 to 58.23) | 37.2(20.4 to 59.23) | 37.45(20.26 to 58.94) | 36.62(19.96 to 56.95) | 0.16 (0.13 to 0.18) | 0.05 (0.01 to 0.09) |
| Bulgaria | 53.11(25.98 to 79.53) | 58.93(31.29 to 85.24) | 63.6(33.35 to 91.11) | 67.49(37.28 to 94.02) | 0.91 (0.87 to 0.94) | 0.81 (0.78 to 0.84) |
| Burkina Faso | 27.56(13.68 to 48.04) | 31(16.05 to 51.48) | 32.78(16.84 to 53.45) | 33.23(16.93 to 53.95) | 0.87 (0.8 to 0.95) | 0.62 (0.55 to 0.7) |
| Burundi | 18.62(8.35 to 37.26) | 18.52(8.55 to 36.07) | 18.45(8.52 to 35.7) | 18.48(8.44 to 36.53) | -0.05 (-0.05 to -0.05) | -0.03 (-0.04 to -0.03) |
| Cabo Verde | 29.78(14.95 to 50.12) | 30.59(15.4 to 51.13) | 31.4(15.6 to 52.27) | 32.39(16.95 to 53.25) | 0.27 (0.27 to 0.27) | 0.28 (0.28 to 0.29) |
| Cambodia | 6.76(2.55 to 20.98) | 7.15(2.65 to 23.5) | 7.39(2.51 to 23.6) | 7.51(2.56 to 23.1) | 0.45 (0.42 to 0.48) | 0.36 (0.33 to 0.39) |
| Cameroon | 32.2(16.42 to 52.78) | 32.96(17.11 to 54.18) | 33.61(17.57 to 55) | 34.29(18.28 to 55.83) | 0.22 (0.21 to 0.22) | 0.21 (0.21 to 0.21) |
| Canada | 60.22(37.7 to 83.51) | 63.82(41.34 to 85.3) | 64.93(41.55 to 86.41) | 63.5(41.08 to 85.22) | 0.37 (0.32 to 0.42) | 0.18 (0.13 to 0.24) |
| Central African Republic | 14.94(3.16 to 41.45) | 15.42(3.17 to 43.11) | 15.54(3.07 to 42.33) | 15.21(3.06 to 41.54) | 0.19 (0.16 to 0.22) | 0.07 (0.03 to 0.11) |
| Chad | 26.5(12.98 to 46.6) | 28.37(14.27 to 48.93) | 29.45(14.82 to 50.41) | 29.89(15.15 to 50.51) | 0.53 (0.49 to 0.57) | 0.41 (0.37 to 0.45) |
| Chile | 68.21(42.28 to 91.76) | 74.11(48.76 to 95.93) | 77.49(51.91 to 99.28) | 78.7(54.51 to 99.28) | 0.64 (0.6 to 0.69) | 0.48 (0.43 to 0.53) |
| China | 8.58(3.98 to 19.22) | 10.67(4.88 to 21.67) | 13.38(5.89 to 28.35) | 16.52(7.34 to 33.92) | 2.27 (2.26 to 2.29) | 2.29 (2.28 to 2.3) |
| Colombia | 13.74(5.98 to 28.94) | 15.09(6.85 to 32.35) | 16.97(7.58 to 35.67) | 19.37(8.7 to 38.93) | 1.08 (1.05 to 1.11) | 1.19 (1.15 to 1.23) |
| Comoros | 21.55(9.85 to 40.9) | 21.47(10.04 to 41.36) | 21.42(10 to 41.1) | 21.38(9.65 to 40.84) | -0.03 (-0.03 to -0.03) | -0.03 (-0.03 to -0.03) |
| Congo | 15.75(3.4 to 43.47) | 16.03(3.23 to 44.3) | 16.18(3.15 to 43.49) | 16.24(3.2 to 43.72) | 0.13 (0.12 to 0.14) | 0.1 (0.09 to 0.11) |
| Cook Islands | 11.38(4.92 to 23.63) | 12.07(5.21 to 25.74) | 12.69(5.35 to 27.3) | 13.15(5.59 to 27.87) | 0.55 (0.54 to 0.56) | 0.5 (0.48 to 0.52) |
| Costa Rica | 17.48(7.64 to 35.32) | 18.06(8.04 to 36.46) | 18.58(8.46 to 37.55) | 19.06(8.75 to 38.58) | 0.31 (0.3 to 0.31) | 0.29 (0.29 to 0.3) |
| Croatia | 14.76(4.55 to 35.28) | 13.54(4.23 to 32.35) | 13.36(3.99 to 32.44) | 15.49(4.95 to 36.7) | -0.46 (-0.55 to -0.37) | 0.06 (-0.12 to 0.23) |
| Cuba | 16.28(7.12 to 33.62) | 17.42(7.82 to 35.8) | 18.12(8.12 to 36.39) | 18.44(8.35 to 37.13) | 0.54 (0.5 to 0.57) | 0.42 (0.39 to 0.46) |
| Cyprus | 61.95(37.9 to 85.3) | 57.3(35.57 to 79.41) | 56.37(33.56 to 80.53) | 59.78(36.15 to 83.45) | -0.45 (-0.52 to -0.37) | -0.14 (-0.24 to -0.04) |
| Czechia | 50.75(28.74 to 74.64) | 52.15(30.75 to 75.67) | 53.31(31.37 to 77.27) | 54.28(32.71 to 78.85) | 0.25 (0.24 to 0.25) | 0.23 (0.22 to 0.24) |
| C么te d'Ivoire | 35.39(18.8 to 55.63) | 35.83(19.41 to 56.18) | 36.22(19.4 to 56.68) | 37.13(19.88 to 59.41) | 0.12 (0.11 to 0.12) | 0.15 (0.13 to 0.16) |
| Democratic People's Republic of Korea | 11.87(4.99 to 24.28) | 12.28(5.22 to 25.72) | 12.45(5.27 to 26.55) | 12.43(5.17 to 26.73) | 0.24 (0.21 to 0.26) | 0.15 (0.13 to 0.18) |
| Democratic Republic of the Congo | 17.32(3.61 to 44.7) | 15.53(3.17 to 42.55) | 14.93(2.93 to 41.88) | 15.41(2.95 to 43.49) | -0.75 (-0.83 to -0.66) | -0.41 (-0.52 to -0.31) |
| Denmark | 76.28(50.07 to 98.15) | 78.5(54.51 to 98.98) | 79.49(55.56 to 99.56) | 79.48(55.34 to 99.41) | 0.21 (0.19 to 0.23) | 0.14 (0.12 to 0.16) |
| Djibouti | 16.86(7.37 to 33.63) | 18.47(8.37 to 36.54) | 19.42(8.89 to 38.69) | 19.8(9.09 to 38.97) | 0.71 (0.66 to 0.76) | 0.54 (0.49 to 0.6) |
| Dominica | 15.54(6.7 to 32.5) | 16.37(7.39 to 33.85) | 16.72(7.38 to 33.85) | 16.64(7.51 to 34.5) | 0.37 (0.33 to 0.4) | 0.23 (0.19 to 0.27) |
| Dominican Republic | 13.9(5.97 to 29.06) | 14.49(6.37 to 31.06) | 15.1(6.65 to 31.7) | 15.78(6.75 to 33.03) | 0.42 (0.42 to 0.42) | 0.43 (0.43 to 0.44) |
| Ecuador | 17.79(7.93 to 35.13) | 18.59(8.65 to 35.9) | 19.29(9.05 to 37.63) | 20.18(9.69 to 38.48) | 0.41 (0.4 to 0.42) | 0.41 (0.41 to 0.42) |
| Egypt | 12(5.01 to 25.57) | 14.12(6.35 to 29.85) | 16.07(7.12 to 34.48) | 17.73(7.95 to 37.16) | 1.47 (1.43 to 1.51) | 1.34 (1.3 to 1.38) |
| El Salvador | 15.49(6.55 to 33.09) | 16.46(7.14 to 34.74) | 17.1(7.71 to 34.89) | 17.44(7.61 to 34.56) | 0.5 (0.47 to 0.52) | 0.4 (0.37 to 0.43) |
| Equatorial Guinea | 15.06(3.06 to 42.56) | 18.43(4.06 to 47.19) | 19.81(4.46 to 48.92) | 19.68(4.42 to 48.39) | 1.37 (1.22 to 1.53) | 0.87 (0.71 to 1.03) |
| Eritrea | 18.45(8.01 to 37.09) | 19.17(8.49 to 37.94) | 19.56(8.88 to 38.59) | 19.69(8.86 to 38.5) | 0.3 (0.27 to 0.32) | 0.22 (0.2 to 0.24) |
| Estonia | 86.42(65.1 to 100) | 89.85(76.07 to 100) | 90.68(77.99 to 100) | 88.57(71.83 to 100) | 0.23 (0.19 to 0.27) | 0.09 (0.05 to 0.14) |
| Eswatini | 15.51(6.73 to 31.96) | 15.48(6.85 to 32.58) | 15.5(6.82 to 32.77) | 15.71(6.74 to 32.95) | 0 (-0.01 to 0) | 0.03 (0.02 to 0.04) |
| Ethiopia | 16.42(7.26 to 33.28) | 16.87(7.65 to 34.31) | 17.48(7.77 to 35.5) | 18.35(8.36 to 36.24) | 0.32 (0.31 to 0.33) | 0.37 (0.35 to 0.39) |
| Fiji | 10.48(4.39 to 21.88) | 11.08(4.76 to 22.86) | 11.42(4.82 to 24.3) | 11.56(4.93 to 24.34) | 0.43 (0.4 to 0.46) | 0.33 (0.3 to 0.36) |
| Finland | 66.24(40.76 to 89.79) | 76.4(53.12 to 95.76) | 78.86(54 to 99.27) | 72.63(45.89 to 95.88) | 0.83 (0.69 to 0.96) | 0.33 (0.16 to 0.49) |
| France | 76.61(51.69 to 98.09) | 78.87(55.32 to 98.98) | 79.89(56.32 to 99.71) | 79.9(56.3 to 99.59) | 0.21 (0.19 to 0.23) | 0.14 (0.12 to 0.16) |
| Gabon | 16.98(3.81 to 45.17) | 17.78(4.02 to 46.2) | 18.18(3.81 to 47.23) | 18.26(4.09 to 46.51) | 0.34 (0.32 to 0.37) | 0.25 (0.21 to 0.28) |
| Gambia | 32.79(16.46 to 53.28) | 33.68(17.64 to 55.25) | 34.1(17.97 to 54.93) | 33.98(18.16 to 54.02) | 0.2 (0.18 to 0.22) | 0.13 (0.1 to 0.15) |
| Georgia | 46.96(26.49 to 71.15) | 46.79(26.89 to 69.06) | 46.93(26.26 to 69.85) | 48.84(28.15 to 71.92) | 0 (-0.01 to 0.01) | 0.1 (0.06 to 0.13) |
| Germany | 80.16(56.37 to 99.54) | 84.11(64.99 to 99.78) | 85.2(66.68 to 99.96) | 83.27(62.56 to 99.89) | 0.3 (0.25 to 0.34) | 0.14 (0.08 to 0.19) |
| Ghana | 29.95(15.55 to 50.95) | 35.36(19.09 to 55.56) | 38.61(21.24 to 59.76) | 39.87(21.89 to 60.8) | 1.27 (1.18 to 1.37) | 0.96 (0.86 to 1.06) |
| Greece | 54.39(32.47 to 77.49) | 62.03(39.68 to 84.21) | 64.81(39.22 to 88.54) | 63.37(38.91 to 87.52) | 0.87 (0.76 to 0.98) | 0.51 (0.39 to 0.62) |
| Greenland | 78.18(54.34 to 97.91) | 80.19(57.73 to 98.72) | 81.31(59.75 to 99.31) | 81.68(59.85 to 99.4) | 0.2 (0.18 to 0.21) | 0.15 (0.13 to 0.16) |
| Grenada | 13.59(6.14 to 28.47) | 13.76(6.13 to 29.06) | 13.97(6.07 to 30.43) | 14.33(6.18 to 30.72) | 0.14 (0.14 to 0.15) | 0.17 (0.16 to 0.18) |
| Guam | 12.94(5.48 to 26.66) | 13.55(5.85 to 27.84) | 13.91(5.96 to 28.8) | 14.05(6.22 to 29.32) | 0.37 (0.35 to 0.4) | 0.28 (0.26 to 0.31) |
| Guatemala | 15.12(6.6 to 31.77) | 15.93(7.23 to 33.47) | 16.45(7.47 to 34.99) | 16.68(7.49 to 34.71) | 0.42 (0.4 to 0.45) | 0.34 (0.31 to 0.36) |
| Guinea | 32.02(16.4 to 53.44) | 32.82(17.51 to 53.71) | 33.44(17.77 to 54.92) | 33.93(17.72 to 55.59) | 0.22 (0.21 to 0.23) | 0.2 (0.19 to 0.2) |
| Guinea-Bissau | 29.11(14.48 to 50.66) | 29.32(14.7 to 50.11) | 29.57(14.85 to 50.29) | 30.04(15.46 to 50.86) | 0.08 (0.08 to 0.08) | 0.1 (0.09 to 0.11) |
| Guyana | 14.75(6.4 to 31.22) | 15.5(6.82 to 32.53) | 16.06(6.96 to 33.62) | 16.46(6.92 to 34.27) | 0.43 (0.41 to 0.44) | 0.37 (0.36 to 0.39) |
| Haiti | 12.03(5.06 to 26.04) | 12.4(5.32 to 27.08) | 12.63(5.5 to 28.21) | 12.76(5.53 to 28.2) | 0.24 (0.23 to 0.26) | 0.2 (0.18 to 0.21) |
| Honduras | 15.24(6.69 to 31.74) | 16.22(7.19 to 33.61) | 16.81(7.42 to 34.85) | 17.07(7.52 to 35.26) | 0.49 (0.46 to 0.52) | 0.38 (0.35 to 0.42) |
| Hungary | 49.78(24.12 to 76.25) | 58.96(32.12 to 85.04) | 63.29(32.98 to 90.69) | 64.05(33.84 to 91.39) | 1.21 (1.09 to 1.33) | 0.83 (0.7 to 0.95) |
| Iceland | 75.27(50.58 to 96.43) | 74.48(48.93 to 95.78) | 74.27(49 to 95.72) | 74.92(50.7 to 96.22) | -0.06 (-0.07 to -0.06) | -0.02 (-0.04 to -0.01) |
| India | 9.96(4.68 to 22.83) | 11.44(5.4 to 26.12) | 12.52(5.97 to 27.77) | 13.2(6.43 to 28.33) | 1.15 (1.09 to 1.21) | 0.96 (0.9 to 1.02) |
| Indonesia | 5.11(2.29 to 14.69) | 5.28(2.41 to 14.68) | 5.6(2.52 to 15.47) | 6.32(2.84 to 16.77) | 0.47 (0.43 to 0.5) | 0.68 (0.61 to 0.76) |
| Iran (Islamic Republic of) | 11.88(5.36 to 25.09) | 12.4(5.53 to 26.3) | 12.68(5.61 to 26.71) | 12.73(5.58 to 26.8) | 0.32 (0.3 to 0.35) | 0.24 (0.21 to 0.26) |
| Iraq | 10.01(2.38 to 28.05) | 9.56(2.35 to 26.03) | 9.46(2.38 to 25.7) | 9.79(2.39 to 27.72) | -0.27 (-0.31 to -0.23) | -0.09 (-0.15 to -0.03) |
| Ireland | 63.12(37.84 to 87.66) | 72.81(48.31 to 93.89) | 75.91(49.85 to 98.13) | 72.87(46.45 to 95.61) | 0.91 (0.78 to 1.03) | 0.48 (0.34 to 0.62) |
| Israel | 44.82(25.08 to 67.58) | 47.59(28.9 to 70.38) | 50.58(29.3 to 74.58) | 55.27(32.48 to 79.67) | 0.61 (0.61 to 0.62) | 0.69 (0.66 to 0.71) |
| Italy | 69.24(44.71 to 90.72) | 73.25(49.99 to 93.23) | 74.84(50.55 to 95.19) | 74.37(50.47 to 95.02) | 0.39 (0.35 to 0.43) | 0.24 (0.19 to 0.29) |
| Jamaica | 15.82(7.03 to 31.59) | 16.38(7.22 to 33.81) | 16.56(7.1 to 33.99) | 16.33(7.1 to 33.72) | 0.22 (0.19 to 0.25) | 0.11 (0.08 to 0.15) |
| Japan | 64.51(38.61 to 87.68) | 73.13(49.19 to 93.17) | 75.4(50.2 to 96.21) | 70.87(45.32 to 93.01) | 0.75 (0.63 to 0.86) | 0.33 (0.19 to 0.47) |
| Jordan | 9.98(2.36 to 31) | 10.53(2.4 to 32.59) | 10.78(2.38 to 32.68) | 10.77(2.45 to 31.93) | 0.39 (0.35 to 0.42) | 0.26 (0.22 to 0.3) |
| Kazakhstan | 54.59(33.52 to 77.89) | 53.56(32.07 to 75.88) | 53.4(31.44 to 75.24) | 55.46(33.62 to 78.92) | -0.1 (-0.12 to -0.08) | 0.02 (-0.02 to 0.07) |
| Kenya | 20.11(9.74 to 36.84) | 20.65(10.25 to 37.91) | 21.09(10.38 to 39.05) | 21.54(10.46 to 39.68) | 0.24 (0.24 to 0.25) | 0.23 (0.23 to 0.24) |
| Kiribati | 9.66(4.09 to 18.96) | 10.25(4.3 to 21.28) | 10.55(4.47 to 22.05) | 10.64(4.34 to 22.43) | 0.45 (0.41 to 0.48) | 0.33 (0.29 to 0.36) |
| Kuwait | 10.78(2.47 to 30.15) | 12.05(2.6 to 34) | 12.45(2.62 to 33.57) | 12.01(2.61 to 33.48) | 0.71 (0.61 to 0.8) | 0.37 (0.26 to 0.48) |
| Kyrgyzstan | 49.96(28.42 to 73.43) | 48.87(28.07 to 72.38) | 48.66(28.21 to 72.27) | 49.85(29.2 to 73.49) | -0.13 (-0.15 to -0.1) | -0.02 (-0.06 to 0.01) |
| Lao People's Democratic Republic | 6.8(3.39 to 15.86) | 7.26(3.58 to 16.71) | 7.64(3.65 to 17.97) | 7.94(3.7 to 18.83) | 0.59 (0.57 to 0.6) | 0.53 (0.51 to 0.55) |
| Latvia | 90.94(76.13 to 100) | 93.65(84.85 to 99.99) | 94.12(85.85 to 100) | 91.69(77.69 to 100) | 0.16 (0.13 to 0.19) | 0.04 (0 to 0.08) |
| Lebanon | 10.44(2.41 to 30.51) | 10.61(2.46 to 29.92) | 10.68(2.48 to 30.17) | 10.68(2.44 to 30.11) | 0.11 (0.1 to 0.12) | 0.08 (0.06 to 0.09) |
| Lesotho | 15.88(6.68 to 32.67) | 16.85(7.16 to 34.85) | 17.42(7.34 to 36.51) | 17.64(7.75 to 37.04) | 0.47 (0.43 to 0.5) | 0.36 (0.32 to 0.39) |
| Liberia | 32.92(16.99 to 53.64) | 31.37(16.49 to 52.18) | 30.84(15.92 to 51.73) | 31.4(15.97 to 52.2) | -0.33 (-0.36 to -0.29) | -0.17 (-0.22 to -0.12) |
| Libya | 10.99(2.52 to 30.28) | 11.09(2.57 to 30.56) | 11.06(2.48 to 31.82) | 10.49(2.51 to 30.85) | 0.03 (0.01 to 0.04) | -0.11 (-0.16 to -0.06) |
| Lithuania | 91.66(78.54 to 100) | 94.94(88.71 to 99.99) | 95.63(89.59 to 100) | 93.26(80.98 to 100) | 0.2 (0.17 to 0.23) | 0.07 (0.02 to 0.11) |
| Luxembourg | 85.25(66.53 to 99.73) | 89.31(75.14 to 99.75) | 90.12(77.84 to 99.85) | 87.01(70.74 to 99.77) | 0.26 (0.21 to 0.31) | 0.08 (0.02 to 0.14) |
| Madagascar | 20.21(9.38 to 39.62) | 19.69(9.09 to 38.75) | 19.52(8.94 to 38.25) | 19.75(9 to 38.88) | -0.17 (-0.19 to -0.15) | -0.08 (-0.11 to -0.06) |
| Malawi | 19.26(8.39 to 38.22) | 20.59(9.42 to 39.55) | 21.2(10.03 to 40.12) | 21.18(10.01 to 40.49) | 0.48 (0.44 to 0.53) | 0.32 (0.27 to 0.37) |
| Malaysia | 13.74(5.93 to 28.41) | 15.6(7.2 to 32.57) | 17.59(7.86 to 36.87) | 19.6(8.87 to 39.17) | 1.25 (1.24 to 1.26) | 1.23 (1.22 to 1.23) |
| Maldives | 7.09(3.49 to 16.73) | 7.37(3.57 to 17.18) | 7.59(3.57 to 17.32) | 7.75(3.61 to 16.94) | 0.34 (0.33 to 0.35) | 0.3 (0.29 to 0.31) |
| Mali | 31.36(15.69 to 53.14) | 35.19(18.63 to 56.52) | 36.78(19.8 to 57.93) | 36.65(19.75 to 57.67) | 0.8 (0.71 to 0.89) | 0.51 (0.42 to 0.6) |
| Malta | 83.33(63.19 to 99.54) | 85.8(68.88 to 99.7) | 86.36(68.99 to 99.82) | 84.71(66.4 to 99.77) | 0.17 (0.14 to 0.2) | 0.06 (0.03 to 0.1) |
| Marshall Islands | 9.85(4.2 to 21.38) | 10.17(4.42 to 21.62) | 10.41(4.35 to 22.12) | 10.59(4.49 to 22.59) | 0.27 (0.26 to 0.28) | 0.25 (0.24 to 0.25) |
| Mauritania | 34.68(17.94 to 55.52) | 37.12(20.47 to 58.3) | 38.41(21.6 to 60.11) | 38.68(22.23 to 60.66) | 0.52 (0.47 to 0.56) | 0.37 (0.32 to 0.42) |
| Mauritius | 8.45(4 to 18.56) | 8.91(3.97 to 18.43) | 9.22(3.99 to 18.75) | 9.4(4.05 to 20.2) | 0.44 (0.42 to 0.47) | 0.36 (0.34 to 0.39) |
| Mexico | 21.88(11.39 to 38.94) | 23.07(12.12 to 40.22) | 23.78(12.38 to 40.93) | 24.1(12.59 to 40.59) | 0.42 (0.39 to 0.45) | 0.33 (0.3 to 0.36) |
| Micronesia (Federated States of) | 9.59(4.07 to 19.61) | 10(4.3 to 20.65) | 10.22(4.34 to 21.45) | 10.28(4.42 to 21.72) | 0.32 (0.29 to 0.34) | 0.23 (0.21 to 0.26) |
| Monaco | 80.48(58.15 to 98.86) | 80.71(58.14 to 98.87) | 81.05(59.02 to 99.07) | 81.67(60.25 to 99.16) | 0.04 (0.03 to 0.04) | 0.05 (0.04 to 0.05) |
| Mongolia | 46.55(26.05 to 69.02) | 44.76(25.21 to 66.79) | 44.52(24.8 to 66.51) | 47.69(27.23 to 70.55) | -0.2 (-0.25 to -0.16) | 0.03 (-0.05 to 0.11) |
| Montenegro | 68.22(42.61 to 91.87) | 71.46(46.73 to 93.53) | 72.11(45.8 to 95.05) | 69.63(44.65 to 92.8) | 0.26 (0.21 to 0.31) | 0.08 (0.02 to 0.14) |
| Morocco | 7.22(2.28 to 19.71) | 7.9(2.22 to 21.14) | 8.86(2.05 to 24.89) | 10.09(2.18 to 29.32) | 1.05 (1.01 to 1.08) | 1.16 (1.12 to 1.2) |
| Mozambique | 17.64(7.83 to 35.75) | 19.01(8.45 to 38.55) | 19.91(8.94 to 39.32) | 20.39(9.2 to 39.95) | 0.61 (0.57 to 0.64) | 0.49 (0.46 to 0.53) |
| Myanmar | 6.52(3.26 to 14.73) | 7.2(3.48 to 16.7) | 7.7(3.53 to 17.22) | 8.03(3.76 to 18.34) | 0.83 (0.79 to 0.87) | 0.71 (0.67 to 0.75) |
| Namibia | 14.13(5.94 to 28.85) | 14.04(6.02 to 29.19) | 14.05(6.02 to 30.51) | 14.32(6.2 to 31.19) | -0.03 (-0.04 to -0.02) | 0.03 (0.01 to 0.05) |
| Nauru | 11.38(4.85 to 24.31) | 10.67(4.52 to 23.08) | 10.58(4.4 to 22.68) | 11.48(4.86 to 24.81) | -0.34 (-0.41 to -0.27) | -0.01 (-0.12 to 0.09) |
| Nepal | 34.22(18.32 to 54.46) | 37.87(20.72 to 58.61) | 40.31(21.8 to 61.91) | 41.57(22.85 to 63.15) | 0.82 (0.77 to 0.87) | 0.66 (0.61 to 0.71) |
| Netherlands | 67.95(42.96 to 90.93) | 71.55(46.89 to 93.81) | 73.42(47 to 96.39) | 73.98(48.04 to 96.54) | 0.39 (0.36 to 0.42) | 0.28 (0.25 to 0.32) |
| New Zealand | 64.64(40.06 to 87.73) | 69.36(44.21 to 91.51) | 71.9(46 to 94.84) | 72.83(47.51 to 95.26) | 0.54 (0.5 to 0.58) | 0.4 (0.36 to 0.44) |
| Nicaragua | 14.26(5.96 to 30.62) | 15.5(6.55 to 32.85) | 16.25(6.99 to 33.97) | 16.56(7.18 to 33.56) | 0.65 (0.61 to 0.7) | 0.51 (0.46 to 0.55) |
| Niger | 31.18(16.05 to 50.96) | 33.52(17.76 to 54.1) | 34.64(18.21 to 55.71) | 34.62(18.09 to 56.15) | 0.53 (0.48 to 0.58) | 0.35 (0.3 to 0.41) |
| Nigeria | 30.48(15.59 to 50.68) | 33.93(18.31 to 55.34) | 35.83(19.52 to 57.3) | 36.34(19.84 to 57.98) | 0.81 (0.75 to 0.88) | 0.59 (0.52 to 0.66) |
| Niue | 10.8(4.55 to 24.33) | 11.25(4.8 to 24.25) | 11.59(4.95 to 25.2) | 11.84(4.99 to 26.07) | 0.35 (0.34 to 0.36) | 0.31 (0.3 to 0.32) |
| North Macedonia | 47.79(26.41 to 71.21) | 50.23(28.95 to 72.77) | 51.9(30.31 to 75.22) | 52.87(30.64 to 76.67) | 0.41 (0.39 to 0.44) | 0.34 (0.32 to 0.37) |
| Northern Mariana Islands | 12.69(5.31 to 26.39) | 12.88(5.59 to 27.16) | 12.93(5.59 to 27.45) | 12.94(5.69 to 26.72) | 0.08 (0.06 to 0.1) | 0.05 (0.04 to 0.07) |
| Norway | 89.68(76.15 to 99.87) | 92.27(82.16 to 99.84) | 93.18(85.04 to 99.95) | 92.59(83.41 to 99.94) | 0.19 (0.17 to 0.21) | 0.11 (0.08 to 0.13) |
| Oman | 10.06(4.28 to 22.03) | 11.19(4.78 to 22.78) | 11.55(4.77 to 24.49) | 10.57(2.52 to 30.22) | 0.65 (0.56 to 0.75) | 0.23 (0.09 to 0.37) |
| Pakistan | 37.36(20.89 to 57.61) | 37.69(21.66 to 58.08) | 38.12(21.58 to 58.82) | 38.94(22.2 to 59.97) | 0.1 (0.1 to 0.11) | 0.13 (0.12 to 0.14) |
| Palau | 10.94(4.67 to 23.03) | 11.28(4.71 to 23.84) | 11.62(4.78 to 25.31) | 11.78(4.89 to 26.18) | 0.28 (0.27 to 0.29) | 0.25 (0.24 to 0.27) |
| Palestine | 8.86(2.39 to 26.08) | 8.8(2.31 to 24.51) | 8.79(2.37 to 23.97) | 8.93(2.32 to 24.48) | -0.04 (-0.05 to -0.03) | 0.01 (0 to 0.03) |
| Panama | 14.68(6.28 to 29.96) | 14.98(6.51 to 31.8) | 15.49(6.6 to 32.87) | 16.41(7.2 to 34.53) | 0.28 (0.26 to 0.29) | 0.37 (0.34 to 0.4) |
| Papua New Guinea | 9.28(4 to 19.7) | 9.3(4.01 to 19.38) | 9.35(4.12 to 19.33) | 9.57(4.16 to 20.38) | 0.04 (0.03 to 0.04) | 0.09 (0.07 to 0.1) |
| Paraguay | 15.94(6.83 to 33.72) | 16.16(7.06 to 34.34) | 16.47(7.3 to 34.79) | 17.05(7.64 to 35.41) | 0.17 (0.16 to 0.17) | 0.22 (0.2 to 0.23) |
| Peru | 6.41(3.16 to 15.08) | 7.01(3.31 to 16.03) | 7.86(3.26 to 16.09) | 8.98(3.76 to 18.73) | 1.04 (1.01 to 1.07) | 1.16 (1.12 to 1.2) |
| Philippines | 13.84(5.92 to 29.22) | 15.31(6.8 to 32.71) | 17.11(7.54 to 36.29) | 19.24(8.99 to 38.34) | 1.08 (1.07 to 1.1) | 1.14 (1.12 to 1.16) |
| Poland | 24.79(9.54 to 49.88) | 33.15(14.86 to 57.93) | 37.49(17.09 to 63.15) | 38.38(17.86 to 63.74) | 2.05 (1.85 to 2.26) | 1.43 (1.23 to 1.63) |
| Portugal | 37.18(20.5 to 59.21) | 45.52(27.99 to 65.73) | 48.67(28.31 to 72.46) | 47.09(26.8 to 70.32) | 1.32 (1.16 to 1.49) | 0.78 (0.6 to 0.95) |
| Puerto Rico | 15.87(6.92 to 32.82) | 16.65(7.28 to 33.59) | 17.05(7.49 to 33.79) | 17.14(7.57 to 35.46) | 0.36 (0.33 to 0.39) | 0.26 (0.23 to 0.29) |
| Qatar | 12.17(2.65 to 35.17) | 12.68(2.82 to 37.04) | 12.98(2.89 to 38.21) | 13.12(2.91 to 37.45) | 0.32 (0.3 to 0.34) | 0.25 (0.23 to 0.28) |
| Republic of Korea | 24.48(6.29 to 54.42) | 36.93(13.59 to 65.82) | 40.69(14.4 to 70.88) | 35.81(12.19 to 66.68) | 2.41 (2.02 to 2.8) | 1.2 (0.81 to 1.6) |
| Republic of Moldova | 65.54(32.21 to 95.4) | 61.65(28.73 to 91.97) | 60.22(26.7 to 90.7) | 60.91(27.26 to 92.07) | -0.43 (-0.47 to -0.38) | -0.25 (-0.31 to -0.2) |
| Romania | 34.28(10.63 to 64.98) | 53.61(32.2 to 76.2) | 64.73(38.69 to 88) | 68.93(42.28 to 92.68) | 3.13 (2.81 to 3.46) | 2.25 (1.96 to 2.54) |
| Russian Federation | 95.37(90.32 to 99.92) | 89.22(77.45 to 97.77) | 80.64(58.6 to 99.15) | 69.73(45.29 to 91.47) | -0.84 (-0.88 to -0.8) | -1.04 (-1.11 to -0.98) |
| Rwanda | 18.39(8.41 to 37.38) | 18.77(8.72 to 37.34) | 19.21(8.54 to 37.73) | 19.84(9.11 to 38.95) | 0.22 (0.22 to 0.23) | 0.25 (0.24 to 0.26) |
| Saint Kitts and Nevis | 14.49(6.34 to 29.99) | 14.25(6.32 to 29.71) | 14.21(6.27 to 29.81) | 14.66(6.28 to 30.45) | -0.09 (-0.11 to -0.07) | 0.02 (-0.02 to 0.05) |
| Saint Lucia | 14.36(6.11 to 29.13) | 14.6(6.31 to 30.8) | 14.78(6.47 to 31.37) | 14.93(6.46 to 32.01) | 0.14 (0.14 to 0.15) | 0.13 (0.13 to 0.13) |
| Saint Vincent and the Grenadines | 13.96(6.07 to 29.83) | 15.35(6.82 to 32.37) | 16.02(6.96 to 32.89) | 16.11(7.04 to 33.07) | 0.69 (0.63 to 0.75) | 0.48 (0.41 to 0.55) |
| Samoa | 10.87(4.74 to 23.42) | 11.58(5.08 to 24.29) | 11.9(5.21 to 25.27) | 11.9(5.11 to 24.68) | 0.45 (0.41 to 0.5) | 0.3 (0.25 to 0.35) |
| San Marino | 76.57(53.1 to 97.18) | 77.58(54.63 to 97.35) | 77.81(54.18 to 97.84) | 76.86(53.19 to 97.11) | 0.08 (0.06 to 0.09) | 0.02 (0 to 0.04) |
| Sao Tome and Principe | 33.31(17.64 to 54.09) | 34.48(18.69 to 55.35) | 35.15(18.62 to 57.17) | 35.5(18.68 to 57.07) | 0.27 (0.25 to 0.29) | 0.21 (0.19 to 0.23) |
| Saudi Arabia | 9.6(2.24 to 27.59) | 10.95(4.69 to 24.27) | 11.4(4.7 to 24.33) | 11.24(2.35 to 32.84) | 0.82 (0.71 to 0.93) | 0.5 (0.39 to 0.61) |
| Senegal | 32.58(17.01 to 53.52) | 32.97(17.99 to 53.79) | 33.33(17.48 to 55.16) | 35.03(18.1 to 56.65) | 0.12 (0.12 to 0.12) | 0.2 (0.17 to 0.23) |
| Serbia | 42.85(20.42 to 67.48) | 40.25(19.67 to 63.56) | 39.86(18.3 to 66.01) | 42.56(20.07 to 68.67) | -0.33 (-0.4 to -0.27) | -0.05 (-0.15 to 0.04) |
| Seychelles | 6.91(3.31 to 16.24) | 7.45(2.83 to 21.23) | 7.64(2.7 to 22.07) | 7.75(3.48 to 17.49) | 0.49 (0.43 to 0.55) | 0.35 (0.3 to 0.4) |
| Sierra Leone | 27.79(13.7 to 48.65) | 28.36(14.02 to 48.51) | 28.82(14.2 to 49.16) | 29.38(14.44 to 49.98) | 0.18 (0.18 to 0.19) | 0.18 (0.18 to 0.18) |
| Singapore | 16.41(3.36 to 43.82) | 19.06(4.35 to 47.74) | 21.96(4.93 to 52.46) | 24.89(6.31 to 55.55) | 1.47 (1.46 to 1.48) | 1.44 (1.43 to 1.45) |
| Slovakia | 50.9(30.71 to 73.34) | 61.09(39.85 to 83.58) | 67.3(41.32 to 90.72) | 70.2(44.31 to 92.87) | 1.4 (1.29 to 1.5) | 1.07 (0.97 to 1.18) |
| Slovenia | 80.6(58.96 to 99.17) | 84.2(65.81 to 99.59) | 85.14(66.87 to 99.84) | 83.25(62.91 to 99.69) | 0.27 (0.23 to 0.31) | 0.12 (0.07 to 0.16) |
| Solomon Islands | 8.21(2.4 to 22.44) | 8.47(2.4 to 23.16) | 8.64(2.34 to 23.83) | 8.74(2.34 to 24.12) | 0.26 (0.25 to 0.27) | 0.21 (0.2 to 0.23) |
| Somalia | 18.19(8.04 to 36.75) | 17.93(7.86 to 36.45) | 17.76(7.7 to 35.69) | 17.65(7.53 to 36.28) | -0.12 (-0.13 to -0.12) | -0.1 (-0.11 to -0.09) |
| South Africa | 16.36(7.18 to 33.65) | 17.79(8.17 to 35.66) | 19(8.78 to 37.69) | 19.99(9.41 to 39.23) | 0.76 (0.73 to 0.78) | 0.69 (0.66 to 0.71) |
| South Sudan | 22.65(10.63 to 41.58) | 22.98(10.66 to 42.42) | 23.21(10.74 to 43.63) | 23.35(11.26 to 43.23) | 0.12 (0.12 to 0.13) | 0.1 (0.1 to 0.11) |
| Spain | 83.43(63.67 to 99.77) | 79.66(56.37 to 99.38) | 78.23(53.13 to 99.22) | 78.8(54.56 to 99.11) | -0.33 (-0.36 to -0.29) | -0.2 (-0.24 to -0.16) |
| Sri Lanka | 7.48(2.52 to 22.13) | 7.75(2.59 to 21.7) | 7.95(2.56 to 23.12) | 8.12(2.5 to 22.42) | 0.31 (0.3 to 0.32) | 0.28 (0.27 to 0.29) |
| Sudan | 9.07(2.41 to 25) | 9.28(2.44 to 26.4) | 9.46(2.39 to 26.67) | 9.65(2.33 to 27.52) | 0.21 (0.2 to 0.21) | 0.2 (0.2 to 0.21) |
| Suriname | 15.37(6.82 to 32.93) | 15.73(6.9 to 32.76) | 16.05(6.89 to 33.14) | 16.37(7.06 to 34.12) | 0.22 (0.21 to 0.22) | 0.21 (0.21 to 0.21) |
| Sweden | 81.02(59.56 to 99.03) | 83.73(65.81 to 99.44) | 85.42(67.49 to 99.8) | 86.2(69.89 to 99.66) | 0.27 (0.25 to 0.28) | 0.21 (0.19 to 0.23) |
| Switzerland | 71.36(45.93 to 94.57) | 73.87(48.46 to 96.04) | 75.26(48.45 to 97.89) | 75.85(49.87 to 97.52) | 0.27 (0.25 to 0.29) | 0.21 (0.18 to 0.23) |
| Syrian Arab Republic | 10.41(2.35 to 29.83) | 10.72(2.45 to 31.12) | 10.78(2.45 to 31.61) | 10.42(2.38 to 30.19) | 0.16 (0.13 to 0.19) | 0.02 (-0.02 to 0.07) |
| Taiwan (Province of China) | 17.86(7.94 to 36.93) | 20.72(10.12 to 39.45) | 23.65(11.29 to 44.08) | 26.42(12.77 to 47.24) | 1.42 (1.4 to 1.44) | 1.35 (1.33 to 1.37) |
| Tajikistan | 37(20.39 to 58.52) | 35.33(19.69 to 56.83) | 34.99(19.3 to 56.81) | 36.51(19.96 to 58.22) | -0.27 (-0.31 to -0.22) | -0.07 (-0.13 to 0) |
| Thailand | 5.87(2.65 to 15.68) | 6.52(2.74 to 17.61) | 7.26(2.74 to 17.24) | 8.05(3.01 to 19.35) | 1.08 (1.07 to 1.08) | 1.09 (1.09 to 1.1) |
| Timor-Leste | 6.63(2.87 to 16.85) | 6.97(3.04 to 19.08) | 7.15(3.07 to 18.54) | 7.19(3.13 to 18.72) | 0.38 (0.35 to 0.41) | 0.27 (0.24 to 0.31) |
| Togo | 28.09(13.75 to 48.34) | 30.39(15.53 to 50.8) | 31.65(16.21 to 52.98) | 32.02(16.6 to 52.5) | 0.6 (0.55 to 0.65) | 0.44 (0.39 to 0.49) |
| Tokelau | 9.07(2.43 to 24.16) | 9.32(2.41 to 25.38) | 9.62(2.37 to 25.31) | 10.56(4.56 to 21.03) | 0.29 (0.29 to 0.3) | 0.45 (0.4 to 0.51) |
| Tonga | 9.08(2.36 to 26.52) | 9.37(2.39 to 27.21) | 9.55(2.26 to 27) | 9.62(2.36 to 27.18) | 0.25 (0.24 to 0.27) | 0.2 (0.18 to 0.21) |
| Trinidad and Tobago | 16.08(7.08 to 33.65) | 16.7(7.43 to 35.4) | 17.16(7.65 to 35.84) | 17.48(7.96 to 35.51) | 0.33 (0.32 to 0.34) | 0.28 (0.27 to 0.3) |
| Tunisia | 10.87(2.51 to 31.49) | 11.23(2.49 to 31.61) | 11.57(2.41 to 31.9) | 12.55(4.66 to 28.61) | 0.31 (0.31 to 0.31) | 0.43 (0.38 to 0.47) |
| Turkey | 9.56(2.21 to 26.97) | 10.05(2.3 to 28.19) | 10.82(2.29 to 30.95) | 11.92(2.43 to 34.37) | 0.63 (0.6 to 0.66) | 0.75 (0.71 to 0.79) |
| Turkmenistan | 50.5(29.3 to 73.78) | 51.36(30.45 to 74.5) | 53.02(32.03 to 76.59) | 56.35(34.04 to 80.07) | 0.25 (0.23 to 0.27) | 0.36 (0.32 to 0.39) |
| Tuvalu | 8.81(2.35 to 24.64) | 9.09(2.41 to 25.81) | 9.28(2.43 to 27.03) | 9.41(2.42 to 27.18) | 0.26 (0.25 to 0.27) | 0.22 (0.21 to 0.23) |
| Uganda | 18.81(8.39 to 37.57) | 19.17(8.67 to 38.63) | 19.49(8.98 to 38.98) | 19.83(9.03 to 39.95) | 0.18 (0.17 to 0.18) | 0.18 (0.17 to 0.18) |
| Ukraine | 30.35(15.68 to 51.25) | 34.22(19.4 to 54.9) | 37.78(20.95 to 60.31) | 41.25(23.56 to 63.96) | 1.11 (1.08 to 1.13) | 1.04 (1.02 to 1.06) |
| United Arab Emirates | 19.38(4.29 to 49.01) | 17.69(3.77 to 46.63) | 16.03(3.14 to 44.66) | 14.54(2.83 to 42.79) | -0.94 (-0.95 to -0.94) | -0.98 (-0.99 to -0.97) |
| United Kingdom | 79.24(58.52 to 96.44) | 82.35(63.76 to 97.6) | 83.77(64.98 to 98.73) | 83.75(64.92 to 98.53) | 0.28 (0.25 to 0.31) | 0.19 (0.16 to 0.22) |
| United Republic of Tanzania | 20.58(9.2 to 39.38) | 20.15(9.11 to 38.4) | 20.1(8.87 to 38.56) | 20.9(9.27 to 39.18) | -0.11 (-0.13 to -0.09) | 0.02 (-0.02 to 0.07) |
| United States of America | 78.14(54.8 to 97.15) | 84.39(66.36 to 98.45) | 86.79(71.04 to 99.53) | 86.03(69.02 to 99.41) | 0.53 (0.47 to 0.59) | 0.32 (0.26 to 0.39) |
| United States Virgin Islands | 15.9(6.74 to 31.9) | 17.71(7.81 to 34.6) | 18.32(8.23 to 36.78) | 17.81(8.12 to 35.64) | 0.7 (0.61 to 0.79) | 0.38 (0.28 to 0.49) |
| Uruguay | 57.58(35.15 to 80.33) | 60.74(37.2 to 83.11) | 63.19(38.69 to 86.49) | 64.87(40.64 to 87.78) | 0.47 (0.45 to 0.48) | 0.41 (0.39 to 0.43) |
| Uzbekistan | 44.41(24.26 to 69.76) | 42.57(23.99 to 66.07) | 42.29(22.79 to 65.7) | 44.77(24.75 to 69.75) | -0.23 (-0.27 to -0.18) | -0.01 (-0.08 to 0.07) |
| Vanuatu | 9.55(3.4 to 23.34) | 10.33(3.59 to 24.2) | 10.7(3.44 to 25.51) | 10.72(3.5 to 25.39) | 0.57 (0.52 to 0.62) | 0.39 (0.33 to 0.45) |
| Venezuela (Bolivarian Republic of) | 18.04(8.07 to 36.69) | 18.62(8.63 to 38.06) | 19.33(8.76 to 39.14) | 20.59(9.29 to 40.27) | 0.35 (0.34 to 0.36) | 0.43 (0.4 to 0.46) |
| Viet Nam | 4(1.43 to 13.46) | 4.18(1.61 to 11.85) | 4.44(1.67 to 13.21) | 5.29(1.98 to 14.82) | 0.54 (0.52 to 0.56) | 0.85 (0.74 to 0.96) |
| Yemen | 8.66(2.48 to 24.25) | 8.88(2.46 to 24.08) | 8.94(2.34 to 24.61) | 8.8(2.44 to 24.6) | 0.15 (0.13 to 0.17) | 0.06 (0.03 to 0.09) |
| Zambia | 19.04(8.64 to 38.51) | 18.5(8.33 to 37.16) | 18.42(8.22 to 36.44) | 19.12(8.46 to 37.69) | -0.16 (-0.19 to -0.13) | -0.01 (-0.06 to 0.04) |
| Zimbabwe | 14.11(6.09 to 29.32) | 14.19(6.26 to 29.04) | 14.28(6.29 to 29.51) | 14.52(6.43 to 30.22) | 0.06 (0.06 to 0.06) | 0.09 (0.08 to 0.1) |

SEV, summary exposure value; EAPC, estimated annual percentage change.
