# Supplementary material for: Global, regional, and national burden of diet high in processed meat from 1990 to 2019: a systematic analysis from the global burden of disease study 2019
Source: Front Nutr. 2024 Feb 13;11:1354287. doi: 10.3389/fnut.2024.1354287 (PMC10896824; doi:10.3389/fnut.2024.1354287)
Supplement: Supplementary file 8 [file Table_4.docx]

Table 4S. Age-standardized mortality of diet high in processed meat for both sexes combined in 1990,2000,2010, and 2019, and EAPC of ASMR from 1990 to 2010 and 1990 to 2019 in 204 countries and territories

| Location | ASMR 1990 | ASMR 2000 | ASMR 2010 | ASMR 2019 | EAPC 1990-2010 | EAPC 1990-2019 |
| --- | --- | --- | --- | --- | --- | --- |
| Afghanistan | 3.31(1.42 to 7.13) | 3.11(1.47 to 6.33) | 2.89(1.35 to 5.73) | 2.84(1.31 to 5.66) | -0.55 (-0.61 to -0.49) | -0.51 (-0.55 to -0.47) |
| Albania | 10.92(2.28 to 18.76) | 10.27(2.31 to 17.36) | 10.67(2.34 to 18.25) | 10.36(2.27 to 18.8) | 0.5 (0.06 to 0.95) | 0.17 (-0.06 to 0.4) |
| Algeria | 2.97(1.27 to 6.45) | 2.48(1 to 5.58) | 2.27(0.94 to 5.09) | 2.15(0.91 to 4.85) | -1.32 (-1.42 to -1.21) | -1.04 (-1.13 to -0.95) |
| American Samoa | 3.12(1.5 to 5.12) | 3.52(1.7 to 5.57) | 3.71(1.76 to 5.89) | 3.61(1.74 to 5.81) | 1.07 (0.92 to 1.23) | 0.54 (0.37 to 0.7) |
| Andorra | 6.89(2.95 to 11.61) | 5.61(2.46 to 9.19) | 4.87(2.36 to 7.9) | 4.59(2.27 to 7.37) | -1.87 (-1.97 to -1.77) | -1.48 (-1.6 to -1.35) |
| Angola | 2.62(1.12 to 4.87) | 2.73(1.24 to 5.01) | 2.89(1.38 to 5.31) | 2.89(1.37 to 5.34) | 0.47 (0.39 to 0.54) | 0.39 (0.33 to 0.45) |
| Antigua and Barbuda | 2.49(1.2 to 4.12) | 2.43(1.19 to 3.87) | 2.38(1.14 to 3.75) | 2.29(1.08 to 3.6) | -0.57 (-0.9 to -0.25) | -0.55 (-0.71 to -0.39) |
| Argentina | 4.89(2.01 to 10.12) | 4.3(2.06 to 7.95) | 3.86(1.93 to 6.85) | 4.2(2.18 to 6.96) | -1.27 (-1.41 to -1.14) | -0.64 (-0.85 to -0.43) |
| Armenia | 5.62(1.76 to 13.82) | 6.27(2.4 to 14.18) | 6.12(2.48 to 13.3) | 5.67(2.23 to 12.34) | -0.18 (-0.52 to 0.15) | -0.47 (-0.65 to -0.29) |
| Australia | 9.11(3.17 to 16.72) | 6.35(2.64 to 11.15) | 4.97(2.41 to 7.96) | 4.37(2.18 to 6.86) | -2.97 (-3.12 to -2.82) | -2.68 (-2.83 to -2.53) |
| Austria | 8.59(3.12 to 15.49) | 8.58(3.06 to 15.27) | 7.12(3 to 11.65) | 5.6(2.46 to 9.43) | -0.76 (-1.03 to -0.49) | -1.66 (-1.93 to -1.38) |
| Azerbaijan | 8.64(2.06 to 20.19) | 10.46(2.87 to 24.25) | 12.81(3.65 to 27.93) | 13.04(3.68 to 29.37) | 1.34 (1.01 to 1.67) | 1.43 (1.24 to 1.62) |
| Bahamas | 2.72(1.26 to 4.71) | 2.49(1.2 to 4.29) | 2.08(1 to 3.49) | 2.02(0.94 to 3.42) | -1.55 (-1.8 to -1.3) | -1.29 (-1.44 to -1.14) |
| Bahrain | 5.31(2.34 to 10.61) | 4.83(2.2 to 8.66) | 5.23(2.47 to 8.29) | 4.42(2.12 to 6.93) | 0.09 (-0.39 to 0.58) | -0.51 (-0.81 to -0.21) |
| Bangladesh | 3.65(1.65 to 7.2) | 4(1.88 to 7.3) | 4.64(2.14 to 8.79) | 4.23(2.03 to 8.5) | 2.13 (1.76 to 2.49) | 0.74 (0.33 to 1.16) |
| Barbados | 3.52(1.73 to 5.7) | 3.16(1.62 to 4.82) | 2.72(1.44 to 4.02) | 2.75(1.3 to 4.16) | -1.18 (-1.37 to -0.99) | -1.18 (-1.31 to -1.04) |
| Belarus | 18.17(3.23 to 34.19) | 20.12(3.33 to 38.88) | 18.82(2.97 to 37.21) | 15.7(2.39 to 31.72) | -0.19 (-0.63 to 0.25) | -0.93 (-1.23 to -0.63) |
| Belgium | 10.2(3.7 to 16.75) | 8.1(3.2 to 12.91) | 5.92(2.6 to 9.06) | 4.99(2.26 to 7.63) | -2.54 (-2.68 to -2.4) | -2.69 (-2.79 to -2.58) |
| Belize | 2.36(1.08 to 4.25) | 3.44(1.6 to 5.88) | 2.82(1.42 to 4.47) | 2.57(1.26 to 4.14) | 1.47 (0.71 to 2.23) | 0.14 (-0.37 to 0.65) |
| Benin | 3.36(1.46 to 6.69) | 4.01(1.92 to 7.44) | 4.38(2.19 to 8.11) | 4.19(2.09 to 7.71) | 1.42 (1.29 to 1.55) | 0.89 (0.71 to 1.06) |
| Bermuda | 3.26(1.45 to 6.55) | 2.07(0.95 to 3.85) | 1.49(0.73 to 2.62) | 1.34(0.65 to 2.38) | -3.98 (-4.2 to -3.76) | -3.1 (-3.38 to -2.82) |
| Bhutan | 3.67(1.37 to 8.06) | 4.18(1.66 to 8.72) | 4.86(2.1 to 9.57) | 5.37(2.47 to 10.09) | 1.41 (1.36 to 1.46) | 1.38 (1.35 to 1.41) |
| Bolivia (Plurinational State of) | 1.29(0.82 to 2.03) | 1.22(0.77 to 1.83) | 1.22(0.76 to 1.86) | 1.32(0.76 to 2.02) | -0.54 (-0.69 to -0.39) | 0.02 (-0.14 to 0.19) |
| Bosnia and Herzegovina | 7.11(2.31 to 14.84) | 6.97(2.55 to 13.61) | 8.7(4.47 to 14.77) | 8.82(4.28 to 15.51) | 1.24 (0.78 to 1.69) | 0.94 (0.7 to 1.17) |
| Botswana | 2.71(1.2 to 4.69) | 4(1.77 to 6.97) | 3.97(1.86 to 6.65) | 3.93(1.8 to 6.59) | 1.94 (1.38 to 2.5) | 1.05 (0.69 to 1.41) |
| Brazil | 2.64(1.17 to 5.21) | 2.25(1.03 to 4.18) | 2.41(1.17 to 4.35) | 2.36(1.23 to 4.09) | -0.51 (-0.78 to -0.24) | -0.08 (-0.26 to 0.1) |
| Brunei Darussalam | 11.14(6.7 to 17.21) | 9.64(5.73 to 14.5) | 9.48(5.82 to 14.08) | 8(4.67 to 12.19) | -0.18 (-0.6 to 0.25) | -0.65 (-0.9 to -0.4) |
| Bulgaria | 12.77(3.48 to 27.11) | 11.73(3.2 to 26.92) | 11.1(3.57 to 22.05) | 11.69(3.74 to 22.43) | -1.05 (-1.35 to -0.75) | -0.61 (-0.82 to -0.4) |
| Burkina Faso | 3.32(1.54 to 6.2) | 3.84(1.8 to 7.23) | 4.13(1.94 to 7.92) | 4.33(2.06 to 8.33) | 1.29 (1.17 to 1.42) | 1.02 (0.92 to 1.12) |
| Burundi | 3.09(1.35 to 5.81) | 2.97(1.28 to 5.39) | 2.64(1.09 to 5.03) | 2.48(1.04 to 4.74) | -0.97 (-1.12 to -0.82) | -0.97 (-1.04 to -0.89) |
| Cabo Verde | 2.28(0.75 to 5.51) | 2.91(1.29 to 6.02) | 2.98(1.42 to 5.62) | 4.13(1.95 to 7.77) | 1.46 (1.09 to 1.83) | 1.55 (1.31 to 1.8) |
| Cambodia | 1(0.69 to 1.51) | 1.01(0.69 to 1.54) | 1.07(0.71 to 1.65) | 1.13(0.73 to 1.77) | 0.25 (0.2 to 0.3) | 0.44 (0.39 to 0.5) |
| Cameroon | 3.91(1.91 to 6.93) | 4.91(2.56 to 8.53) | 5.54(2.98 to 9.28) | 5.29(2.84 to 8.97) | 2.06 (1.87 to 2.25) | 1.26 (1.01 to 1.51) |
| Canada | 7.87(2.83 to 14.12) | 6.97(3.05 to 11.64) | 4.9(2.38 to 7.87) | 4.36(2.09 to 7.03) | -2.22 (-2.52 to -1.92) | -2.55 (-2.77 to -2.34) |
| Central African Republic | 3.36(1.42 to 6.35) | 3.44(1.44 to 6.68) | 3.6(1.6 to 6.84) | 3.35(1.4 to 6.48) | 0.46 (0.39 to 0.53) | 0.17 (0.07 to 0.28) |
| Chad | 2.74(1.23 to 5.58) | 3.46(1.6 to 6.62) | 3.86(1.81 to 7.3) | 3.74(1.76 to 7.25) | 1.85 (1.65 to 2.05) | 1.15 (0.93 to 1.37) |
| Chile | 8.04(3.12 to 13.66) | 6.76(3.23 to 10.33) | 6.75(3.6 to 9.74) | 6.23(3.5 to 8.76) | -0.67 (-0.86 to -0.49) | -0.64 (-0.73 to -0.55) |
| China | 0.79(0.47 to 1.35) | 0.83(0.48 to 1.45) | 1.27(0.6 to 2.54) | 1.33(0.57 to 2.76) | 2.7 (2.2 to 3.21) | 2.56 (2.28 to 2.84) |
| Colombia | 1.8(0.74 to 3.7) | 1.57(0.67 to 3) | 1.38(0.58 to 2.77) | 1.3(0.53 to 2.67) | -1.53 (-1.72 to -1.34) | -1.43 (-1.56 to -1.3) |
| Comoros | 2.9(1.29 to 5.71) | 2.87(1.33 to 5.22) | 2.66(1.23 to 4.97) | 2.71(1.24 to 5.02) | -0.56 (-0.71 to -0.41) | -0.34 (-0.44 to -0.25) |
| Congo | 4.1(1.83 to 7.7) | 3.93(1.81 to 7.43) | 3.63(1.67 to 6.99) | 3.57(1.65 to 6.72) | -0.65 (-0.81 to -0.49) | -0.55 (-0.64 to -0.47) |
| Cook Islands | 3.73(1.73 to 5.58) | 3.83(1.86 to 5.58) | 3.66(1.73 to 5.35) | 3.68(1.64 to 5.64) | 0.02 (-0.12 to 0.15) | -0.15 (-0.23 to -0.07) |
| Costa Rica | 1.84(0.76 to 3.93) | 1.54(0.62 to 3.21) | 1.27(0.51 to 2.63) | 1.3(0.54 to 2.63) | -2.44 (-2.76 to -2.11) | -1.66 (-1.98 to -1.35) |
| Croatia | 2.77(0.99 to 6.5) | 2.09(0.81 to 4.58) | 2(0.77 to 4.39) | 1.83(0.68 to 4.06) | -1.55 (-1.83 to -1.27) | -1.21 (-1.38 to -1.05) |
| Cuba | 2.64(1.07 to 5.61) | 1.88(0.72 to 4.16) | 1.66(0.65 to 3.62) | 1.62(0.61 to 3.57) | -2.76 (-3.13 to -2.39) | -1.86 (-2.19 to -1.52) |
| Cyprus | 14.53(7.37 to 24.43) | 11.14(5.79 to 18.95) | 7.59(4.17 to 12.57) | 6.23(3.28 to 10.33) | -3.62 (-3.86 to -3.38) | -3.43 (-3.58 to -3.27) |
| Czechia | 11.4(3.31 to 23.45) | 8.03(2.43 to 16.56) | 6.9(2.55 to 13.22) | 6.34(2.54 to 12.01) | -2.38 (-2.61 to -2.16) | -1.88 (-2.06 to -1.71) |
| C么te d'Ivoire | 4.74(2.21 to 9.37) | 5.66(2.79 to 10.3) | 5.41(2.65 to 9.64) | 4.99(2.49 to 8.79) | 0.79 (0.5 to 1.09) | 0.1 (-0.13 to 0.34) |
| Democratic People's Republic of Korea | 1.18(0.53 to 2.47) | 1.3(0.59 to 2.69) | 1.47(0.62 to 3.16) | 1.34(0.55 to 2.86) | 1.21 (1.14 to 1.28) | 0.63 (0.44 to 0.81) |
| Democratic Republic of the Congo | 3.2(1.46 to 6.18) | 2.7(1.22 to 5.27) | 2.43(1.06 to 4.77) | 2.43(1.02 to 4.8) | -1.43 (-1.52 to -1.34) | -1.03 (-1.16 to -0.91) |
| Denmark | 13.25(3.95 to 22.79) | 10.07(4.13 to 15.83) | 7.06(3.72 to 10.41) | 5.86(3.12 to 8.54) | -3.18 (-3.36 to -3.01) | -3.25 (-3.4 to -3.1) |
| Djibouti | 2.09(0.92 to 3.98) | 2.35(1.02 to 4.38) | 2.82(1.29 to 5.33) | 2.9(1.39 to 5.3) | 1.58 (1.48 to 1.68) | 1.28 (1.17 to 1.39) |
| Dominica | 3.33(1.56 to 5.8) | 3.29(1.54 to 5.33) | 2.95(1.42 to 4.65) | 2.85(1.35 to 4.53) | -0.72 (-0.85 to -0.59) | -0.69 (-0.76 to -0.63) |
| Dominican Republic | 1.76(0.73 to 3.67) | 1.68(0.7 to 3.36) | 2.24(0.9 to 4.68) | 2.65(1.04 to 5.71) | 1.74 (1.28 to 2.21) | 2.18 (1.91 to 2.46) |
| Ecuador | 1.3(0.72 to 2.24) | 1.32(0.74 to 2.14) | 1.58(0.91 to 2.5) | 1.58(0.86 to 2.58) | 0.88 (0.54 to 1.23) | 0.8 (0.62 to 0.97) |
| Egypt | 3.59(1.33 to 8.26) | 3.45(1.22 to 7.9) | 4.38(1.53 to 10.2) | 4.59(1.56 to 11.14) | 1.14 (0.88 to 1.4) | 1.23 (1.09 to 1.38) |
| El Salvador | 1.66(0.64 to 3.62) | 1.92(0.87 to 3.67) | 2.1(1 to 3.74) | 2.29(0.97 to 4.2) | 1.29 (1.07 to 1.51) | 1.11 (0.98 to 1.25) |
| Equatorial Guinea | 3.05(1.32 to 6.11) | 3.09(1.41 to 5.8) | 3.78(1.95 to 6.52) | 3.95(1.99 to 6.68) | 1.27 (0.99 to 1.54) | 1.28 (1.14 to 1.42) |
| Eritrea | 2.45(1.03 to 4.54) | 2.92(1.33 to 5.29) | 3.03(1.4 to 5.6) | 3.06(1.38 to 5.5) | 1.25 (1.04 to 1.46) | 0.7 (0.52 to 0.88) |
| Estonia | 27.92(5.46 to 47.73) | 24.7(5.36 to 41.68) | 15.36(4.07 to 25.77) | 12.17(3.54 to 21.04) | -2.78 (-3.3 to -2.27) | -3.6 (-3.95 to -3.25) |
| Eswatini | 3.58(1.65 to 5.63) | 4.79(2.27 to 7.45) | 5.96(2.7 to 9.44) | 5.25(2.37 to 8.2) | 3.55 (3.06 to 4.05) | 1.79 (1.25 to 2.33) |
| Ethiopia | 2.93(1.42 to 4.98) | 2.44(1.18 to 4.17) | 2.06(0.99 to 3.57) | 2.09(1.01 to 3.7) | -1.8 (-1.85 to -1.75) | -1.34 (-1.49 to -1.2) |
| Fiji | 5.26(2.51 to 8.71) | 7.9(3.77 to 11.86) | 7.8(3.66 to 11.56) | 7.64(3.5 to 11.99) | 1.82 (1.37 to 2.28) | 0.95 (0.63 to 1.28) |
| Finland | 11.16(2.28 to 21.23) | 9.56(2.32 to 17.02) | 7.36(1.98 to 12.72) | 5.51(1.57 to 9.92) | -1.76 (-1.97 to -1.56) | -2.48 (-2.7 to -2.26) |
| France | 7.06(2.98 to 11.38) | 6.41(3.06 to 9.64) | 5.02(2.58 to 7.35) | 4.21(2.25 to 6.07) | -1.39 (-1.65 to -1.13) | -1.91 (-2.1 to -1.72) |
| Gabon | 4.14(2.02 to 7.16) | 4.66(2.29 to 7.68) | 4.63(2.33 to 7.83) | 4.4(2.19 to 7.32) | 0.83 (0.65 to 1.01) | 0.18 (-0.02 to 0.38) |
| Gambia | 3.62(1.51 to 7.54) | 4.09(1.93 to 7.99) | 4.63(2.21 to 8.91) | 4.94(2.31 to 9.77) | 1.49 (1.29 to 1.68) | 1.09 (0.93 to 1.24) |
| Georgia | 10.89(2.31 to 25.65) | 9.1(2.15 to 21.55) | 7.85(2.52 to 16.79) | 7.37(2.7 to 15.38) | -1.75 (-2.07 to -1.43) | -1.53 (-1.73 to -1.34) |
| Germany | 16.21(5.31 to 25.93) | 12.09(4.73 to 18.7) | 8.73(3.8 to 13.17) | 7.65(3.32 to 11.58) | -3.03 (-3.14 to -2.93) | -2.86 (-2.99 to -2.74) |
| Ghana | 3.61(1.64 to 7.27) | 4.55(2.28 to 8.43) | 5.89(3.09 to 10.09) | 5.74(2.96 to 10.04) | 2.83 (2.67 to 2.99) | 1.91 (1.63 to 2.19) |
| Greece | 5.16(1.62 to 10.56) | 4.58(1.38 to 9.66) | 4.57(1.45 to 8.93) | 4.11(1.4 to 7.84) | -0.34 (-0.54 to -0.15) | -0.68 (-0.83 to -0.53) |
| Greenland | 15.96(5.54 to 25.86) | 15.12(5.91 to 23.6) | 11(4.7 to 16.96) | 9.52(4.21 to 14.73) | -2.19 (-2.59 to -1.79) | -2.25 (-2.45 to -2.05) |
| Grenada | 3.41(1.61 to 5.93) | 3.02(1.46 to 4.8) | 3.21(1.61 to 4.92) | 2.97(1.48 to 4.71) | -0.4 (-0.64 to -0.16) | -0.41 (-0.54 to -0.28) |
| Guam | 2.7(1.22 to 5.12) | 2.23(0.97 to 4.45) | 2.11(0.88 to 4.4) | 2.3(0.93 to 4.96) | -1.34 (-1.7 to -0.97) | -0.56 (-0.84 to -0.27) |
| Guatemala | 1.91(0.74 to 4.28) | 2.51(1.15 to 4.49) | 3(1.44 to 4.83) | 3.03(1.4 to 4.98) | 1.72 (1.43 to 2) | 1.46 (1.28 to 1.64) |
| Guinea | 3.44(1.61 to 6.61) | 3.84(1.92 to 6.89) | 4.57(2.25 to 8.43) | 4.59(2.25 to 8.43) | 1.62 (1.53 to 1.72) | 1.33 (1.21 to 1.45) |
| Guinea-Bissau | 4.87(2.19 to 9.69) | 5.36(2.53 to 10.16) | 5.75(2.8 to 10.77) | 5.5(2.61 to 10.48) | 0.95 (0.88 to 1.02) | 0.51 (0.38 to 0.64) |
| Guyana | 4.84(2.1 to 9.01) | 5.82(2.74 to 9.74) | 5.19(2.46 to 8.99) | 4.95(2.26 to 8.7) | 0.7 (0.27 to 1.13) | -0.12 (-0.42 to 0.18) |
| Haiti | 3.75(1.7 to 6.85) | 3.17(1.42 to 5.67) | 3.18(1.36 to 5.78) | 3.08(1.29 to 5.68) | -0.71 (-0.93 to -0.5) | -0.44 (-0.57 to -0.32) |
| Honduras | 1.46(0.55 to 3.31) | 1.71(0.64 to 3.76) | 1.9(0.72 to 4.24) | 2.06(0.78 to 4.48) | 1.16 (0.89 to 1.43) | 1.34 (1.17 to 1.52) |
| Hungary | 10.05(2.97 to 21.2) | 8.48(2.66 to 17.89) | 9.28(3.31 to 17.49) | 8.46(3.03 to 15.88) | -0.23 (-0.6 to 0.14) | -0.32 (-0.5 to -0.13) |
| Iceland | 9.9(2.33 to 17.53) | 7.01(2.03 to 12.29) | 5.07(1.66 to 8.8) | 4.28(1.55 to 7.45) | -3.39 (-3.48 to -3.3) | -3.14 (-3.26 to -3.03) |
| India | 2(1.38 to 3.02) | 2.19(1.52 to 3.25) | 2.43(1.69 to 3.52) | 2.54(1.76 to 3.64) | 0.99 (0.78 to 1.21) | 1.03 (0.9 to 1.15) |
| Indonesia | 0.73(0.62 to 0.87) | 0.79(0.68 to 0.96) | 1.04(0.85 to 1.32) | 1.22(0.91 to 1.58) | 1.78 (1.53 to 2.03) | 2.13 (1.97 to 2.29) |
| Iran (Islamic Republic of) | 2.38(1.06 to 5.18) | 2.3(1.02 to 5.05) | 1.79(0.85 to 3.63) | 1.8(0.89 to 3.51) | -1.43 (-1.71 to -1.15) | -1.35 (-1.54 to -1.16) |
| Iraq | 3.3(1.47 to 6.64) | 2.85(1.33 to 5.55) | 2.7(1.28 to 5.17) | 2.68(1.3 to 5.06) | -0.95 (-1.08 to -0.81) | -0.8 (-0.9 to -0.71) |
| Ireland | 11.03(3.29 to 20.9) | 9.29(2.97 to 16.54) | 5.98(2.31 to 9.92) | 4.89(1.95 to 8.3) | -2.74 (-3.05 to -2.44) | -3.15 (-3.35 to -2.95) |
| Israel | 5.74(2.2 to 11.92) | 6.01(3.33 to 9.57) | 3.95(2.35 to 5.97) | 3.62(2.18 to 5.43) | -1.75 (-2.29 to -1.21) | -2.3 (-2.62 to -1.98) |
| Italy | 8.53(3.89 to 13.72) | 7.01(3.43 to 10.87) | 5.6(2.94 to 8.29) | 4.93(2.61 to 7.38) | -2.02 (-2.09 to -1.94) | -2.02 (-2.08 to -1.95) |
| Jamaica | 2.7(1.31 to 4.15) | 3.3(1.66 to 4.73) | 2.91(1.49 to 4.2) | 3.08(1.47 to 4.66) | 0.53 (0.04 to 1.03) | 0.21 (-0.06 to 0.48) |
| Japan | 3.69(1.69 to 6.28) | 3.16(1.46 to 4.98) | 2.69(1.28 to 4.13) | 2.09(1.03 to 3.27) | -1.57 (-1.65 to -1.49) | -1.92 (-2.04 to -1.79) |
| Jordan | 2.97(1.4 to 5.29) | 2.95(1.4 to 5.2) | 2.2(1.09 to 3.86) | 1.91(0.89 to 3.36) | -1.19 (-1.68 to -0.69) | -1.93 (-2.25 to -1.61) |
| Kazakhstan | 9.94(2.13 to 21.81) | 12.86(2.65 to 29.47) | 11.13(2.5 to 24.38) | 8.82(2.75 to 18.02) | 0.02 (-0.7 to 0.76) | -1.17 (-1.64 to -0.69) |
| Kenya | 1.76(0.91 to 3.02) | 1.97(1.05 to 3.32) | 2.34(1.23 to 4.09) | 2.33(1.23 to 4.09) | 1.75 (1.54 to 1.96) | 1.2 (1 to 1.39) |
| Kiribati | 4.79(2.26 to 8.2) | 6.12(2.91 to 9.91) | 6.31(2.98 to 10.2) | 6.21(2.96 to 10.19) | 1.54 (1.27 to 1.81) | 0.72 (0.46 to 0.97) |
| Kuwait | 2.19(0.93 to 4.46) | 2.31(0.99 to 4.56) | 1.74(0.73 to 3.58) | 1.47(0.61 to 2.99) | 0.13 (-0.52 to 0.78) | -1.34 (-1.85 to -0.84) |
| Kyrgyzstan | 6.95(1.63 to 15.63) | 7.98(1.78 to 18.9) | 9.57(1.82 to 23.21) | 8.09(1.68 to 18.87) | 1.69 (1.29 to 2.08) | 0.74 (0.39 to 1.08) |
| Lao People's Democratic Republic | 1.32(0.88 to 2.11) | 1.39(0.95 to 2.1) | 1.43(0.93 to 2.25) | 1.46(0.94 to 2.28) | 0.47 (0.43 to 0.52) | 0.28 (0.22 to 0.34) |
| Latvia | 28.12(5.65 to 48.73) | 25.19(5.5 to 43.1) | 22.39(5.67 to 37.67) | 17.76(4.46 to 31.06) | -1.38 (-1.93 to -0.83) | -1.98 (-2.29 to -1.67) |
| Lebanon | 3.11(1.24 to 7.01) | 2.47(1.02 to 5.51) | 2.42(1.01 to 5.27) | 2.26(0.94 to 4.97) | -1.36 (-1.64 to -1.08) | -0.87 (-1.06 to -0.68) |
| Lesotho | 2.36(1.05 to 3.84) | 3.18(1.38 to 5.12) | 4.97(2.26 to 8.25) | 5.24(2.35 to 8.6) | 4.59 (4.12 to 5.07) | 3.6 (3.21 to 4) |
| Liberia | 4.04(1.85 to 7.93) | 3.69(1.73 to 7.13) | 4.2(2.04 to 8.03) | 3.95(1.86 to 7.46) | 0.13 (-0.15 to 0.41) | 0.25 (0.08 to 0.41) |
| Libya | 1.92(0.75 to 4.29) | 1.8(0.74 to 3.92) | 1.82(0.76 to 3.82) | 1.78(0.75 to 3.8) | -0.12 (-0.45 to 0.21) | -0.03 (-0.19 to 0.13) |
| Lithuania | 28.79(5.54 to 50.99) | 24.82(5.1 to 43.83) | 23.89(5.03 to 41.64) | 19.02(4.46 to 34.09) | -1.1 (-1.54 to -0.65) | -1.52 (-1.77 to -1.27) |
| Luxembourg | 13.45(4.72 to 21.25) | 10.21(3.89 to 15.71) | 7.01(3.03 to 10.65) | 5.21(2.36 to 7.88) | -3.16 (-3.32 to -3.01) | -3.49 (-3.62 to -3.36) |
| Madagascar | 2.63(1.1 to 5.29) | 2.66(1.12 to 5.34) | 2.7(1.09 to 5.78) | 2.61(1.06 to 5.41) | 0.21 (0.09 to 0.33) | -0.02 (-0.1 to 0.07) |
| Malawi | 2.56(1.12 to 4.62) | 2.99(1.38 to 5.42) | 2.85(1.29 to 5.2) | 2.61(1.26 to 4.79) | 0.66 (0.38 to 0.95) | 0 (-0.22 to 0.23) |
| Malaysia | 2.21(0.93 to 4.39) | 2.45(0.99 to 5.06) | 2.17(0.8 to 4.92) | 2.25(0.81 to 5.09) | 0.05 (-0.32 to 0.43) | -0.43 (-0.69 to -0.18) |
| Maldives | 1.77(1.21 to 2.73) | 1.43(1 to 2.16) | 1.04(0.69 to 1.65) | 0.97(0.6 to 1.56) | -3.19 (-3.45 to -2.92) | -2.64 (-2.87 to -2.41) |
| Mali | 3.71(1.73 to 7.15) | 4.11(2.05 to 7.44) | 4.56(2.34 to 8.08) | 4.52(2.34 to 8.16) | 1.06 (1.02 to 1.11) | 0.77 (0.68 to 0.86) |
| Malta | 17.42(6.14 to 27.25) | 13.31(4.95 to 20.65) | 9.92(3.95 to 15.17) | 8.13(3.33 to 12.57) | -2.54 (-2.68 to -2.41) | -2.62 (-2.7 to -2.55) |
| Marshall Islands | 3.42(1.72 to 6.06) | 3.95(1.87 to 6.81) | 4.42(2.06 to 7.84) | 4.42(1.93 to 8) | 1.41 (1.28 to 1.53) | 0.94 (0.8 to 1.09) |
| Mauritania | 4.99(2.29 to 9.68) | 4.76(2.39 to 8.62) | 4.69(2.42 to 8.35) | 4.44(2.25 to 7.83) | -0.29 (-0.35 to -0.23) | -0.33 (-0.38 to -0.28) |
| Mauritius | 2.29(1.37 to 3.85) | 2.19(1.28 to 3.64) | 2.91(1.62 to 4.09) | 2.55(1.34 to 3.82) | 1.66 (1.21 to 2.12) | 0.93 (0.61 to 1.24) |
| Mexico | 3.78(2.12 to 5.69) | 3.49(1.97 to 5.29) | 3.52(2.04 to 5.33) | 3.84(2.13 to 5.84) | -0.51 (-0.68 to -0.34) | -0.05 (-0.21 to 0.11) |
| Micronesia (Federated States of) | 3.63(1.75 to 6.27) | 4.53(2.19 to 7.3) | 5.1(2.5 to 8.35) | 5.45(2.56 to 9.3) | 1.92 (1.76 to 2.08) | 1.41 (1.25 to 1.57) |
| Monaco | 9.17(2.71 to 15.36) | 7.31(2.65 to 12.22) | 6.53(2.72 to 10.58) | 5.93(2.41 to 9.41) | -1.99 (-2.19 to -1.78) | -1.54 (-1.69 to -1.38) |
| Mongolia | 10.11(1.73 to 24.34) | 11.39(1.93 to 28.5) | 8.35(1.56 to 20.33) | 7.89(1.47 to 19.21) | -1.21 (-1.78 to -0.63) | -1.5 (-1.8 to -1.2) |
| Montenegro | 9.05(2.76 to 16.22) | 9.71(3.01 to 17.35) | 10.53(3.44 to 18.4) | 9.48(3.2 to 16.89) | 1.11 (0.95 to 1.27) | 0.44 (0.23 to 0.65) |
| Morocco | 1.81(1.05 to 3.2) | 1.73(0.99 to 3.12) | 2.04(0.88 to 4.31) | 2.38(0.99 to 5.15) | 0.34 (0.01 to 0.67) | 1.12 (0.85 to 1.4) |
| Mozambique | 2.06(0.92 to 3.58) | 2.18(0.99 to 3.79) | 2.85(1.33 to 5.01) | 3.01(1.38 to 5.37) | 1.63 (1.33 to 1.93) | 1.83 (1.63 to 2.02) |
| Myanmar | 1.32(0.89 to 1.91) | 1.32(0.9 to 1.97) | 1.32(0.86 to 1.95) | 1.31(0.86 to 1.92) | -0.02 (-0.05 to 0.02) | -0.06 (-0.08 to -0.03) |
| Namibia | 2.57(1.15 to 4.42) | 3.16(1.47 to 5.32) | 2.66(1.2 to 4.46) | 2.66(1.16 to 4.56) | 0.6 (0.07 to 1.14) | -0.17 (-0.5 to 0.17) |
| Nauru | 5.25(2.31 to 9.53) | 6.21(2.92 to 10.93) | 6.3(2.86 to 11.09) | 6.55(3.01 to 11.64) | 1.01 (0.8 to 1.23) | 0.64 (0.5 to 0.78) |
| Nepal | 2.82(0.91 to 6.54) | 2.93(1.03 to 6.58) | 3.5(1.3 to 7.66) | 4.07(1.62 to 8.57) | 0.94 (0.74 to 1.15) | 1.43 (1.26 to 1.59) |
| Netherlands | 8.74(3.55 to 15.11) | 7.01(3.18 to 11.46) | 4.73(2.45 to 7.25) | 4.29(2.26 to 6.49) | -2.94 (-3.2 to -2.67) | -2.87 (-3.05 to -2.68) |
| New Zealand | 9.46(3.24 to 17.35) | 7.2(2.95 to 12.66) | 5.44(2.38 to 9.02) | 5.08(2.28 to 8.24) | -2.7 (-2.77 to -2.63) | -2.46 (-2.6 to -2.32) |
| Nicaragua | 1.69(0.75 to 3.12) | 2.3(1.06 to 4.09) | 2.8(1.27 to 4.97) | 2.82(1.25 to 5.04) | 2.32 (1.82 to 2.81) | 1.62 (1.3 to 1.93) |
| Niger | 3.52(1.47 to 7.4) | 3.75(1.74 to 7.31) | 3.92(1.91 to 7.2) | 4.04(1.9 to 7.6) | 0.83 (0.68 to 0.98) | 0.6 (0.51 to 0.7) |
| Nigeria | 3.69(1.73 to 7.44) | 4.2(2.03 to 7.85) | 4.28(2.18 to 7.64) | 4.13(2.07 to 7.23) | 0.78 (0.62 to 0.95) | 0.43 (0.29 to 0.56) |
| Niue | 3.5(1.64 to 6.03) | 4.19(1.94 to 7.02) | 4.48(2.14 to 7.45) | 4.46(2.02 to 7.38) | 1.29 (1.13 to 1.44) | 0.77 (0.61 to 0.93) |
| North Macedonia | 8.23(3 to 16.68) | 9.72(3.83 to 18.83) | 10.8(4.92 to 19.55) | 9.93(4.43 to 18.12) | 1.63 (1.44 to 1.81) | 0.69 (0.42 to 0.97) |
| Northern Mariana Islands | 2.45(1.13 to 4.31) | 2.51(1.21 to 4.36) | 2.99(1.41 to 5.13) | 2.81(1.32 to 4.88) | 1.53 (1.21 to 1.85) | 0.77 (0.51 to 1.03) |
| Norway | 15.72(4.4 to 25.7) | 12.18(4.27 to 19.21) | 8.18(3.42 to 12.5) | 6.38(2.92 to 9.61) | -3.29 (-3.47 to -3.11) | -3.37 (-3.48 to -3.27) |
| Oman | 3.95(1.86 to 8.03) | 4.26(1.94 to 8.6) | 4.25(1.86 to 8.26) | 3.58(1.66 to 6.76) | 0.32 (0.19 to 0.46) | -0.11 (-0.28 to 0.06) |
| Pakistan | 4.73(1.83 to 9.6) | 6.74(2.8 to 13.15) | 7.82(3.56 to 14.81) | 7.98(3.72 to 15.22) | 2.49 (2.19 to 2.79) | 1.68 (1.41 to 1.94) |
| Palau | 3.54(1.59 to 6.37) | 4.15(1.89 to 7.16) | 4.47(2.08 to 7.77) | 4.6(2.06 to 7.86) | 1.28 (1.16 to 1.4) | 0.92 (0.81 to 1.04) |
| Palestine | 2.78(1.5 to 5.08) | 2.57(1.41 to 4.6) | 2.23(1.23 to 3.81) | 2.52(1.38 to 4.1) | -1.25 (-1.37 to -1.13) | -0.62 (-0.86 to -0.38) |
| Panama | 1.52(0.67 to 3.02) | 1.4(0.67 to 2.46) | 1.48(0.71 to 2.54) | 1.51(0.7 to 2.58) | -0.3 (-0.48 to -0.11) | 0.08 (-0.07 to 0.22) |
| Papua New Guinea | 2.23(1.14 to 3.67) | 2.61(1.32 to 4.24) | 2.9(1.46 to 4.71) | 3.01(1.49 to 4.86) | 1.39 (1.29 to 1.48) | 1.04 (0.93 to 1.14) |
| Paraguay | 1.73(0.71 to 3.48) | 2(0.94 to 3.47) | 2.39(1.12 to 4.03) | 2.46(1.1 to 4.2) | 1.81 (1.63 to 2) | 1.36 (1.2 to 1.51) |
| Peru | 0.66(0.48 to 0.96) | 0.51(0.37 to 0.71) | 0.55(0.36 to 0.84) | 0.55(0.3 to 0.9) | -1.52 (-2.04 to -1) | -0.34 (-0.75 to 0.07) |
| Philippines | 1.85(0.95 to 3.15) | 2.19(1.02 to 4.04) | 2.8(1.23 to 5.49) | 3.13(1.42 to 6.15) | 2.58 (2.37 to 2.8) | 2.36 (2.22 to 2.5) |
| Poland | 6.37(1.84 to 15.16) | 4.91(1.52 to 11.38) | 4.72(1.77 to 9.6) | 4.18(1.65 to 8.25) | -1.38 (-1.72 to -1.04) | -1.38 (-1.54 to -1.21) |
| Portugal | 4.35(1.95 to 8.31) | 4.17(2.15 to 7.31) | 3.35(1.86 to 5.39) | 2.82(1.57 to 4.56) | -1.14 (-1.45 to -0.84) | -1.84 (-2.08 to -1.59) |
| Puerto Rico | 2.81(1.3 to 4.97) | 2.68(1.29 to 4.31) | 2.3(1.18 to 3.53) | 2.06(0.97 to 3.4) | -0.94 (-1.15 to -0.73) | -1.38 (-1.56 to -1.2) |
| Qatar | 6.28(2.71 to 12.2) | 6.63(2.94 to 11.82) | 6.95(3.29 to 11.41) | 5.44(2.61 to 9.11) | 0.95 (0.43 to 1.46) | -0.18 (-0.57 to 0.22) |
| Republic of Korea | 3.15(1.36 to 6.46) | 3.53(2.18 to 5.24) | 2.8(1.72 to 4.18) | 1.94(1.11 to 3.03) | 0 (-0.54 to 0.53) | -1.77 (-2.31 to -1.22) |
| Republic of Moldova | 19.84(3.61 to 37.74) | 18.2(3.11 to 35.87) | 16.04(3.12 to 31.35) | 11.84(2.45 to 23) | -1.68 (-2.16 to -1.2) | -2.2 (-2.48 to -1.92) |
| Romania | 7.02(1.58 to 16.71) | 6.79(1.51 to 16.23) | 8.66(2.3 to 17.39) | 8.35(2.4 to 15.79) | 0.7 (0.37 to 1.04) | 0.69 (0.53 to 0.86) |
| Russian Federation | 27.03(5.35 to 47.33) | 32.64(6.23 to 57.22) | 23.16(4.81 to 39.11) | 14.88(3.63 to 26.62) | -0.96 (-1.79 to -0.13) | -2.73 (-3.34 to -2.12) |
| Rwanda | 3(1.36 to 5.32) | 2.74(1.26 to 4.82) | 2.2(1.03 to 3.9) | 2.34(1.07 to 4.2) | -2.09 (-2.41 to -1.76) | -1.42 (-1.68 to -1.16) |
| Saint Kitts and Nevis | 4.16(1.85 to 7.63) | 3(1.48 to 5.26) | 2.39(1.21 to 3.89) | 2.5(1.23 to 4.12) | -2.27 (-2.52 to -2.02) | -1.77 (-1.99 to -1.55) |
| Saint Lucia | 3.65(1.75 to 5.88) | 3.01(1.47 to 4.52) | 2.1(1.05 to 3.13) | 2.36(1.13 to 3.65) | -3.03 (-3.33 to -2.72) | -2.33 (-2.69 to -1.97) |
| Saint Vincent and the Grenadines | 3.91(1.76 to 6.67) | 3.91(1.89 to 6.18) | 3.63(1.73 to 5.82) | 3.75(1.81 to 6.04) | -0.92 (-1.14 to -0.7) | -0.34 (-0.53 to -0.15) |
| Samoa | 3.11(1.51 to 5.57) | 3.56(1.64 to 6.13) | 3.87(1.71 to 6.74) | 3.77(1.68 to 6.7) | 1.29 (1.18 to 1.41) | 0.72 (0.56 to 0.89) |
| San Marino | 6.18(2.76 to 9.89) | 5.05(2.43 to 7.83) | 4.69(2.3 to 7.73) | 4.36(2.04 to 7.26) | -1.32 (-1.49 to -1.15) | -1.11 (-1.21 to -1.01) |
| Sao Tome and Principe | 2.74(0.98 to 6.18) | 3.36(1.22 to 7.47) | 3.59(1.29 to 7.89) | 3.79(1.39 to 8.22) | 1.47 (1.26 to 1.69) | 1.09 (0.95 to 1.24) |
| Saudi Arabia | 2.18(0.91 to 4.57) | 2.45(1.03 to 5.06) | 2.41(0.94 to 5.32) | 2.16(0.82 to 4.85) | 0.41 (0.26 to 0.56) | -0.12 (-0.28 to 0.05) |
| Senegal | 3.88(1.83 to 7.63) | 3.77(1.77 to 7.16) | 4.27(2.09 to 7.87) | 4.56(2.29 to 8.19) | 0.79 (0.55 to 1.04) | 0.72 (0.6 to 0.84) |
| Serbia | 7.45(2.61 to 15.98) | 7.14(2.45 to 15.55) | 6.66(2.62 to 13.58) | 6.36(2.48 to 12.72) | -0.61 (-0.84 to -0.37) | -0.7 (-0.83 to -0.58) |
| Seychelles | 1.09(0.75 to 1.71) | 1.13(0.75 to 1.77) | 1.15(0.77 to 1.8) | 1.19(0.76 to 1.87) | 0.37 (0.22 to 0.53) | 0.13 (0.02 to 0.24) |
| Sierra Leone | 3.27(1.32 to 7.16) | 3.45(1.52 to 7.18) | 4.01(1.82 to 8.08) | 3.88(1.66 to 8.02) | 1.33 (1.15 to 1.5) | 0.9 (0.74 to 1.06) |
| Singapore | 2.75(1.08 to 5.91) | 2.15(0.86 to 4.57) | 1.57(0.55 to 3.42) | 1.2(0.37 to 2.64) | -2.2 (-2.43 to -1.97) | -2.92 (-3.16 to -2.69) |
| Slovakia | 11.05(2.98 to 24.08) | 9.1(2.54 to 19.79) | 9.4(2.88 to 18.62) | 8.89(2.81 to 17.15) | -0.47 (-0.79 to -0.14) | -0.44 (-0.6 to -0.28) |
| Slovenia | 10.91(3.77 to 18.91) | 11.14(5.04 to 16.77) | 7.15(3.26 to 10.87) | 5.79(2.59 to 9.27) | -2.45 (-3.08 to -1.82) | -3.03 (-3.38 to -2.67) |
| Solomon Islands | 3.98(2.04 to 7.44) | 4.65(2.31 to 8.45) | 5.03(2.49 to 9.57) | 5.55(2.74 to 10.09) | 1.32 (1.18 to 1.46) | 1.19 (1.11 to 1.28) |
| Somalia | 2.78(1.2 to 5.1) | 2.57(1.07 to 4.89) | 2.83(1.21 to 5.45) | 2.75(1.18 to 5.35) | 0.3 (0.08 to 0.52) | 0.28 (0.17 to 0.39) |
| South Africa | 2.29(1.13 to 3.76) | 3.53(1.75 to 5.64) | 4.13(2.16 to 6.27) | 3.54(1.91 to 5.29) | 3.13 (2.65 to 3.62) | 1.96 (1.52 to 2.4) |
| South Sudan | 2.7(1.25 to 4.92) | 2.55(1.21 to 4.61) | 2.67(1.28 to 4.96) | 2.6(1.21 to 4.76) | -0.04 (-0.2 to 0.11) | 0 (-0.08 to 0.08) |
| Spain | 10.38(4.61 to 15.79) | 7.47(3.36 to 11.43) | 5.37(2.76 to 8.05) | 4.67(2.47 to 6.88) | -3.14 (-3.21 to -3.07) | -2.95 (-3.06 to -2.83) |
| Sri Lanka | 1.26(0.82 to 2.08) | 1.38(0.88 to 2.15) | 1.66(1.04 to 2.47) | 1.52(0.82 to 2.39) | 1.69 (1.43 to 1.95) | 1.27 (1.03 to 1.51) |
| Sudan | 2.6(1.09 to 5.75) | 2.36(0.97 to 5.3) | 2.19(0.9 to 4.99) | 2.18(0.92 to 4.89) | -0.93 (-0.97 to -0.89) | -0.71 (-0.78 to -0.63) |
| Suriname | 2.8(1.17 to 5.58) | 2.39(1.06 to 4.47) | 2.36(1.09 to 4.19) | 2.6(1.23 to 4.6) | -1.04 (-1.66 to -0.41) | -0.24 (-0.63 to 0.15) |
| Sweden | 13.53(3.72 to 22.52) | 10.49(3.56 to 16.9) | 8.05(3.18 to 12.44) | 6.87(2.86 to 10.53) | -2.49 (-2.55 to -2.44) | -2.48 (-2.55 to -2.41) |
| Switzerland | 8.05(2.91 to 13.88) | 6.64(2.7 to 11.14) | 4.62(1.97 to 7.53) | 3.93(1.69 to 6.46) | -2.77 (-2.99 to -2.55) | -2.84 (-2.98 to -2.7) |
| Syrian Arab Republic | 3.29(1.2 to 7.7) | 3.43(1.31 to 7.67) | 3.03(1.15 to 7.17) | 2.9(1.09 to 6.72) | -0.91 (-1.22 to -0.61) | -0.79 (-0.94 to -0.64) |
| Taiwan (Province of China) | 2.11(1.01 to 3.6) | 2.51(1.35 to 3.78) | 2.14(1.16 to 3.31) | 2.24(1.19 to 3.51) | 0.13 (-0.31 to 0.56) | -0.28 (-0.52 to -0.03) |
| Tajikistan | 5.5(1.54 to 13.15) | 6.49(1.77 to 15.86) | 8.58(2.58 to 20.15) | 9.47(3.01 to 21.81) | 1.73 (1.4 to 2.07) | 2.14 (1.91 to 2.37) |
| Thailand | 0.67(0.52 to 0.9) | 0.69(0.51 to 0.95) | 0.56(0.38 to 0.84) | 0.6(0.34 to 0.97) | -1.27 (-1.54 to -1) | -0.89 (-1.09 to -0.68) |
| Timor-Leste | 0.78(0.54 to 1.17) | 0.79(0.55 to 1.18) | 0.97(0.64 to 1.48) | 1.14(0.75 to 1.82) | 0.9 (0.66 to 1.14) | 1.57 (1.35 to 1.78) |
| Togo | 3.42(1.44 to 7.2) | 3.99(1.77 to 8) | 4.4(2.01 to 8.71) | 4.24(1.91 to 8.23) | 1.4 (1.29 to 1.5) | 0.82 (0.65 to 0.99) |
| Tokelau | 2.87(1.37 to 4.97) | 3.33(1.58 to 5.63) | 3.6(1.63 to 6.05) | 3.59(1.62 to 5.98) | 1.29 (1.18 to 1.41) | 0.83 (0.69 to 0.97) |
| Tonga | 2.58(1.28 to 3.95) | 2.95(1.49 to 4.6) | 3.05(1.47 to 4.7) | 3.15(1.47 to 4.93) | 1.17 (0.9 to 1.43) | 0.82 (0.67 to 0.98) |
| Trinidad and Tobago | 6.15(2.87 to 10.13) | 5.64(2.66 to 9.09) | 4.56(2.25 to 7.1) | 4.61(2.09 to 7.49) | -1.42 (-1.65 to -1.18) | -1.41 (-1.61 to -1.22) |
| Tunisia | 2.06(0.8 to 4.69) | 2.14(0.79 to 5) | 2.06(0.78 to 4.86) | 1.94(0.73 to 4.58) | -0.23 (-0.36 to -0.1) | -0.34 (-0.41 to -0.27) |
| Turkey | 2.55(1.11 to 4.94) | 1.8(0.76 to 3.54) | 1.77(0.76 to 3.32) | 1.63(0.72 to 3.17) | -2.47 (-2.91 to -2.02) | -1.46 (-1.81 to -1.12) |
| Turkmenistan | 11.43(2.25 to 26.46) | 13.08(2.57 to 30.96) | 11.61(2.61 to 25.66) | 12.6(2.94 to 27.29) | 0.01 (-0.61 to 0.62) | -0.41 (-0.74 to -0.08) |
| Tuvalu | 3.4(1.71 to 5.91) | 3.98(1.81 to 6.69) | 4.21(1.93 to 7.06) | 4.25(1.91 to 7.26) | 1.17 (1.03 to 1.3) | 0.76 (0.63 to 0.89) |
| Uganda | 2.23(1.07 to 3.95) | 2.73(1.29 to 4.73) | 2.53(1.27 to 4.43) | 2.53(1.24 to 4.5) | 0.73 (0.38 to 1.07) | 0.17 (-0.05 to 0.39) |
| Ukraine | 5.61(1.24 to 14.76) | 6.92(1.48 to 19.2) | 7.8(1.48 to 21) | 8.38(1.65 to 21.41) | 1.73 (1.43 to 2.04) | 1.16 (0.92 to 1.4) |
| United Arab Emirates | 7.25(3.28 to 14.13) | 7.92(3.59 to 15.27) | 6.2(2.88 to 11.25) | 3.78(1.68 to 7.09) | -0.3 (-0.82 to 0.21) | -2.51 (-3.17 to -1.84) |
| United Kingdom | 14.22(3.98 to 23.6) | 10.13(3.22 to 16.27) | 6.46(2.44 to 10.12) | 5.93(2.35 to 9.22) | -3.95 (-4.1 to -3.8) | -3.48 (-3.69 to -3.26) |
| United Republic of Tanzania | 2.39(1.12 to 4.26) | 2.32(1.11 to 4.14) | 2.44(1.16 to 4.45) | 2.57(1.22 to 4.79) | 0.13 (0.02 to 0.24) | 0.33 (0.26 to 0.41) |
| United States of America | 13.03(4.28 to 21.46) | 12.57(4.86 to 19.43) | 9.45(3.92 to 14.42) | 8.84(3.66 to 13.49) | -1.47 (-1.79 to -1.15) | -1.74 (-1.93 to -1.55) |
| United States Virgin Islands | 3.22(1.39 to 6.38) | 3.14(1.4 to 5.94) | 3.63(1.66 to 6.8) | 3.38(1.53 to 6.31) | 0.39 (0.05 to 0.74) | 0.38 (0.19 to 0.56) |
| Uruguay | 7.68(3.35 to 13.85) | 6.21(3 to 10.45) | 5.61(2.95 to 8.98) | 5.5(3.04 to 8.53) | -1.57 (-1.71 to -1.42) | -1.14 (-1.28 to -1) |
| Uzbekistan | 8.48(1.87 to 19.48) | 14.06(3.44 to 33.13) | 19.69(5.18 to 44.75) | 18.81(5.36 to 42.35) | 4.08 (3.66 to 4.49) | 2.92 (2.49 to 3.35) |
| Vanuatu | 2.54(1.28 to 5.06) | 3.04(1.46 to 5.86) | 3.53(1.64 to 6.68) | 3.72(1.75 to 7.01) | 1.41 (1.24 to 1.58) | 1.3 (1.2 to 1.41) |
| Venezuela (Bolivarian Republic of) | 2.94(1.23 to 5.98) | 2.49(1.06 to 4.91) | 2.67(1.17 to 5.09) | 3.01(1.25 to 5.93) | -0.63 (-1.03 to -0.22) | 0.04 (-0.24 to 0.31) |
| Viet Nam | 0.48(0.4 to 0.58) | 0.45(0.38 to 0.54) | 0.51(0.44 to 0.63) | 0.68(0.54 to 0.88) | 0.26 (0.02 to 0.49) | 1.31 (0.99 to 1.63) |
| Yemen | 2.45(1.09 to 5.38) | 2.2(0.99 to 4.73) | 2.06(0.91 to 4.39) | 2.09(0.98 to 4.45) | -0.87 (-0.95 to -0.79) | -0.58 (-0.67 to -0.49) |
| Zambia | 2.78(1.29 to 4.92) | 3.12(1.46 to 5.46) | 2.91(1.35 to 5.14) | 2.65(1.23 to 4.68) | 0.11 (-0.13 to 0.35) | -0.43 (-0.61 to -0.24) |
| Zimbabwe | 2.07(0.92 to 3.79) | 2.43(1.13 to 4.39) | 3.12(1.39 to 5.64) | 3.02(1.34 to 5.54) | 2.62 (2.27 to 2.98) | 1.85 (1.56 to 2.13) |

ASMR, age-standard morality rate; EAPC, estimated annual percentage change.
