# Supplementary material for: Global, regional, and national burden of diet high in processed meat from 1990 to 2019: a systematic analysis from the global burden of disease study 2019
Source: Front Nutr. 2024 Feb 13;11:1354287. doi: 10.3389/fnut.2024.1354287 (PMC10896824; doi:10.3389/fnut.2024.1354287)
Supplement: Supplementary file 9 [file Table_5.docx]

Table 5S. Age-standardized DALYs of diet high in processed meat for both sexes combined in 1990,2000,2010, and 2019, and EAPC of ASDR from 1990 to 2010 and 1990 to 2019 in 204 countries and territories

| Location | DALYs 1990 | DALYs 2000 | DALYs 2010 | DALYs 2019 | EAPC 1990-2010 | EAPC 1990-2019 |
| --- | --- | --- | --- | --- | --- | --- |
| Afghanistan | 92.12(36.79 to 204.19) | 88.22(39.12 to 185.01) | 83.08(38.26 to 165.68) | 82.4(35 to 162.13) | -0.37 (-0.44 to -0.31) | -0.36 (-0.4 to -0.33) |
| Albania | 252.86(78.48 to 409.98) | 246.83(81.41 to 389.16) | 254.5(82.24 to 406.95) | 252.47(90.79 to 417.11) | 0.58 (0.19 to 0.97) | 0.26 (0.05 to 0.46) |
| Algeria | 67.2(27.76 to 145.82) | 58.8(23.97 to 125.95) | 56.96(23.63 to 117.21) | 56.92(24.06 to 112.21) | -0.87 (-1 to -0.74) | -0.53 (-0.65 to -0.42) |
| American Samoa | 98.23(44.7 to 157.46) | 115.88(52.72 to 179.22) | 125.7(56.64 to 194.1) | 127.57(58.92 to 202.88) | 1.43 (1.29 to 1.58) | 0.93 (0.77 to 1.08) |
| Andorra | 159.39(78.73 to 250.55) | 140.79(75.97 to 211.15) | 130.86(78 to 193.55) | 132.79(80.82 to 194.49) | -1.1 (-1.18 to -1.02) | -0.74 (-0.86 to -0.63) |
| Angola | 75.33(33.63 to 133.11) | 77.86(35.87 to 135.63) | 83.65(41.25 to 143.66) | 84.16(42.26 to 144.68) | 0.46 (0.36 to 0.56) | 0.48 (0.41 to 0.54) |
| Antigua and Barbuda | 69.28(32.39 to 112.83) | 68.2(31.68 to 104.13) | 68.29(32.81 to 102.36) | 69.03(32.63 to 101.77) | -0.34 (-0.58 to -0.11) | -0.23 (-0.34 to -0.11) |
| Argentina | 122.97(56.86 to 233.42) | 114.03(60.23 to 191.72) | 112.54(63.45 to 176.2) | 130.9(78.56 to 197.36) | -0.63 (-0.78 to -0.48) | 0.1 (-0.12 to 0.33) |
| Armenia | 135.64(50.81 to 300.68) | 149.73(66.46 to 298.24) | 159.87(75.37 to 301.18) | 152.24(71.3 to 284.32) | 0.35 (0.09 to 0.61) | 0.07 (-0.08 to 0.22) |
| Australia | 207.77(84.27 to 354.58) | 157.51(83.41 to 245.61) | 134.99(78.98 to 192.55) | 126.34(76.55 to 175.89) | -2.12 (-2.27 to -1.98) | -1.71 (-1.86 to -1.56) |
| Austria | 192.1(83.23 to 325.04) | 198.44(89.41 to 312.48) | 171.75(92.6 to 251.78) | 142.99(82.72 to 209.63) | -0.48 (-0.73 to -0.23) | -1.21 (-1.44 to -0.98) |
| Azerbaijan | 231.42(72.76 to 498.09) | 267.11(92.11 to 568.72) | 312.43(117.96 to 610.33) | 310.17(121.61 to 606.29) | 0.85 (0.54 to 1.17) | 0.89 (0.72 to 1.06) |
| Bahamas | 78.39(35.74 to 133.37) | 74.15(34.31 to 121.14) | 68(31.88 to 108.99) | 70.04(32.11 to 109.81) | -0.86 (-1.01 to -0.71) | -0.57 (-0.69 to -0.45) |
| Bahrain | 126.36(52.63 to 249.51) | 122.23(53.6 to 213.59) | 122.07(57.31 to 191.14) | 111.24(50.27 to 169.37) | -0.13 (-0.43 to 0.17) | -0.53 (-0.72 to -0.35) |
| Bangladesh | 105.5(49.98 to 202.03) | 110.25(54.93 to 199.78) | 129.16(62.37 to 233.24) | 122.56(60.03 to 229.91) | 1.73 (1.43 to 2.03) | 0.84 (0.56 to 1.12) |
| Barbados | 93.1(47.38 to 145.91) | 86.42(45.72 to 129.3) | 78.6(41.53 to 113.75) | 81.95(40.52 to 120.04) | -0.84 (-0.99 to -0.7) | -0.71 (-0.83 to -0.6) |
| Belarus | 404.8(100.96 to 712.12) | 473.88(114.2 to 862.33) | 451.54(100.26 to 834.49) | 355.78(88.36 to 652.68) | 0.16 (-0.34 to 0.66) | -0.95 (-1.35 to -0.55) |
| Belgium | 234.47(99.75 to 366.62) | 199.24(98.28 to 292.93) | 163.72(93.17 to 230.05) | 150.55(89.14 to 208.56) | -1.72 (-1.79 to -1.65) | -1.72 (-1.8 to -1.65) |
| Belize | 62.03(27.74 to 105.26) | 93.17(44.11 to 152.35) | 83.36(41.51 to 127.64) | 80.65(39.11 to 123.31) | 1.81 (1.17 to 2.46) | 0.68 (0.25 to 1.11) |
| Benin | 87.81(42.15 to 161.24) | 108.85(56.01 to 189) | 119.58(63.31 to 201.6) | 118.01(62.73 to 197.1) | 1.61 (1.44 to 1.78) | 1.1 (0.92 to 1.27) |
| Bermuda | 77.45(35.29 to 149.36) | 51.69(24.8 to 91.05) | 41.45(20.74 to 67) | 41.17(19.9 to 65.87) | -3.2 (-3.46 to -2.95) | -2.24 (-2.55 to -1.94) |
| Bhutan | 110.04(46.91 to 223.8) | 123.98(55.31 to 238.53) | 140.51(66.96 to 257.65) | 154.97(78.78 to 270.26) | 1.21 (1.17 to 1.25) | 1.2 (1.18 to 1.23) |
| Bolivia (Plurinational State of) | 32.02(19.37 to 50.59) | 30.57(18.15 to 46) | 31.23(17.86 to 46.98) | 33.68(18.16 to 50.52) | -0.34 (-0.47 to -0.21) | 0.14 (-0.01 to 0.29) |
| Bosnia and Herzegovina | 184.98(76.33 to 346.19) | 193.19(90.86 to 337.01) | 236(137.66 to 359.53) | 243.25(141.28 to 380.78) | 1.32 (0.99 to 1.65) | 1.1 (0.92 to 1.27) |
| Botswana | 69.61(31.1 to 120.42) | 104.81(47.5 to 183.16) | 103.45(49.05 to 170.35) | 102.98(46.86 to 169.79) | 2 (1.41 to 2.59) | 1.11 (0.74 to 1.48) |
| Brazil | 75.31(34.92 to 139.65) | 66.61(31.42 to 117.57) | 76.29(39.37 to 127.31) | 79.35(42.84 to 126.25) | 0.02 (-0.29 to 0.33) | 0.54 (0.33 to 0.75) |
| Brunei Darussalam | 306.51(185.46 to 459.31) | 271.67(169.37 to 389.78) | 272.25(165.53 to 386.21) | 240.82(146.66 to 348.41) | -0.16 (-0.47 to 0.16) | -0.51 (-0.69 to -0.32) |
| Bulgaria | 292.33(108.56 to 568.16) | 282.33(102.55 to 568.35) | 305.33(129.81 to 527.53) | 324.58(140.14 to 550.02) | -0.03 (-0.28 to 0.22) | 0.3 (0.14 to 0.45) |
| Burkina Faso | 87.6(42.19 to 156.26) | 102.94(52.14 to 182.5) | 111.15(58.13 to 195.53) | 120.6(64.81 to 211.99) | 1.34 (1.21 to 1.47) | 1.15 (1.07 to 1.24) |
| Burundi | 83.53(36.74 to 151.93) | 80.77(35.83 to 145.39) | 71.36(31.57 to 130.49) | 68.02(29.58 to 120.96) | -1.03 (-1.21 to -0.85) | -0.96 (-1.05 to -0.87) |
| Cabo Verde | 63.17(27.59 to 130.76) | 84.34(42.16 to 153.64) | 93.55(50.57 to 154.97) | 113.59(59.53 to 189.96) | 2.08 (1.78 to 2.37) | 1.75 (1.57 to 1.93) |
| Cambodia | 26.46(17.56 to 40.27) | 26.83(17.34 to 41.83) | 28.49(17.96 to 44.32) | 31.78(19.25 to 48.79) | 0.31 (0.26 to 0.37) | 0.61 (0.52 to 0.7) |
| Cameroon | 97.86(51.47 to 165.55) | 127.23(68.72 to 208.65) | 144.61(81.73 to 230.8) | 140.21(77.01 to 225.9) | 2.29 (2.07 to 2.51) | 1.42 (1.15 to 1.7) |
| Canada | 188.26(72.51 to 318.17) | 163.14(78.93 to 254.5) | 126.12(69.34 to 186.38) | 122.92(71.01 to 179.37) | -1.95 (-2.11 to -1.79) | -1.88 (-2.04 to -1.73) |
| Central African Republic | 97.58(43.94 to 178.16) | 102.76(44.76 to 187.5) | 108.75(50.12 to 196.93) | 102.45(46.4 to 188.57) | 0.63 (0.57 to 0.7) | 0.33 (0.23 to 0.44) |
| Chad | 72.08(34.93 to 135.52) | 94.97(47.51 to 167.87) | 106.66(53.49 to 186.84) | 104.85(53.76 to 185.31) | 2.13 (1.89 to 2.38) | 1.35 (1.1 to 1.6) |
| Chile | 187(96.94 to 292.04) | 176.66(102.15 to 248.69) | 193.2(118.51 to 264.35) | 189.26(118.83 to 254.9) | 0.43 (0.26 to 0.6) | 0.29 (0.19 to 0.38) |
| China | 20.99(11.92 to 34.77) | 21.8(11.75 to 36.41) | 34.28(16.05 to 62.53) | 38.18(17.78 to 70.27) | 2.74 (2.2 to 3.28) | 2.8 (2.51 to 3.09) |
| Colombia | 53.99(22.69 to 100.19) | 52.01(23 to 87.69) | 50.33(23.14 to 85.04) | 54.43(26.21 to 90.73) | -0.62 (-0.76 to -0.48) | -0.23 (-0.37 to -0.08) |
| Comoros | 73.71(34.22 to 138.88) | 74.3(36.28 to 133.69) | 68.27(33.64 to 119.32) | 71.38(34.1 to 127.43) | -0.51 (-0.69 to -0.33) | -0.25 (-0.37 to -0.13) |
| Congo | 114.33(54.22 to 204.3) | 110.74(53.42 to 195.36) | 102.44(49.24 to 185.95) | 100.59(48.6 to 176.19) | -0.6 (-0.78 to -0.43) | -0.53 (-0.62 to -0.44) |
| Cook Islands | 102.61(44.13 to 157.17) | 107.02(48.8 to 157.52) | 109.01(48.47 to 159.99) | 114.11(50.24 to 172.41) | 0.37 (0.29 to 0.44) | 0.32 (0.28 to 0.36) |
| Costa Rica | 56.9(26.18 to 104.46) | 52.11(25.69 to 91.13) | 50.72(25.24 to 85.17) | 55.63(28.42 to 91.95) | -1.07 (-1.27 to -0.86) | -0.38 (-0.62 to -0.15) |
| Croatia | 62.17(24.13 to 133.37) | 48.89(20.48 to 96.11) | 46.67(20.33 to 87.29) | 48.29(22.22 to 87.99) | -1.46 (-1.7 to -1.23) | -0.86 (-1.07 to -0.65) |
| Cuba | 74.99(32.31 to 141.25) | 58.96(26.15 to 110.91) | 57.16(26.99 to 102.62) | 60.86(30.07 to 108.78) | -1.68 (-1.99 to -1.37) | -0.8 (-1.1 to -0.5) |
| Cyprus | 296.9(155.99 to 480.42) | 241.97(136.05 to 388.63) | 173.07(100.68 to 268.58) | 156.73(92.36 to 236.1) | -2.96 (-3.15 to -2.77) | -2.74 (-2.9 to -2.58) |
| Czechia | 295.09(114.35 to 542.33) | 218.76(98.18 to 390.09) | 200.2(106.66 to 319.05) | 205.57(119.73 to 324.48) | -1.95 (-2.19 to -1.72) | -1.19 (-1.43 to -0.95) |
| C么te d'Ivoire | 121.58(60.74 to 223.47) | 150.44(77.96 to 258.69) | 144.81(76.98 to 243.12) | 136.94(74.92 to 225.93) | 0.97 (0.64 to 1.31) | 0.28 (0.03 to 0.53) |
| Democratic People's Republic of Korea | 33.37(14.45 to 66.36) | 36.59(15.97 to 73.5) | 41.31(17.59 to 83.95) | 40.07(17.02 to 80.37) | 1.15 (1.1 to 1.2) | 0.76 (0.64 to 0.89) |
| Democratic Republic of the Congo | 87.39(42.19 to 160.94) | 76.98(36.65 to 140.56) | 71.12(32.56 to 131.6) | 72.91(33.71 to 129.9) | -1.09 (-1.16 to -1.01) | -0.7 (-0.82 to -0.58) |
| Denmark | 285.56(100.22 to 467.2) | 216.74(103.34 to 323.47) | 161.18(96.11 to 222.88) | 143.44(89.45 to 195.97) | -2.92 (-3.02 to -2.81) | -2.74 (-2.88 to -2.6) |
| Djibouti | 54.48(24.47 to 98.93) | 62.74(28.15 to 112.86) | 75.21(36.43 to 134.85) | 76.82(38.3 to 133.82) | 1.7 (1.62 to 1.77) | 1.33 (1.2 to 1.45) |
| Dominica | 89.43(41.95 to 143) | 94.07(43.71 to 144.4) | 91.61(44.85 to 135.97) | 94.25(45.74 to 140.35) | 0.02 (-0.1 to 0.14) | 0.04 (-0.02 to 0.09) |
| Dominican Republic | 47.41(19.1 to 94.55) | 47.63(20.06 to 91.14) | 62.05(25.56 to 123.86) | 77.55(31.53 to 155.01) | 1.8 (1.45 to 2.16) | 2.26 (2.03 to 2.49) |
| Ecuador | 41.22(20.54 to 71.42) | 46.42(23.69 to 74.42) | 55.61(30.17 to 86.61) | 56.3(30.19 to 88.78) | 1.4 (1.18 to 1.63) | 1.09 (0.95 to 1.24) |
| Egypt | 86.09(29.92 to 198.56) | 84.38(29.3 to 191.38) | 113.9(40.43 to 257.04) | 124.62(43.86 to 284.16) | 1.49 (1.19 to 1.78) | 1.71 (1.54 to 1.88) |
| El Salvador | 51.75(20.7 to 101.58) | 61.78(28.16 to 105.72) | 70.56(35.09 to 111.63) | 78.6(37.01 to 127.87) | 1.64 (1.46 to 1.82) | 1.45 (1.33 to 1.56) |
| Equatorial Guinea | 88.55(40.13 to 169.37) | 86.05(42.36 to 151.89) | 104.47(56.56 to 169.22) | 108.6(58.4 to 172.27) | 0.98 (0.67 to 1.28) | 1.11 (0.95 to 1.27) |
| Eritrea | 70.29(31.12 to 126.09) | 81.93(38.69 to 144.6) | 84.48(40.25 to 151.75) | 84.58(39.85 to 148.65) | 1.07 (0.88 to 1.26) | 0.57 (0.41 to 0.73) |
| Estonia | 585.35(146.01 to 977.92) | 539.27(157.94 to 883.51) | 341.31(136.72 to 544.08) | 276.16(124.64 to 439.07) | -2.64 (-3.22 to -2.06) | -3.4 (-3.76 to -3.04) |
| Eswatini | 90.86(43.09 to 141.6) | 124.41(59.75 to 192.74) | 155.22(70.02 to 243.4) | 134.89(61.9 to 208.06) | 3.69 (3.19 to 4.19) | 1.83 (1.27 to 2.4) |
| Ethiopia | 82.58(39.79 to 139.58) | 67.08(32.92 to 113.04) | 53.78(27.04 to 89.85) | 53.74(27.58 to 90.39) | -2.17 (-2.22 to -2.12) | -1.75 (-1.9 to -1.61) |
| Fiji | 147.43(66.7 to 247.77) | 211.74(99.52 to 317.94) | 214.18(101.09 to 318.36) | 211.17(93.65 to 323.37) | 1.77 (1.4 to 2.15) | 0.98 (0.69 to 1.26) |
| Finland | 263.85(82.19 to 467.12) | 231.05(87.46 to 367.99) | 194(92.97 to 291.91) | 159.65(84.96 to 236.2) | -1.18 (-1.35 to -1.02) | -1.72 (-1.89 to -1.55) |
| France | 153.72(70.38 to 232.2) | 141.64(74.72 to 204.23) | 118.97(69.46 to 165.37) | 106.31(64.88 to 146.79) | -1.06 (-1.24 to -0.88) | -1.36 (-1.49 to -1.24) |
| Gabon | 113.6(58.34 to 185.22) | 128.51(68.62 to 204.22) | 129.92(70.14 to 208.47) | 123.51(64.69 to 196.33) | 0.92 (0.75 to 1.09) | 0.28 (0.09 to 0.48) |
| Gambia | 91.13(42.21 to 175.47) | 104.95(52.61 to 192.38) | 120.63(61.3 to 217.39) | 130.17(66.05 to 233.29) | 1.66 (1.46 to 1.87) | 1.22 (1.06 to 1.39) |
| Georgia | 272.09(77.47 to 589.95) | 251.39(77.92 to 537.73) | 238.09(100.37 to 441.09) | 227.01(110.01 to 407.07) | -0.86 (-1.08 to -0.65) | -0.83 (-0.95 to -0.7) |
| Germany | 354.85(147.96 to 542.19) | 272.9(136.18 to 398.65) | 212.42(119.49 to 302.09) | 207.22(125.41 to 291.44) | -2.64 (-2.71 to -2.57) | -2.16 (-2.33 to -1.98) |
| Ghana | 96.03(47.18 to 179.37) | 123.87(66.72 to 213.76) | 166.25(95.72 to 263.38) | 158.96(89.66 to 258.63) | 3.14 (2.98 to 3.31) | 2.12 (1.81 to 2.44) |
| Greece | 130.72(52.98 to 241.51) | 127.81(56.01 to 232.04) | 143.81(64.42 to 240.63) | 139(68.42 to 225.98) | 0.69 (0.48 to 0.9) | 0.4 (0.26 to 0.54) |
| Greenland | 385.35(139.9 to 604.89) | 350.54(149.51 to 530.25) | 273.54(132.9 to 405.04) | 250.36(129.51 to 369.15) | -1.97 (-2.23 to -1.71) | -1.86 (-2.01 to -1.72) |
| Grenada | 94.01(42.36 to 157.07) | 83.74(40.12 to 128.66) | 89.93(43.96 to 134.88) | 90.77(43.35 to 135.3) | -0.3 (-0.52 to -0.07) | -0.05 (-0.18 to 0.08) |
| Guam | 69.61(30.39 to 129.36) | 65.42(27.95 to 123.69) | 69.96(29.61 to 134.8) | 77.53(32.7 to 152.53) | -0.02 (-0.29 to 0.24) | 0.44 (0.26 to 0.63) |
| Guatemala | 55.43(21.99 to 113.4) | 74.02(34.29 to 118.98) | 94.9(47.25 to 142.86) | 98.84(48.97 to 148.43) | 2.3 (2.06 to 2.53) | 2.01 (1.83 to 2.18) |
| Guinea | 87.77(44.69 to 157.47) | 100.9(53.19 to 172.45) | 120.5(63.74 to 210.76) | 125.08(65.55 to 215.06) | 1.77 (1.69 to 1.84) | 1.51 (1.41 to 1.61) |
| Guinea-Bissau | 131.84(61.28 to 252.53) | 147.02(70.22 to 268.4) | 158.11(79.93 to 280.2) | 151.49(76.4 to 270.08) | 1.02 (0.94 to 1.1) | 0.56 (0.42 to 0.7) |
| Guyana | 139.27(63.19 to 249.84) | 173.91(80.42 to 279.96) | 157.47(74.48 to 258.06) | 154.27(73.03 to 252.45) | 0.93 (0.52 to 1.34) | 0.18 (-0.1 to 0.46) |
| Haiti | 104.55(45.47 to 186.45) | 89.57(37.4 to 154.51) | 92.91(38.84 to 161.15) | 93.15(39.2 to 158.97) | -0.49 (-0.73 to -0.25) | -0.16 (-0.31 to -0.02) |
| Honduras | 51.42(20.86 to 97.83) | 58.09(23.86 to 106.63) | 64.79(28.15 to 118.57) | 71.39(32.31 to 126.65) | 1.1 (0.99 to 1.22) | 1.21 (1.13 to 1.3) |
| Hungary | 264.38(97.41 to 512.39) | 227.91(99.88 to 418.82) | 263.22(129.97 to 425.6) | 245.03(126.07 to 388.84) | 0.2 (-0.22 to 0.62) | 0.04 (-0.17 to 0.25) |
| Iceland | 222.86(70.59 to 369.43) | 163.01(63.77 to 261.01) | 129.94(64.85 to 197.7) | 127.04(69.48 to 187.38) | -2.77 (-2.87 to -2.67) | -2.15 (-2.35 to -1.94) |
| India | 48.11(33.18 to 73.84) | 52.67(36.63 to 78.53) | 60.25(41.9 to 87.41) | 64(44.52 to 92.6) | 0.99 (0.78 to 1.2) | 1.18 (1.05 to 1.31) |
| Indonesia | 18.86(15.99 to 22.78) | 19.83(16.97 to 23.65) | 25.84(20.45 to 32.9) | 31.27(22.29 to 41.35) | 1.56 (1.28 to 1.83) | 2.14 (1.93 to 2.35) |
| Iran (Islamic Republic of) | 58.52(25.16 to 127) | 57.3(24.73 to 120.43) | 49.07(22.86 to 91.96) | 50.9(24.32 to 89.52) | -0.83 (-1 to -0.66) | -0.75 (-0.88 to -0.62) |
| Iraq | 87.08(36.57 to 172.89) | 78.22(33.38 to 151.75) | 73.82(33.05 to 136.88) | 73(33.88 to 132.81) | -0.77 (-0.85 to -0.69) | -0.7 (-0.77 to -0.63) |
| Ireland | 240.28(75.79 to 439.89) | 207.72(74.44 to 345.75) | 148.64(71.05 to 224.05) | 133.9(73.8 to 197.97) | -2.14 (-2.36 to -1.92) | -2.33 (-2.47 to -2.18) |
| Israel | 133.52(60.3 to 255.55) | 136.43(80.91 to 204.58) | 104.27(66.95 to 147.18) | 102.96(67.02 to 142.39) | -1.29 (-1.62 to -0.97) | -1.44 (-1.62 to -1.25) |
| Italy | 200.46(107.76 to 301.14) | 174.73(100.57 to 250.33) | 162.06(103.35 to 221.54) | 147.6(95.04 to 201.65) | -0.84 (-0.96 to -0.71) | -1.07 (-1.17 to -0.97) |
| Jamaica | 73.89(36.7 to 107.89) | 94.78(48.6 to 132.63) | 86.22(44.11 to 121.44) | 93.6(46.18 to 140.89) | 0.88 (0.4 to 1.36) | 0.56 (0.3 to 0.81) |
| Japan | 110.92(62.86 to 165.39) | 111.39(67.04 to 155.55) | 100.16(61.76 to 139.25) | 89.62(57.84 to 124.31) | -0.52 (-0.66 to -0.38) | -0.83 (-0.95 to -0.72) |
| Jordan | 72.79(32.31 to 128.98) | 74.18(33.88 to 131.03) | 60.05(27.6 to 100.15) | 53.76(24.15 to 90.2) | -0.71 (-1.09 to -0.33) | -1.41 (-1.68 to -1.14) |
| Kazakhstan | 264.91(82.28 to 521.13) | 346.12(99.22 to 714.77) | 291.04(106.06 to 574.92) | 238.58(112.08 to 419.03) | -0.01 (-0.77 to 0.74) | -1.16 (-1.63 to -0.68) |
| Kenya | 45.51(24.75 to 73.07) | 53.4(28.88 to 85.83) | 64.25(34.47 to 105.63) | 63.5(34.77 to 104.36) | 2.08 (1.88 to 2.27) | 1.37 (1.15 to 1.6) |
| Kiribati | 148.75(67.03 to 261.76) | 187.85(85.43 to 309.47) | 194.17(86.82 to 317.15) | 191.67(88.25 to 319.91) | 1.48 (1.23 to 1.73) | 0.71 (0.48 to 0.95) |
| Kuwait | 62.39(25.89 to 121.48) | 73.83(31.42 to 139.47) | 62.14(27 to 114.25) | 56.73(24.5 to 101.81) | 1.01 (0.45 to 1.57) | -0.38 (-0.84 to 0.08) |
| Kyrgyzstan | 187.55(59.02 to 383.86) | 203.26(62.53 to 431.37) | 223.8(65.26 to 482.83) | 190.15(65.27 to 385.3) | 0.63 (0.21 to 1.04) | -0.01 (-0.29 to 0.28) |
| Lao People's Democratic Republic | 35.7(22.62 to 56.97) | 37.82(24.46 to 57.59) | 39.38(24.05 to 61.99) | 40.34(24.23 to 63.18) | 0.58 (0.54 to 0.62) | 0.38 (0.33 to 0.44) |
| Latvia | 630.04(149.45 to 1076.03) | 582.2(156.34 to 981.06) | 516.47(160.75 to 843.3) | 403.6(154.58 to 665.61) | -1.39 (-2.08 to -0.7) | -2.11 (-2.49 to -1.73) |
| Lebanon | 79.51(29.97 to 175.98) | 64.06(26.13 to 137.41) | 65.88(27.72 to 132.44) | 65.73(27.21 to 132.99) | -1.03 (-1.35 to -0.72) | -0.45 (-0.67 to -0.23) |
| Lesotho | 61.11(26.62 to 97.91) | 83.7(38.02 to 132.41) | 130.82(60.83 to 214.49) | 137.08(63.67 to 226.91) | 4.65 (4.21 to 5.09) | 3.64 (3.25 to 4.03) |
| Liberia | 107.76(53.79 to 192.4) | 101.11(52.72 to 177.39) | 119.2(62.68 to 204.86) | 114.48(61.58 to 194.5) | 0.38 (0.1 to 0.67) | 0.56 (0.38 to 0.74) |
| Libya | 54.96(22.28 to 115.68) | 55.56(23.63 to 110.82) | 59.85(25.27 to 111.29) | 59.92(25.06 to 112.83) | 0.52 (0.29 to 0.76) | 0.5 (0.38 to 0.61) |
| Lithuania | 607.02(139.46 to 1051.15) | 525.01(136.09 to 905.66) | 519.11(141.6 to 883.52) | 402.06(120.27 to 689.45) | -1 (-1.58 to -0.42) | -1.54 (-1.87 to -1.22) |
| Luxembourg | 291.35(109.51 to 449.54) | 231.51(108.54 to 347.73) | 186.41(108.72 to 268.35) | 173.21(108.74 to 243.86) | -2.2 (-2.28 to -2.11) | -2.01 (-2.11 to -1.92) |
| Madagascar | 70.52(31.45 to 135.7) | 71.06(30.43 to 137.77) | 73.28(31.1 to 150.27) | 71.76(31.37 to 139.11) | 0.24 (0.15 to 0.33) | 0.07 (0.01 to 0.14) |
| Malawi | 70.15(32.26 to 121.26) | 84.62(40.06 to 147.02) | 82.95(39.83 to 144.67) | 75.93(38.48 to 131.52) | 0.96 (0.67 to 1.24) | 0.21 (-0.03 to 0.46) |
| Malaysia | 61.24(26.7 to 116.13) | 67.34(28.43 to 129.74) | 67.96(28.02 to 136.25) | 75.53(32.51 to 147.74) | 0.57 (0.39 to 0.74) | 0.57 (0.47 to 0.67) |
| Maldives | 43.53(28.35 to 70.15) | 33.63(22.56 to 51.64) | 24.71(15.48 to 38.38) | 24.53(14.77 to 39.17) | -3.38 (-3.62 to -3.13) | -2.52 (-2.82 to -2.22) |
| Mali | 94.46(48.49 to 168.17) | 105.82(56.5 to 176.61) | 118.52(64.84 to 193.38) | 121.22(69.24 to 198.96) | 1.14 (1.1 to 1.19) | 0.92 (0.85 to 0.99) |
| Malta | 385.25(162.49 to 581.01) | 309.53(147.06 to 460.72) | 252.11(136.36 to 363.66) | 222.69(128.46 to 316.87) | -1.9 (-2 to -1.8) | -1.89 (-1.95 to -1.83) |
| Marshall Islands | 104.41(48.26 to 183.04) | 126.9(56.13 to 215.64) | 143.47(63.16 to 244.1) | 147.86(60.69 to 255.05) | 1.72 (1.6 to 1.84) | 1.22 (1.07 to 1.37) |
| Mauritania | 123.5(60.6 to 227.47) | 116.75(62.19 to 198.71) | 114.76(62.96 to 192.62) | 110.46(58.5 to 182.87) | -0.32 (-0.4 to -0.25) | -0.33 (-0.37 to -0.28) |
| Mauritius | 63.13(35.23 to 106.22) | 61.64(32.71 to 100.81) | 82.02(42.1 to 117.35) | 74.43(37.53 to 109.61) | 1.78 (1.36 to 2.19) | 1.11 (0.82 to 1.4) |
| Mexico | 116.74(67.9 to 165.75) | 111.39(64.1 to 156.67) | 115.47(66.19 to 163.53) | 127.12(75.09 to 181.78) | -0.13 (-0.25 to -0.01) | 0.24 (0.1 to 0.38) |
| Micronesia (Federated States of) | 104.72(45.81 to 185.26) | 131.62(59.11 to 218.72) | 148.98(69.64 to 247.34) | 158.31(69.3 to 272.2) | 2.03 (1.85 to 2.21) | 1.46 (1.28 to 1.64) |
| Monaco | 204.13(77.8 to 325.69) | 170.06(78.17 to 265.46) | 162.13(84.35 to 237.94) | 159.81(88.86 to 228.93) | -1.42 (-1.63 to -1.21) | -0.88 (-1.06 to -0.7) |
| Mongolia | 249.1(48.86 to 566.45) | 277.75(53.95 to 643.39) | 206.48(49 to 460.02) | 196.19(50.19 to 435.64) | -1.31 (-1.86 to -0.76) | -1.52 (-1.8 to -1.23) |
| Montenegro | 258.42(108.6 to 422.63) | 290.61(128.8 to 458.65) | 305.75(143.04 to 473.32) | 281.88(140.61 to 441.38) | 1.19 (1 to 1.38) | 0.41 (0.17 to 0.64) |
| Morocco | 43.88(24.61 to 80.46) | 42.74(23.59 to 79.82) | 54.85(22.22 to 112.12) | 64.44(26.34 to 131.78) | 0.91 (0.54 to 1.29) | 1.61 (1.35 to 1.88) |
| Mozambique | 54.58(25.43 to 92.39) | 58.74(28.31 to 97.33) | 79.65(38.25 to 133.55) | 84.66(40.29 to 145.19) | 1.9 (1.58 to 2.22) | 2.06 (1.86 to 2.26) |
| Myanmar | 36.45(23.38 to 54.97) | 36.66(23.84 to 54.5) | 37.07(22.63 to 55.26) | 36.9(22.58 to 53.12) | 0.05 (0.01 to 0.09) | 0.01 (-0.02 to 0.03) |
| Namibia | 65.89(28.71 to 110.79) | 81.76(37.38 to 136.66) | 69.26(30.57 to 112.16) | 68.5(30.05 to 113.29) | 0.66 (0.13 to 1.2) | -0.14 (-0.47 to 0.2) |
| Nauru | 148.84(62.53 to 280.8) | 177.88(79.06 to 321.92) | 182.62(77.41 to 332.15) | 189.97(81.97 to 341.44) | 1.14 (0.93 to 1.36) | 0.73 (0.57 to 0.88) |
| Nepal | 91.23(35.75 to 192.79) | 97.86(41.95 to 197.46) | 112.27(50.97 to 216.26) | 130.9(63.22 to 241.89) | 0.91 (0.77 to 1.04) | 1.31 (1.18 to 1.43) |
| Netherlands | 211.23(95.61 to 343.09) | 175.35(89.56 to 270.08) | 128.75(79.18 to 182.02) | 121.4(76.03 to 169.97) | -2.43 (-2.63 to -2.24) | -2.27 (-2.43 to -2.12) |
| New Zealand | 227.94(87.63 to 392.27) | 169.75(80.86 to 276.02) | 134.27(69.3 to 201.37) | 137.17(78.4 to 197.47) | -2.75 (-2.85 to -2.65) | -2.08 (-2.32 to -1.85) |
| Nicaragua | 52.4(23.58 to 86.14) | 63.17(29.12 to 100.94) | 76.4(36.16 to 121.17) | 82.15(38.6 to 132.06) | 1.69 (1.43 to 1.96) | 1.5 (1.34 to 1.65) |
| Niger | 84.61(37.7 to 163.82) | 91.08(44.86 to 167.66) | 94.36(48.34 to 164.18) | 103.9(53.05 to 183.06) | 0.81 (0.66 to 0.96) | 0.76 (0.68 to 0.85) |
| Nigeria | 90.22(44.39 to 173.25) | 103.47(53.29 to 186.06) | 103.47(54.1 to 176.33) | 102.47(55.02 to 168.74) | 0.68 (0.49 to 0.88) | 0.39 (0.27 to 0.52) |
| Niue | 101.07(45.43 to 174.11) | 122.63(54.89 to 200.57) | 134.5(61.33 to 217.5) | 138.19(61.11 to 220.42) | 1.47 (1.33 to 1.61) | 1.01 (0.87 to 1.15) |
| North Macedonia | 226.59(99.71 to 418.39) | 259.92(124.49 to 453.56) | 279.98(152.73 to 450.26) | 269.59(147.52 to 435.37) | 1.15 (1.01 to 1.3) | 0.55 (0.37 to 0.73) |
| Northern Mariana Islands | 72.11(32.18 to 126.17) | 77.25(36.42 to 130.93) | 91.45(43.53 to 151.3) | 88.98(41.1 to 150) | 1.65 (1.39 to 1.9) | 0.95 (0.72 to 1.18) |
| Norway | 368.54(125.37 to 583.31) | 280.96(128.48 to 426.24) | 202(113.45 to 294.5) | 176.92(110.02 to 250.69) | -3.06 (-3.15 to -2.97) | -2.87 (-2.98 to -2.76) |
| Oman | 92.25(40.56 to 190.14) | 101.76(44.8 to 207.45) | 100.33(44.17 to 192.23) | 80.17(35.64 to 145.65) | 0.51 (0.37 to 0.66) | -0.38 (-0.64 to -0.12) |
| Pakistan | 145.73(64.62 to 276.31) | 205.82(91.32 to 385.36) | 236.44(115.11 to 421.35) | 243.28(120.5 to 440.54) | 2.39 (2.09 to 2.69) | 1.64 (1.39 to 1.89) |
| Palau | 102.26(42.91 to 185.03) | 121.63(52.8 to 210.92) | 135.01(59.85 to 237.45) | 141.67(61.74 to 240.75) | 1.52 (1.42 to 1.63) | 1.17 (1.06 to 1.28) |
| Palestine | 66.53(33.66 to 123.9) | 62.77(33.05 to 111.94) | 56.06(28.91 to 94) | 62.9(32.88 to 100.91) | -0.98 (-1.08 to -0.87) | -0.46 (-0.65 to -0.27) |
| Panama | 44.75(20.3 to 78.2) | 44.82(21.79 to 70.96) | 51.15(24.91 to 79.68) | 56.85(27.83 to 88.07) | 0.57 (0.42 to 0.72) | 0.94 (0.81 to 1.07) |
| Papua New Guinea | 66.36(31.34 to 110.21) | 77.85(36.34 to 127.21) | 87.41(42 to 142.46) | 92.1(42.56 to 149.45) | 1.44 (1.35 to 1.52) | 1.13 (1.03 to 1.22) |
| Paraguay | 48.05(20.23 to 89.67) | 55.6(25.8 to 93.71) | 66.29(31.89 to 107.8) | 71.72(33.45 to 117.55) | 1.78 (1.58 to 1.98) | 1.41 (1.27 to 1.55) |
| Peru | 15.6(11.36 to 23.24) | 12.27(8.98 to 16.95) | 15.43(8.46 to 23.48) | 17.01(8.35 to 26.69) | -0.71 (-1.33 to -0.08) | 0.68 (0.2 to 1.17) |
| Philippines | 47.21(24.36 to 76.89) | 60.33(27.58 to 107.89) | 80.86(36.49 to 152.47) | 93.03(44.27 to 172.71) | 3.16 (2.96 to 3.35) | 2.82 (2.67 to 2.98) |
| Poland | 158.81(58.72 to 341.25) | 124.35(51.78 to 247.64) | 138.8(72.36 to 234.55) | 130.79(71.49 to 207.69) | -0.59 (-1.07 to -0.11) | -0.39 (-0.63 to -0.15) |
| Portugal | 107.57(56.23 to 186.03) | 111.41(64.04 to 173.62) | 109(68.81 to 155.01) | 97.46(61.73 to 138.75) | 0.26 (0.06 to 0.46) | -0.5 (-0.73 to -0.27) |
| Puerto Rico | 80.16(38.77 to 135.16) | 86.4(43.29 to 131.84) | 82.56(42.74 to 121.22) | 78.52(39.21 to 119.32) | 0.24 (0.05 to 0.42) | -0.29 (-0.46 to -0.11) |
| Qatar | 137.48(60.33 to 256.95) | 151.04(66.83 to 259.43) | 157.42(76.34 to 244.73) | 124.27(61.09 to 198.68) | 1.05 (0.69 to 1.41) | -0.14 (-0.52 to 0.23) |
| Republic of Korea | 89.68(43.67 to 164.86) | 104.35(67.37 to 145.29) | 90.76(60.04 to 123.59) | 68.93(44.04 to 96.76) | 0.53 (0.09 to 0.99) | -1.01 (-1.48 to -0.54) |
| Republic of Moldova | 418.77(121.74 to 731.62) | 410.66(120.47 to 727.47) | 393.66(125.11 to 694.79) | 293.31(104.37 to 500.99) | -0.95 (-1.42 to -0.47) | -1.51 (-1.79 to -1.23) |
| Romania | 162.97(52.97 to 348.8) | 167.96(55.51 to 350.94) | 235.34(91.49 to 409.81) | 234.51(102.46 to 389.46) | 1.51 (1.13 to 1.9) | 1.43 (1.23 to 1.62) |
| Russian Federation | 623.68(139.35 to 1099.1) | 789.02(171.35 to 1383.48) | 542.34(142.83 to 881.47) | 332.6(107.45 to 565.19) | -0.96 (-1.93 to 0.02) | -2.99 (-3.7 to -2.28) |
| Rwanda | 80(38.26 to 137.2) | 73.91(35.93 to 124.57) | 57.73(28.68 to 96.92) | 61.2(30.09 to 104.9) | -2.29 (-2.68 to -1.89) | -1.57 (-1.87 to -1.27) |
| Saint Kitts and Nevis | 110.88(51.16 to 193.92) | 85.32(40.65 to 141.88) | 70.5(34.73 to 110.32) | 77.72(37.3 to 122.11) | -2.08 (-2.24 to -1.91) | -1.44 (-1.67 to -1.21) |
| Saint Lucia | 102.75(47.34 to 158.07) | 92.99(43.86 to 135.65) | 77.48(38.82 to 112.2) | 84.49(40.57 to 124.94) | -1.75 (-1.94 to -1.57) | -1.17 (-1.4 to -0.94) |
| Saint Vincent and the Grenadines | 103.51(47.18 to 166.81) | 109.74(51.63 to 166.3) | 106.56(52.31 to 160.92) | 114.19(55.89 to 172.89) | -0.21 (-0.39 to -0.04) | 0.13 (0 to 0.25) |
| Samoa | 87.38(38.8 to 155.29) | 102.68(44.46 to 174.8) | 114.07(47.99 to 194.97) | 113.92(49.31 to 198.88) | 1.55 (1.43 to 1.67) | 0.98 (0.81 to 1.15) |
| San Marino | 139.57(74.07 to 206.57) | 124.17(73.04 to 178.56) | 125.83(75.94 to 187.3) | 129.58(77.47 to 195.31) | -0.5 (-0.67 to -0.33) | -0.18 (-0.3 to -0.06) |
| Sao Tome and Principe | 75.96(34.71 to 148.44) | 96.48(43.77 to 187.7) | 104.74(49.75 to 195.28) | 108.36(52.02 to 200.84) | 1.76 (1.52 to 2) | 1.17 (0.97 to 1.36) |
| Saudi Arabia | 57.44(22.85 to 117.06) | 64.26(25.47 to 128.57) | 70.16(26.77 to 145.42) | 69.09(27.19 to 139.16) | 1.01 (0.95 to 1.07) | 0.66 (0.55 to 0.76) |
| Senegal | 107.47(54.69 to 190.65) | 105.22(55.47 to 182.87) | 124.93(68.41 to 204.04) | 132.26(73.25 to 215) | 1.08 (0.81 to 1.34) | 0.95 (0.81 to 1.09) |
| Serbia | 194.07(86.61 to 366.18) | 187.38(85.59 to 348.36) | 174.76(87.17 to 307.94) | 173.48(92.64 to 293.77) | -0.6 (-0.81 to -0.39) | -0.6 (-0.71 to -0.49) |
| Seychelles | 28.78(18.89 to 45.78) | 31.85(20.5 to 48.15) | 33.34(20.56 to 50.34) | 37.01(21.96 to 56.36) | 0.79 (0.66 to 0.93) | 0.67 (0.57 to 0.76) |
| Sierra Leone | 77.59(32.85 to 163.75) | 83.26(38.13 to 167.76) | 99.47(46.28 to 194.73) | 98.9(46.97 to 189.9) | 1.57 (1.38 to 1.75) | 1.15 (0.99 to 1.31) |
| Singapore | 79.48(37.03 to 150.23) | 67.95(33.12 to 121.62) | 62.47(33.04 to 105.51) | 55.47(29.61 to 90.68) | -0.91 (-1.02 to -0.8) | -1.13 (-1.22 to -1.03) |
| Slovakia | 269.66(94.74 to 533.35) | 222.87(84.12 to 431.77) | 241.02(103.49 to 423.38) | 234.87(109.77 to 392.27) | -0.23 (-0.6 to 0.14) | -0.13 (-0.32 to 0.05) |
| Slovenia | 275.4(121.86 to 435.1) | 283(154.98 to 397.97) | 203.75(123.74 to 285.76) | 178.51(110.26 to 252.46) | -1.71 (-2.19 to -1.22) | -2.19 (-2.46 to -1.91) |
| Solomon Islands | 122.2(58.9 to 233.96) | 142.64(65.24 to 263.55) | 157.38(71.55 to 299.92) | 173.18(79.51 to 318.36) | 1.43 (1.3 to 1.55) | 1.29 (1.2 to 1.37) |
| Somalia | 76.02(33.88 to 138.58) | 72.61(31.97 to 132.77) | 79.25(35.43 to 146.19) | 77.34(33.88 to 142.9) | 0.35 (0.19 to 0.52) | 0.31 (0.22 to 0.4) |
| South Africa | 65.21(31.18 to 105.26) | 94.29(45.93 to 148.26) | 110.68(58.79 to 164.14) | 97.75(53.42 to 141.53) | 2.82 (2.44 to 3.21) | 1.83 (1.47 to 2.2) |
| South Sudan | 70.09(34.2 to 121.45) | 67.64(33.99 to 114.63) | 71.83(35.51 to 123.4) | 71.1(35.56 to 120.83) | 0.12 (-0.03 to 0.26) | 0.16 (0.08 to 0.23) |
| Spain | 255.03(136.19 to 365.23) | 212.68(122.09 to 297.06) | 171.21(105.61 to 232.22) | 156.77(99.39 to 215) | -1.98 (-2.04 to -1.93) | -1.92 (-2.01 to -1.82) |
| Sri Lanka | 32.87(19.63 to 53.74) | 37.86(22.48 to 59.52) | 45.31(25.12 to 66.79) | 44.29(23.11 to 68.45) | 1.89 (1.67 to 2.1) | 1.48 (1.29 to 1.68) |
| Sudan | 67.62(27.06 to 151.49) | 62.33(25.71 to 140.44) | 59.8(24.76 to 133.04) | 60.04(24.6 to 127.59) | -0.68 (-0.73 to -0.63) | -0.46 (-0.53 to -0.4) |
| Suriname | 84.76(36.63 to 156.44) | 79.65(35.77 to 137.33) | 87.91(41.95 to 141.78) | 97.18(47.59 to 156.23) | -0.1 (-0.58 to 0.39) | 0.56 (0.26 to 0.86) |
| Sweden | 282.25(99.68 to 443.91) | 222.6(98.13 to 337.3) | 185.62(99.54 to 268.19) | 167.7(94.72 to 238.37) | -2.02 (-2.11 to -1.92) | -1.86 (-1.95 to -1.78) |
| Switzerland | 185.95(82.21 to 295.25) | 155.54(80.65 to 235.6) | 124.39(70.18 to 176.51) | 115.36(69.18 to 162.85) | -2.12 (-2.2 to -2.05) | -1.89 (-1.99 to -1.8) |
| Syrian Arab Republic | 85.82(30.37 to 201.23) | 85.93(32.42 to 190.63) | 74.22(27.76 to 167.9) | 73.91(27.43 to 165.52) | -1.11 (-1.34 to -0.88) | -0.84 (-0.98 to -0.7) |
| Taiwan (Province of China) | 57.44(29.51 to 92.18) | 69.41(38.21 to 100.94) | 68.16(39.32 to 100.8) | 73.98(41.98 to 109.12) | 0.83 (0.55 to 1.11) | 0.55 (0.4 to 0.71) |
| Tajikistan | 152.95(54.81 to 327.69) | 165.54(58.66 to 361.27) | 212.21(80.99 to 444.08) | 236.12(96.23 to 476.78) | 0.96 (0.54 to 1.39) | 1.59 (1.31 to 1.87) |
| Thailand | 16.95(12.27 to 22.85) | 18.59(13.22 to 25.37) | 16.75(10.05 to 24.58) | 20.42(10.8 to 31.9) | -0.48 (-0.74 to -0.21) | 0.1 (-0.15 to 0.35) |
| Timor-Leste | 19.66(13.34 to 29.7) | 20.07(12.7 to 30.46) | 23.64(14.88 to 36.86) | 29.17(18.26 to 46.14) | 0.79 (0.6 to 0.99) | 1.54 (1.32 to 1.77) |
| Togo | 84.08(37.94 to 169.04) | 101.25(47.68 to 190.04) | 112.35(55.29 to 214.51) | 109.49(53.17 to 200.95) | 1.57 (1.44 to 1.7) | 0.97 (0.79 to 1.15) |
| Tokelau | 81(36.7 to 140.71) | 94.65(42.65 to 159.8) | 104.15(45.85 to 172.07) | 107.21(46.72 to 177.66) | 1.38 (1.28 to 1.48) | 1 (0.89 to 1.12) |
| Tonga | 72.45(34.45 to 112.28) | 83.28(40.12 to 130.75) | 88.54(41.07 to 138.19) | 93.09(41.04 to 143.73) | 1.26 (1.07 to 1.45) | 0.96 (0.84 to 1.09) |
| Trinidad and Tobago | 175.92(82.53 to 279.95) | 165.51(80.25 to 259.96) | 138.92(69.41 to 206.3) | 143.98(66.9 to 220.08) | -1.18 (-1.39 to -0.97) | -1.09 (-1.26 to -0.92) |
| Tunisia | 52.1(20.38 to 113.3) | 56.63(21.99 to 123.26) | 58.14(24.07 to 120.38) | 58.14(23.6 to 115.69) | 0.37 (0.27 to 0.47) | 0.28 (0.22 to 0.33) |
| Turkey | 65.51(27.37 to 126.89) | 47.29(18.96 to 92.57) | 48.8(21.1 to 84.97) | 46.93(20.68 to 82.5) | -2.14 (-2.64 to -1.64) | -0.95 (-1.35 to -0.55) |
| Turkmenistan | 286.55(77.03 to 606.38) | 338.32(91.34 to 747.99) | 309.56(100.17 to 608.02) | 336.54(120.83 to 635.85) | 0.31 (-0.3 to 0.92) | -0.14 (-0.46 to 0.19) |
| Tuvalu | 96.62(44.23 to 169.83) | 112.78(48.64 to 189.29) | 122.43(54.66 to 207.61) | 126.11(54.63 to 218.6) | 1.29 (1.19 to 1.39) | 0.94 (0.83 to 1.05) |
| Uganda | 58.93(29.12 to 99.6) | 75.4(37.33 to 125.34) | 71.32(37.36 to 116.79) | 71.69(36.66 to 119.52) | 0.99 (0.62 to 1.37) | 0.4 (0.16 to 0.64) |
| Ukraine | 125.16(38.42 to 298.84) | 155.01(42.97 to 394.76) | 183.5(50.22 to 440.9) | 209.55(57.74 to 481.84) | 2.1 (1.7 to 2.49) | 1.59 (1.32 to 1.86) |
| United Arab Emirates | 176.02(82.94 to 328.97) | 192.19(92.45 to 349.89) | 156.1(74.73 to 269.98) | 109.33(49.76 to 193.04) | -0.26 (-0.69 to 0.16) | -1.93 (-2.44 to -1.43) |
| United Kingdom | 331.67(114.43 to 522.13) | 255.21(111.27 to 382.35) | 200.55(115.18 to 286.25) | 201.02(123.01 to 281.82) | -2.51 (-2.57 to -2.46) | -1.93 (-2.13 to -1.73) |
| United Republic of Tanzania | 58.53(28.96 to 98.73) | 59.45(29.95 to 100.34) | 64.23(32.25 to 109.92) | 67.43(33.86 to 116.52) | 0.5 (0.39 to 0.61) | 0.65 (0.58 to 0.72) |
| United States of America | 335.97(144.54 to 510.15) | 315.88(149.84 to 462.2) | 286.32(162.68 to 408.11) | 278.41(160.25 to 392.78) | -0.65 (-0.74 to -0.57) | -0.73 (-0.79 to -0.67) |
| United States Virgin Islands | 84.5(38.28 to 157.48) | 86.42(40 to 150.59) | 102.45(51.1 to 174.15) | 101.52(48.95 to 173.11) | 0.78 (0.49 to 1.07) | 0.8 (0.65 to 0.95) |
| Uruguay | 173(77.94 to 298.37) | 143.15(71.08 to 231.98) | 135.33(76.11 to 203.98) | 146.28(88.09 to 208.55) | -1.28 (-1.45 to -1.12) | -0.63 (-0.83 to -0.42) |
| Uzbekistan | 208.73(62.55 to 436.12) | 323.02(101.61 to 691.89) | 431.29(149.87 to 893.68) | 423.99(159.66 to 854.42) | 3.48 (3.1 to 3.87) | 2.51 (2.15 to 2.87) |
| Vanuatu | 73.08(33.94 to 149.26) | 89.28(40.39 to 172.7) | 104.94(45.75 to 200.78) | 112.59(48.87 to 209.83) | 1.55 (1.37 to 1.73) | 1.45 (1.34 to 1.55) |
| Venezuela (Bolivarian Republic of) | 86.33(39.26 to 162.62) | 75.6(33.31 to 137.23) | 85.23(40.45 to 148.24) | 98.97(47.67 to 172.66) | -0.29 (-0.7 to 0.12) | 0.52 (0.23 to 0.82) |
| Viet Nam | 10.24(8.58 to 12.4) | 9.52(8.15 to 11.51) | 11.62(10.04 to 13.88) | 16.56(12.83 to 21.35) | 0.47 (0.14 to 0.81) | 1.82 (1.41 to 2.22) |
| Yemen | 62.65(26.38 to 141.63) | 56.11(24.2 to 125.6) | 52.78(22.73 to 112.67) | 54.27(23.86 to 114.95) | -0.86 (-0.95 to -0.77) | -0.54 (-0.65 to -0.43) |
| Zambia | 75.04(35.62 to 129.57) | 86.35(40.9 to 149.24) | 80.52(37.67 to 137.02) | 74.4(36.08 to 124.77) | 0.18 (-0.1 to 0.45) | -0.34 (-0.53 to -0.15) |
| Zimbabwe | 52.92(24.14 to 92.78) | 65(31.33 to 111.66) | 84.63(38.05 to 145.9) | 83.28(37.38 to 144.98) | 2.87 (2.58 to 3.16) | 2.07 (1.8 to 2.35) |

ASDR, age-standard DALYs rate; DALYs, disability-adjusted life years; EAPC, estimated annual percentage change.
